# Understanding Nanocellulose–Water Interactions: Turning a Detriment into an Asset

**DOI:** 10.1021/acs.chemrev.2c00611

**Published:** 2023-02-01

**Authors:** Laleh Solhi, Valentina Guccini, Katja Heise, Iina Solala, Elina Niinivaara, Wenyang Xu, Karl Mihhels, Marcel Kröger, Zhuojun Meng, Jakob Wohlert, Han Tao, Emily D. Cranston, Eero Kontturi

**Affiliations:** † Department of Bioproducts and Biosystems, Aalto University, EspooFI-00076, Finland; ‡ Wallenberg Wood Science Centre (WWSC), Department of Fibre and Polymer Technology, School of Engineering Sciences in Chemistry, Biotechnology and Health, KTH Royal Institute of Technology, 10044Stockholm, Sweden; § Department of Wood Science, University of British Columbia, Vancouver, British ColumbiaV6T 1Z4, Canada; ∥ Department of Chemical and Biological Engineering, University of British Columbia, Vancouver, British ColumbiaV6T 1Z3, Canada; ⊥ Laboratory of Natural Materials Technology, Åbo Akademi University, TurkuFI-20500, Finland; # Wenzhou Institute, University of Chinese Academy of Sciences, Wenzhou325001, China

## Abstract

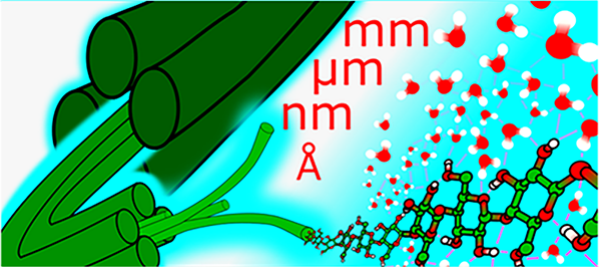

Modern technology has enabled the isolation of nanocellulose from plant-based fibers, and the current trend focuses on utilizing nanocellulose in a broad range of sustainable materials applications. Water is generally seen as a detrimental component when in contact with nanocellulose-based materials, just like it is harmful for traditional cellulosic materials such as paper or cardboard. However, water is an integral component in plants, and many applications of nanocellulose already accept the presence of water or make use of it. This review gives a comprehensive account of nanocellulose–water interactions and their repercussions in all key areas of contemporary research: fundamental physical chemistry, chemical modification of nanocellulose, materials applications, and analytical methods to map the water interactions and the effect of water on a nanocellulose matrix.

## 1 Introduction

The term nanocellulose refers to anisotropic nanoparticles that can be isolated from, in most cases, a processed plant cell wall. While the early accounts of nanocellulose originate from the mid-20th century, the first decade of the 21st century saw process-related advances that enabled a more facile and efficient isolation of nanocellulose for materials construction.^1^ This development in nanocellulose preparation and its applications has unleashed an unprecedented scientific interest in cellulose-based materials over the past 15 years.^2−14^ Although much of the attention has focused on utilizing nanocellulose as a building block in new functional materials, the fundamental progress in cellulose science stemming from nanocellulose research has also been remarkable.^15−19^

This review presents a comprehensive, critical coverage of a topic which is dominant in both fundamental aspects and materials applications of nanocellulose: cellulose–water interactions. The presence of water or humidity in cellulose-based materials is often seen as a detriment. Everyone knows what happens to paper when you immerse it in water: it disintegrates and loses its mechanical strength. Nanocellulose is made of much smaller entities than pulp fibers, and because of its high surface area, it takes up more water and the effect is even more drastic. As high strength coupled with low density is one of the main assets of nanocellulose, the strength loss in water is a major issue. In general, water is seen as a nuisance, and the ensuing problems are being tackled with “brute force” such as chemical hydrophobization and the like. Quantification, localization, and influence of water within a cellulose matrix has also been subject to a number of analytical challenges throughout the history,^20^ and it continues to be that way.^21^

The purpose of this review is to point out how the presence of water can be beneficial as well as detrimental in nanocellulose-based systems, processes, and materials: isolation, chemical modification, biomedical templates, responsive hydrogels, sensors, smart emulsions, and so forth. Although intuitively utilized since the ancient times, for example, Egyptians exploiting wood swelling in water to seal leaking joints in their boats, the systematic usage of water interactions has started to emerge only within the past few years.

The specific response to water has its roots in the amphiphilic nature of cellulose and its native crystallite structure. The literature on fundamental aspects of cellulose–water interactions spans roughly one century, albeit with a dramatic upsurge during the past decade, fully covered in this review. Understanding the different “types” of bound water and how to measure them, is another crucial step to exploiting nanocellulose–water interactions, and as such, we extensively discuss analytical tools including modeling and experimental approaches to elucidate the relationships between water and nanocellulose.

A number of studies and reviews focus on the interactions of water and other natural polymers such as chitin^22,23^ and collagen^24,25^ in the literature. To our knowledge, a comprehensive review on such materials to the extent of the scope of this review has not been published. In addition to presenting fundamentals and characterization, this review focuses on new nanocellulose-based materials which may suffer (but just as well benefit) from the presence of water. We see this as a vital approach in the current research environment where “green” solutions to chemicals and materials are intensively sought after. Accepting (and taking advantage of) the presence of water and the predictability of processing nanocellulose in water is also important when we consider replacing nonaqueous solvents with water in striving toward a more sustainable society. Combined with the comprehensive nature of our approach, spanning fundamentals and analytics, the all-inclusive take on the role of water interactions in nanocellulose production, modification, and applications is what distinguishes this review from other recent reviews that touch on the subject of cellulose–water interactions.^3,9,19,26−29^

## 2 Fundamentals of Cellulose– and Nanocellulose–Water Interactions

### 2.1 Overview of the Properties of Water

Water is usually considered as either a solvation agent or a suspension medium in nanocellulose related research, and the complexity of water is often overlooked. To understand nanocellulose–water interactions, it is pivotal to briefly review some of the relevant characteristics and main features of water. To limit the scope of this discussion, we focus on liquid water at moderate temperatures and pressures. For more comprehensive reviews of water and its properties in more extreme conditions, we guide the reader to relevant publications in the field.^30−33^ The complex behavior of water results from a combination of hydrogen bonding, dissociation behavior, and the complex structural dynamics, influenced by temperature, pressure, and its interaction with the interfaces and other molecules. As we approach this topic, it should be noted that much controversy still exists regarding the detailed mechanisms and dynamics of water behavior at this level.^34^ Also, the developments in the simulation of water properties and dynamics^35−37^ as well as experimental techniques^38,39^ have been recently reviewed elsewhere.

IUPAC defines a hydrogen bond as “an attractive interaction between a hydrogen atom from a molecule or a molecular fragment X–H in which X is more electronegative than H, and an atom or a group of atoms in the same or a different molecule, in which there is evidence of bond formation”.^40^ This definition leaves the mechanism of this attractive interaction purposefully open, as no single physical force can be found to be responsible for the phenomena we observe as hydrogen bonding.^40^ Indeed, the hydrogen bond is either described as a sum of several forces such as electrostatic interactions, polarization, induction interactions between multipoles, charge-transfer-induced covalency, or an independent interaction with unidentified origin.^40^ The consensus in the scientific community is that there are three-dimensional dynamics and a random network of hydrogen-bonded molecules in liquid water in which the hydrogen bonds are continuously broken and reformed on the time scale of femtoseconds to picoseconds.^41^ Thus, there is a broad distribution of possible energies and an indefinitely high number of molecules involved in the hydrogen bonding network of bulk liquid water.^42^ This means that assigning a specific, singular, hydrogen bonding energy to a liquid water system would be misleading. Moreover, as water can be both an acceptor and a donor of multiple hydrogen bonds, a distribution of acceptor–donor states exists.^42^ The 2 acceptor −2 donor model, resulting in tetrahedral molecular ordering, seems to be the dominant structure on average.^43^ Due to the random structuring of hydrogen bonds, a water cluster can be identified as a subgroup of water molecules, which form comparatively stable substructures in the time frame of hydrogen bond formation and dissociation. These water clusters can influence the structuring of hydrogen bonds in the surrounding medium outside the cluster.^44^ Although their very existence is not that controversial, the exact structure, lifetime, and effects that clusters have on the surrounding water medium are still under debate. In addition to water clustering, other phenomena characterized by the hydrogen water dynamics include proton hopping (the exchange of protons between neighboring water molecules)^45^ and changes in the hydrogen bond structure of water around OH^–^ and H_3_O^+^ ions.^46^ Fluctuations in hydrogen bond networks are experimentally accessible by computer simulations,^47^ albeit some spectroscopic methods also allow for probing of the phenomena occurring on these time scales.^37^ For a more detailed discussion on how the various ways the hydrogen bond energy of water has been approximated and investigated we suggest a book chapter by Chaplin^48^ and a review by Cisneros et al.^35^ for a more technical-oriented approach.

In addition to water structuring in molecular clusters, the hydrogen bond network of water is altered due to interactions with interfaces or other molecules resulting in the reordering of water molecules. Interesting examples of this phenomenon are the structuring of water in the presence of solutes, at the surface of water-dispersed colloids/particles, and even at hydrophobic surfaces. We address the structuring of water around nanocellulosic materials in section 3.1 when the amphiphilic nature of nanocellulose is discussed.

### 2.2 Cellulose in Nature

#### 2.2.1 Origin and Basic Crystalline Structure of Cellulose

Cellulose is a semicrystalline polysaccharide composed of β-1,4-linked d-anhydroglucopyranose units (C_6_H_10_O_5_), and it is biosynthesized from glucose through a uridine diphosphate glucose intermediate by all higher-order plants,^49^ green algae,^50^ as well as some specific marine animals (tunicates)^51^ and certain bacteria (namely those belonging to the genera Acetobacter, Rhizobium, Agrobacterium, and Sarcina).^52^ Cellulose is the most ubiquitously present natural polymer in both land and marine ecosystems and functions as a highly effective natural carbon sink in terrestrial ecosystems turning over almost 3.6 gigatons of carbon annually.^53^ The majority of cellulose is found in higher-order plants, and it typically accounts for 40–50% of the mass of wood material depending on the species of the plant source.^54^

**Figure 1:**
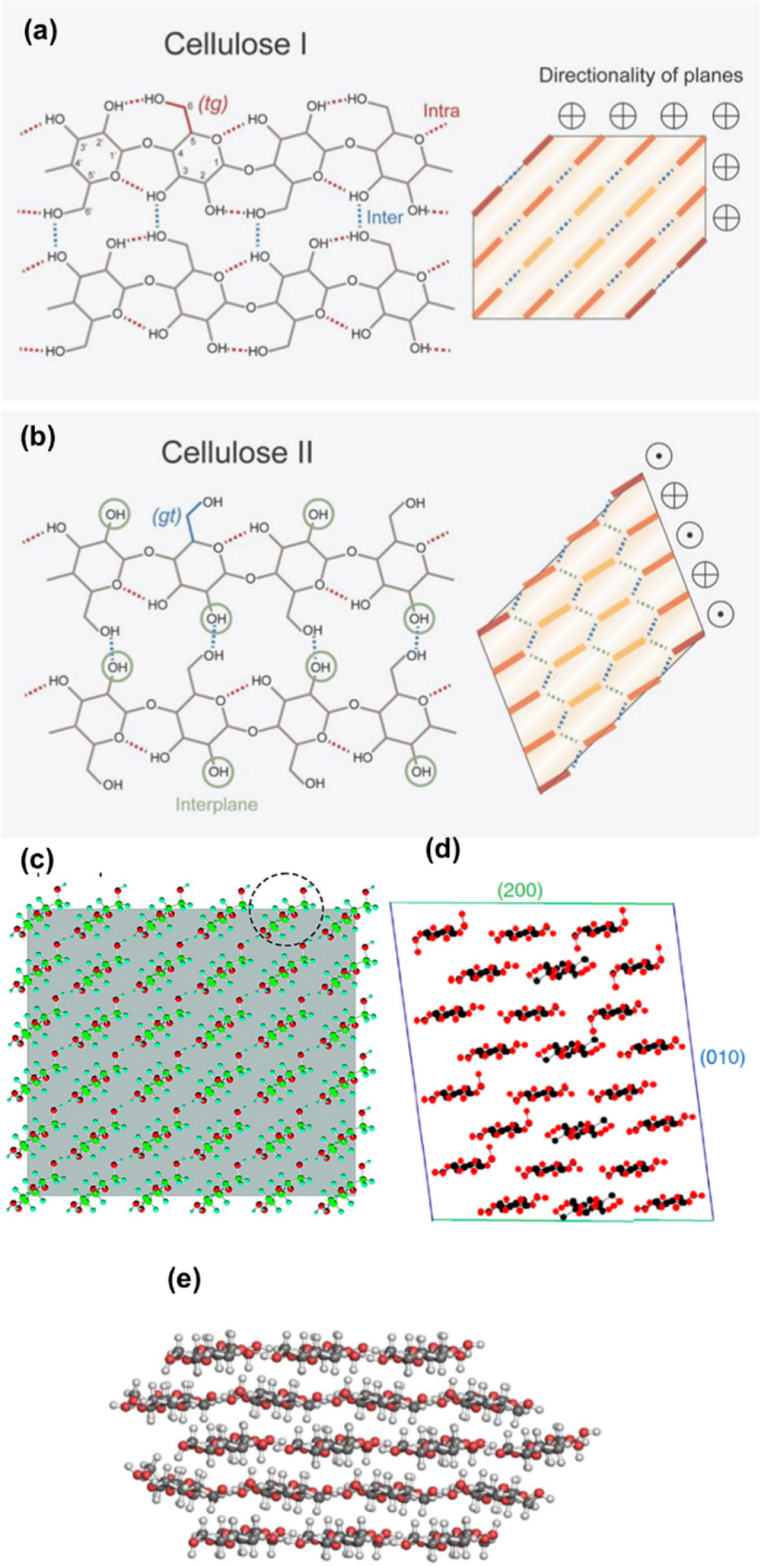
(a,b) Hydrogen bonding network in cellulose I and II. (a,b) Reproduced from ref (59) under the terms of the CC-BY Creative Commons Attribution 4.0 International license (CC-BY 4.0). Copyright 2021 Springer Nature. (c–e) Different chain models for cellulose (c) the much debated 6 × 6 chain model. (c) Adapted with permission from ref (60). Copyright 2010 American Chemical Society. (d) 6 × 4 chain model. (d) Reproduced under the terms of PNAS exclusive License to Publish.^61^ (e) 18-chain model (34443 form). (e) Reproduced from ref (62) under the terms of the CC-BY. Copyright 2018 Springer Nature.

Cellulose biosynthesis is carried out by the cellulose synthase complex or terminal complex (TC),^55^ where simultaneously upon their synthesis the cellulose polymer chains are assembled into higher-order structures known as microfibrils, which are the smallest supramolecular units of cellulose in nature. Microfibrils are semicrystalline, slender threads that form the structural scaffold of the plant cell wall. In the native cellulose crystal, sheets formed by hydrogen bonding are stacked on top of each other through interplanar van der Waals forces.^56^ Within the sheets, the intramolecular hydrogen bonds in the native cellulose I crystal are between HO(3)–HO(5) and HO(2)–HO(6), whereas the major intermolecular bond forms between HO(3) and HO(6) (Figure 1a for cellulose I and Figure 1b for cellulose II). Overall, the hydrogen bond energy of cellulose ranges from 17 to 30 kJ mol^–1^, and the intermolecular hydrogen bond energy is approximated to be around 20 kJ mol^–1^. In cellulose I, for example, the density of hydrogen bonds is approximately 3.7 × 10^18^ m^–2^ along the 1(−1)0 crystallographic plane.^57^

The widths and the shapes of the crystal, which determine the width of the microfibril, differ according to the cellulose source. As a rule of thumb, the higher the plant has climbed on the evolutionary ladder, the thinner the crystal. Trees have the thinnest crystals (∼3 nm), while algae have the widest (>20 nm). This observation can be rationalized by taking into account that the crystallinity and order in the higher plant cell walls are optimized to find a perfect balance between strength and flexibility and to ensure the structural integrity of the organism depending on their growth and environmental condition (e.g., wind, water availability).^58^

The number of cellulose chains that make up the cellulose crystallite in a microfibril is dependent on the source of the cellulose, and it is still a matter of debate among the cellulose community. For instance, in the case of wood cellulose, it was traditionally accepted that each TC synthesizes microfibrils consisting of 36 cellulose polymer chains (i.e., a 6 × 6 chain cross-section, Figure 1c). Later, however, Jarvis and co-workers suggested that 24 chains make up the crystal (Figure 1d),^61^ and more recently, models for 18 chain crystals have gained ground (Figure 1e).^63^ These 24 and 18 chain models appear currently more accepted within crystallographers than the traditional 36 chain model. Regardless of the exact number of chains making up the microfibril, it is important to understand that cellulose in nature exists exclusively in the form of microfibrils and it is never found in nature as single polymer chains or in a fully amorphous form.

Native crystalline cellulose, also referred to as cellulose I, is assembled with the cellulose polymer chains running parallel to one another, and it exists as two different crystal structures (i.e., polymorphs): triclinic I_α_ or monoclinic I_β_.^64^ Primarily, the crystal structure of cellulose is governed by the conformation of the TC, which in turn affects the morphology of the resulting microfibril.^50^ Linear TCs, for example, mostly produce I_α_ rich cellulose and relatively wide microfibrils with high degree of crystallinity, while rosette-shaped TCs produce thin, I_β_-rich cellulose microfibrils.^65,66^ Tsekos et al. showed a rough, but indisputable, correlation between the shape of the TC and the resulting cellulose microfibril.^67^ It is important to note, however, that both polymorphs coexist in all cellulose and their ratio varies depending on the cellulose source.^68^ Cellulose I_β_ is dominant in higher-order plants and tunicate-synthesized cellulose, while cellulose I_α_ is the main component of celluloses synthesized by algae and bacteria.

Two alternative hypotheses have been proposed to explain the simultaneous presence of the I_α_ and I_β_ forms of cellulose. The first is that cellulose I_α_ is synthesized by a different type of TC from cellulose I_β_, whereas the second hypothesis is that the different cellulose polyomorphs result from events that occur after the synthesis of the cellulose polymer chains. For example, it has been shown that bending can interconvert the crystalline forms of cellulose I and that their ratio is very sensitive to the angle through which the microfibril is bent.^69^

Besides the native crystalline form, different cellulose polymorphs exist, in which the crystalline structure of cellulose I_α_ and I_β_ have been altered through physicochemical treatments. Cellulose II can be prepared by reorganizing the hydrogen bonding network such that the cellulose chains run antiparallel to each other in the crystal structure using either a mercerization process where cellulose I is swollen in the presence of NaOH or through the regeneration (i.e., solubilization and subsequent recrystallization) of cellulose (Figure 1b).^70^ Cellulose I readily converts into cellulose II in an irreversible process, as cellulose II is thermodynamically more stable than cellulose I.^71^ A third cellulose polymorph called cellulose III can also be reversibly prepared by exposing cellulose I or II to liquid ammonia or certain diamines.^72^ However, cellulose I and II arguably garner the most research interest because of their biological, industrial, and scientific relevance, and cellulose I is overwhelmingly the most dominant polymorph in all nanocellulose constructs.

#### 2.2.2 Cellulose Morphology and the Hierarchical Structure of the Plant Cell Wall

**Figure 2:**
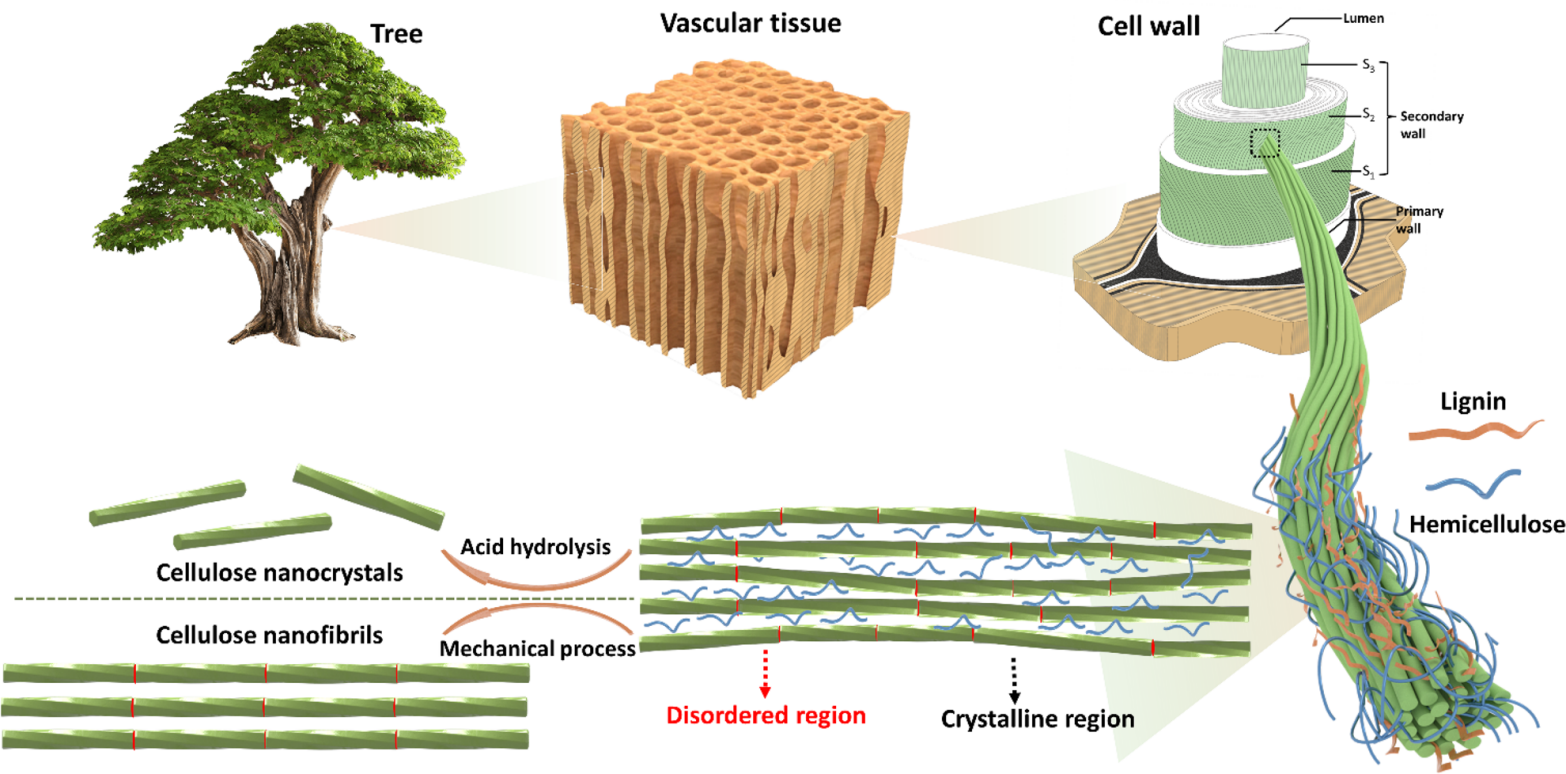
Schematic representation of the plant cell wall and cellulose fiber structure. CNFs and CNCs are extracted from cellulose fibers using mechanical process and chemical methods (oxidation or acid hydrolysis), respectively.

While cellulose microfibrils are predominantly crystalline, there is direct evidence showing that the microfibril structure in higher plants has regions of disordered cellulose distributed along their length (Figure 2).^49,73^ The length of the crystalline portions between these disordered regions is primarily governed by the source of the cellulosic material, and this is often depicted using the so-called fringed-fibrillar model.^49,56,74−76^ For example, the length of the crystalline regions in cotton-derived cellulose is on average 125 nm (i.e., a degree of polymerization (DP) of 250), whereas that of tunicate cellulose can be as high as 3 μm (DP = 6000).^77^ The inherent length of the crystalline portions of the cellulose structure is linked to the leveling-off degree of polymerization (LODP), corresponding to the DP at which the cellulose structure become inaccessible for further degradation when exposed to strong mineral acids or oxidizing agents at semidilute concentrations.^78,79^

Isolated native cellulose often gives crystallinity index values between 60% and 90% when analyzed by X-ray diffraction or solid-state NMR spectroscopy. However, such values do not directly indicate that, for example, 70% of the cellulose in the microfibrils would be crystalline and the remaining 30% would be disordered or “amorphous”. In comparison to the crystalline regions, the disordered regions are reportedly very short: accounts of 1–2 nm and 3–6 nm have been proposed,^73,80^ i.e., they are more like defects in the crystallite rather than bulky amorphous regions as they are often schematically depicted in traditional literature. Much of the response of the disordered cellulose in analytics likely comes from the microfibril surface simply because the chains there have higher degrees of freedom. As a result, the systematically higher crystallinity reported for algae^50^ or tunicates^51^ comes largely from the fact that their microfibrils, and therefore their crystallites, are wider than those in higher plants (i.e., there are fewer surface cellulose chains).^1,81^ Cellulose sample preparation prior to crystallinity measurements can also affect the degree of crystallinity values obtained, at least within a few percent range. Certainly, the frequency of the disordered regions also plays a role here, but we believe that the crystallite width is a more significant factor.

Furthermore, while nearly all studies assume that the disordered cellulose regions are a part of natural microfibrils, some reports have suggested that these regions of disorder are in fact a result of the processes involved in cellulose isolation. For example, Atalla et al. put forward that native celluloses are irreversibly transformed and develop the semicrystalline character upon isolation at elevated temperatures.^82^ These issues are still under debate. Nonetheless, the semicrystalline nature of cellulose is a fact of processed cellulosic materials, such as virtually all nanocellulose types, and it adds to their complexity as areas of high order are less susceptible to chemical and biological attack.^83^

The role of disordered regions in water-induced swelling of cellulosic materials is not straightforward. There is a common consensus that crystalline cellulose is impenetrable by water.^84^ However, the disordered regions may be somewhat “accessible”, but they are certainly not “swollen” by water because of their short, defect-like texture. It has never been shown that the length of a microfibril would increase when immersed in water. However, small angle neutron scattering data have shown that a somewhat higher concentration of D_2_O molecules can be observed in the vicinity of the disordered segments.^85^

Another often misunderstood fundamental issue with cellulose–water interactions is hydrogen bonding. Despite the undisputable importance of hydrogen bonding between water molecules and nanocellulose at the interface in governing the characteristics and properties of cellulose dispersions, we would like to echo the conclusions of Wohlert et al. in their critical review on the general role of hydrogen bonding in nanocellulose structure and properties as a material. They conclude that hydrogen bonding is one interaction among several, and its relative contribution to the nanocellulose properties is highly dependent on the specific conditions and cannot easily be determined by intuition or even by analysis.^57,59^

In any case, the combination of the insolubility of cellulose in water with its ability to interact with water are fundamentally important to sustaining its structural integrity. Nature has utilized this feature of cellulose to engineer the incredible structure that is the plant cell wall. The plant cell wall is made up of three distinct regions: (i) the primary cell wall, (ii) the secondary cell wall (made up of three layers), and (iii) the middle lamella. Within the primary and secondary plant cell walls, cellulose microfibrils are further assembled into microfibril bundles and form a composite network structure with the two other main plant cell wall components, namely hemicellulose and lignin (Figure 2).^86−88^

Within the plant cell wall, the microfibrils (and their bundles) are orientated depending on which layer of the cell wall they exist in (Figure 2). In the primary cell wall, the microfibrils form very thin oriented layers with different orientations to one another, forming the impression of a random network.^89,90^ On the other hand, the highly aligned microfibrils have a unique microfibril angle in each of the three layers of the secondary cell wall. This level of natural hierarchical engineering provides plants with the necessary flexibility required for growth and swelling in the presence of water while ensuring the plant has sufficient axial stiffness.^91,92^ When discussing the swelling of cellulosic materials in water, one must remember that it is never the individual cellulose microfibril that swells, it is always a scaffold where bulk water clusters between the microfibrils. As a result of axial microfibril orientation in the secondary wall, the plant fibers always swell copiously in a radial direction, but their length of increase when immersed in water is negligible.^93^

Plant fibers are always swollen by water as the major component in their native growth environment, which imparts the plants with necessary flexibility. It was earlier proposed that water is held in a microporous gel of hemicelluloses and lignin distributed as fine platelets within a cellulose skeleton.^94^ Yet the water content is strictly controlled by the presence of more hydrophobic lignin in the cell wall. Conventional pulping process including beating and bleaching (delignification) is often undertaken when chemically processing plant fibers to, for example, pulp for paper production. Rheological properties of fiber–water suspensions where water acts as a suspension matrix are of critical importance in many of papermaking process from beating, screening, fractionation, dispersion flow in headbox, sheet forming, and dewatering.^95^ In general, delignified fibers make up a strong network because the fibers are able to form inter- and intrahydrogen bonds with each other due to cellulose surface exposure after lignin removal.^93^ Two general water transport mechanisms including diffusion and capillary flow were suggested in cellulosic materials.^96^ If the fiber network is exposed to water, however, the fibers and the network lose their integrity and mechanical strength because lignin is no longer there to obstruct the water adsorption to cellulose surface due to pore flow accompanied by a surface “hopping” mechanism.^96,97^ A delignified fiber is also prone to irreversible loss of porosity due to drying. Pores between the cellulose microfibrils are filled with water in a swollen state and the pores disappear due to capillary forces as the water is removed. When the fiber is re-exposed to water, the pores reappear but not to the same extent as before drying. This decrease in the swelling capability of a fiber is referred to as hornification, and it has genuine practical implications not only in papermaking and paper recycling but also in nanocellulose production.^98−103^ Hornification is often (usually without explicit evidence) attributed to “irreversible hydrogen bond formation between neighboring microfibrils upon drying”. To our perception, a more likely reason could be hydrophobic interactions where the hydrophobic sites of the cellulose crystals in microfibrils partially aggregate. Such hydrophobic bonding is less likely to be cleaved by water upon re-exposure than hydrogen bonding. We must acknowledge, however, that several accounts refer to co-crystallization or association of hydrophilic sites as a culprit for hornification, with obvious involvement of (also) hydrogen bonding between the microfibrils.^104,105^ The issue remains unsettled within the community. Hornification or similar phenomena with microfibril aggregation have even been reported to occur in air-dried cotton fibers upon exposure to HCl vapor,^106^ a preliminary phase in one type of nanocellulose isolation procedure.^107^ Moreover, the loss in swelling capability is not restricted to fully delignified samples and it has also been observed to an extent for lignin-containing samples such as wood and mechanical pulp.^108,109^ The situation is further complicated by the fact that hornification is known to be at least partially reversible with introduction of mechanical force in the system, a process referred to as beating in the papermaking sciences.^110^ In addition, the response of the dimensional behavior of paper to relative humidity changes is a reflection of individual cellulose fiber changes in macroscale manner.^111^ The torsional response, i.e., twist, in drying of a collapsed fiber is a function of the microfibril angle, the fiber length, and the factional linear shrinkage across the microfibrils, which is closely related to the nanoscale twist of nanocellulose (will be explicitly discussed in section 2.3 and section 4.1). All in all, drying of wood and other native specimen is a more complex affair with a series of structural rearrangements taking place, involving physical deformations such as bending, buckling, or twisting of the fibrous cellulose bundles.^112−115^

### 2.3 From Cellulose to Nanocellulose

#### 2.3.1 Types of Nanocellulose

**Figure 3:**
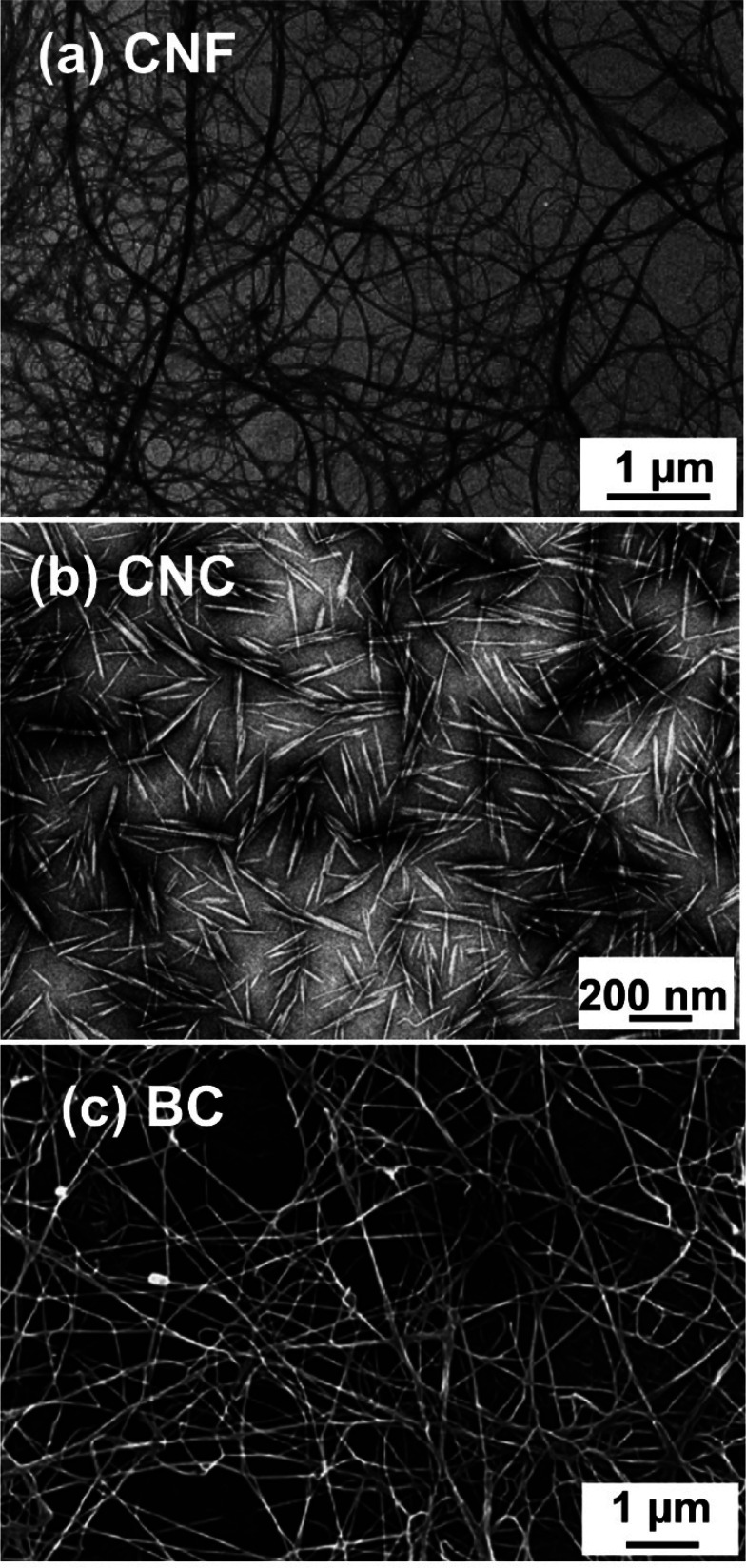
Typical appearance of nanocellulose observed by TEM (a,b) and SEM (c). (a) CNF^122^ and (b) CNC.^123^ (a) Adapted with permission from ref (122). Copyright 1998 John Wiley and Sons. (b) Adapted with permission from ref (123). Copyright 1991 The Royal Society of Chemistry. (c) SEM image of BC membrane.^124^ (c) Adapted with permission from ref (124). Copyright 2008 Elsevier.

Nanocellulose refers to cellulosic materials which have at least one dimension in the nanoscale. A vast majority of nanocellulose consists of anisotropic nanoparticles with varying aspect ratios although spherical nanocelluloses have also been reported.^116,117^ Nanocellulose, namely cellulose nanofibrils (CNFs), cellulose nanocrystals (CNCs), and bacterial cellulose (BC), have garnered a vast amount of research attention due to their incredible versatility. Their high aspect ratios, high surface area to volume ratios, abundance of surface hydroxy groups, and high strength enable them to be used in a wide variety of potential applications.^118,119^ While all nanocellulose grades exhibit the aforementioned properties, CNFs and BC are distinctly different materials to CNCs, as attested visually in Figure 3.^120,121^

CNFs are essentially isolated cellulose microfibrils (Figure 2 and Figure 3a). In consequence, they are flexible and semicrystalline threads, with diameters in the nanoscale (average width of 2–50 nm) but lengths in the micrometer range (1–15 μm).^51,102^ A series of chemical (e.g., a 2,2,6,6-tetramethylpiperidin-1-yl)oxyl (TEMPO)-mediated oxidation^125^) and/or enzymatic pretreatments (e.g., cellulases),^126^ followed by substantial mechanical defibrillation,^127^ are necessary to liberate CNFs from their biological matrix, i.e., the plant fiber, given the strong interactions between cellulose microfibrils and the tightly knit hierarchical structure of the cell wall.^128^ During the mechanical defibrillation, high shear forces are applied to isolate single CNFs which have dimensions dependent on both the isolation method and the source of the cellulose. The surface charge of CNFs depends on the amount of residual hemicellulose, namely xylan with methylglucuronic acid groups, and the choice of pretreatment. Specifically, the CNFs prepared by TEMPO-mediated oxidation pretreatment (TOCNF) carry very high charge densities, with approximately every second anhydroglucose unit on the surface bearing a carboxylic group when the maximum degree of oxidation is applied. Full details on CNF isolation can be found in the relevant reviews.^102,129^ TEMPO-mediated oxidation is also the only pretreatment method that manages to truly individualize microfibrils into CNFs. With other isolation methods, the CNFs always consist of (at least partially) microfibrillar bundles.^129^

CNCs (sometimes called nanowhiskers) on the other hand, are highly crystalline, rod-shaped nanoparticles, with widths between 3 and 50 nm and lengths ranging from 50 nm to 10 μm (Figure 3b) depending on the cellulose source.^130^ The most common source for CNCs is cotton because of the wide availability and relative purity of ordinary laboratory filter paper; the typical widths of cotton CNCs range from 5 to 20 nm and lengths from 50 to 300 nm (with an average at ∼120 nm). Similar to CNFs, CNCs are also prepared using a top-down approach. By contrast, however, CNCs are typically isolated using an acid-catalyzed hydrolysis, which selectively degrades the more reactive disordered regions of the cellulose microfibril and leaves intact the crystalline regions.^79,131−133^ The length (or DP) of CNCs is largely governed by the LODP of their source material (see section 2.2.2 for a description of LODP and Figure 2). The conventional method to isolate CNCs is to perform a hydrolysis reaction in the presence of concentrated sulfuric acid at elevated temperatures, which in addition to hydrolyzing the disordered regions introduces charged sulfate half ester groups on the CNC surface, thus imparting colloidal stability.^134−137^ Recently, many alternative isolation methods for CNCs have surfaced, based on, e.g., oxidation and esterification, but these approaches often require the additional presence of a strong mineral acid to perform the hydrolysis of disordered regions.^138^ A critical factor is that CNCs require some type of a surface charge group to come up with a stable colloidal dispersion in water. While sulfated CNCs are still overwhelmingly the most produced and studied, CNCs stabilized by phosphate^139,140^ and carboxylate groups^107,133,141,142^ have gained ground in recent years. For comprehensive accounts of CNC preparation, the interested reader is referred to recent reviews on the topic.^138,143,144^ One of the distinguishing characteristics of CNC dispersions is that they spontaneously form (lyotropic) chiral nematic liquid crystals,^145^ a feature that has spawned a sizable branch of CNC research over the past 30 years.^146−148^

The current trend in the literature is to regard BC as the third member of the nanocellulose family because it is produced by various bacterial species from low molecular weight sugars using a bottom-up approach (Figure 3c). BC is directly synthesized as a hydrated nanofiber network, i.e., a hydrogel,^149,150^ and unlike CNF and CNC preparation, it requires no isolation steps apart from washing away the bacteria and growth culture medium with mild alkali. Although the bottom-up preparation route for BC is distinctly different from the other nanocellulose types, BC is essentially a form of CNF as it consists of semicrystalline, flexible threads. In contrast to plant-based CNFs, BC nanofibers are like flat ribbons, that is, their cross-sectional dimensions are rectangular: ca. 7 nm high and 20–140 nm wide.^119^ After rinsing off the bacteria, BC is also the purest form of nanocellulose without remnants of hemicellulose or lignin and virtually without any charge. The chemical purity is among the reasons why BC has been popular, particularly in biomedical applications.^151^

Both CNCs and CNFs show a longitudinal twist with a right-handed chirality, which ultimately originates from the crystalline structure of cellulose.^152,153^ Molecular dynamics (MD) simulations have established the twist in molecular cellulose^154^ as well as in cellulose I crystal.^155,156^ Combining computational and experimental data, Conley et al. quantified a twist of 800 nm period for wood-based CNC.^154^ As the CNCs from typical sources like wood, cotton, or ramie have lengths spanning 50–300 nm, the long periodicity in the twist may be the reason as to why the twist is rarely visually evident in microscopy images of common CNCs. Despite this, the twisting of the cellulose crystal ultimately causes the formation of chiral nematic liquid crystals in CNC dispersions.

A noteworthy physical distinction in aqueous dispersions of CNFs and CNCs is that CNFs form gels at very low concentrations (<1 wt %), whereas CNCs are fluid dispersions of fairly low viscosity.^9^ CNCs do gel, but usually the gelling point is above the critical concentration for liquid crystal formation (ca. 10 wt %).^157^ These differences in water binding capacity are a partial reason for the different approaches in applications-related research concerning CNFs and CNCs. CNFs are often applied as scaffold structures in hydrogels, or as entangled networks, whereas CNCs are utilized when, for example, discrete particles are needed or sophisticated chemical modifications are deployed for self-assembly or responsive materials.^2,27^ This distinction in applications is by no means a strict one, but it has set an underlying trend for research over the past 15 years.

#### 2.3.2 Role of Water in Nanocellulose Production

**Figure 4:**
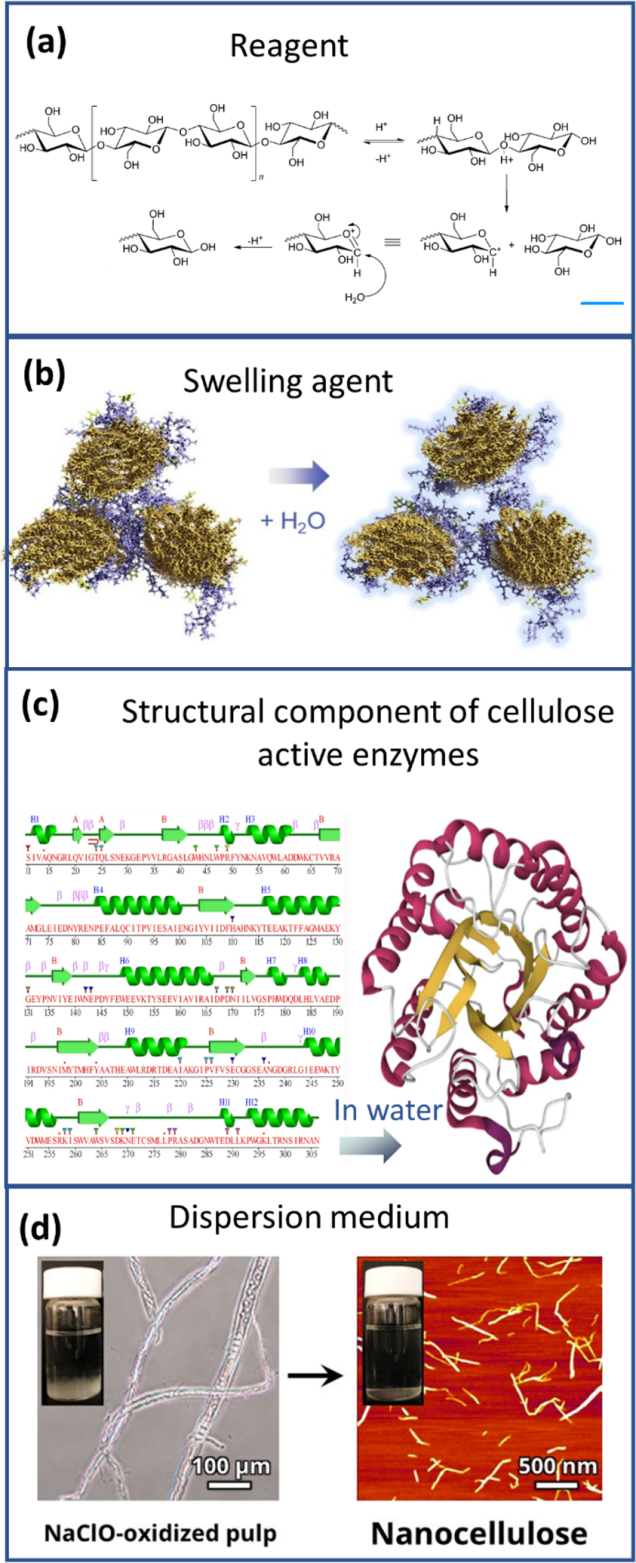
Schematic summary of the role of water in nanocellulose production. Water serves as (a) reagent^198^ and (b) swelling agent,^199^ (c) an essential medium for tertiary structures of enzymes^200^ used for nanocellulose production, and (d) medium^195^ in the hydrolysis reaction, (a) Adapted with permission from ref (198). Copyright 2011 John Wiley and Sons. (b) Adapted from ref (199) under the terms of CC-BY. Copyright 2021 Elsevier. (c) Adapted from ref (200) under the terms of CC-BY 4.0. Copyright 2021 Springer Nature. (d) Adapted from ref (195) under the terms of CC-BY. Copyright 2020 American Chemical Society.

There is no doubt that water plays an important role in the production of nanocellulose, particularly in the hydrolysis reaction utilized to isolate CNCs (discussed in detail in section 2.3.2.2).^158^ However, even when not directly involved in chemical reactions of nanocellulose production, the presence of water is pivotal. Figure 4 summarizes the role of water in nanocellulose preparation.

##### 2.3.2.1 Cellulose Hydrolysis

Hydrolysis is a chemical reaction of a substance with water, leading to the decomposition of both the substance and water.^159^ The isolation of CNCs typically occurs through a controlled hydrolysis reaction in which the β-1,4-glycosidic bonds in the disordered regions of the microfibril are cleaved by the addition of a water molecule (Figure 4a). Therefore, all CNC production routes rely on the presence of water as a reagent. Ultimately, as a result of hydrolysis of cellulose, glucose is released in a process called saccharification. However, under ambient conditions, this is a very slow reaction, and it is not applicable for practical purposes.^160^ The hydrolysis of cellulose can be expedited via different catalysts such as acids and bases, enzymes, or by using subcritical and supercritical water as a reaction medium. While the hydrolysis in subcritical and near critical conditions of water has been rarely used for the isolation of CNFs,^161,162^ it has been more actively demonstrated in the production of CNCs. For example, the use of subcritical water (120 °C and 20.3 MPa for 60 min) allows higher diffusion, activity, and ionization of water but leads to relatively low yields of CNCs (around 20%).^163,164^ Also, in a recent study, supercritical carbon dioxide and enzymes were studied to hydrolyze the disordered regions of the cellulose microfibrils to produce CNCs.^165^ As mentioned earlier, the most common method to isolate CNCs is through an acid catalyzed hydrolysis using concentrated aqueous mineral acids such as sulfuric acid, phosphoric acid, and hydrochloric acid to yield charged or uncharged CNCs (such as the case for HCl hydrolyzed CNC). Recently, Pääkkönen et al. reported the production of carboxylated CNCs via HCl gas hydrolysis, followed by a TEMPO-mediated oxidation, leading to lower acid consumption and more effortless process steps toward easier CNC purifications.^79,107^ The use of HCl gas to produce CNCs from cellulose fibers relies on the water naturally present on the cellulose surface: water is able to dissociate the HCl and carry out the acid hydrolysis.^79,107^ In addition, solid acid catalysts (such as carbon catalysts with weakly acidic groups, polymer-based acids, magnetic solid acids, and lignin-based catalysts) have been used.^166−171^ Bronsted acid active sites on the solid catalysts offer advantages such as selective cellulose hydrolysis, long catalyst lifetime, reusability, reduction of acid pollutants, and reduction of the cost of wastewater treatment. However, the contact between the active sites and cellulose remains a challenge because both reactant and catalyst are present in solid phase. The role of water as the reaction medium is crucial to improve the accessibility of catalyst to cellulose. Additionally, water can act as a catalyst for a autohydrolysis process, as hydronium ions (H_3_O^+^) formed on the surface of catalyst further promote cellulose hydrolysis.^172−174^ Despite the multiple reports on gas or solid based methods to hydrolyze cellulose into nanocellulose, these processes remain to be less common in comparison to liquid acid hydrolysis.

In acid catalyzed hydrolysis, the reaction mechanism involves the activation of the glycosidic oxygen by protonation before water addition,^175^ and the rate of hydrolysis is dependent on both the acid concentration (i.e., fraction of water) and the temperature. At very high acid concentrations and temperatures, cellulose undergoes complete degradation into singular sugars.^176^ Therefore, for the purposes of implementing acid catalyzed hydrolysis in CNC production, reaction conditions must be strictly controlled.^177^ Both the temperature and acid concentration must be sufficiently high to cleave the glycosidic bonds in the disordered regions of the microfibril in a timely manner but low enough to keep the crystalline regions intact (Figure 3b). In the conventional sulfuric acid hydrolysis to produce CNCs, the concentration is generally set to 64–65 wt %, which is fairly close to the value where total hydrolysis/dissolution of crystalline cellulose occurs (72 wt %). Here, the water content is very low because of the formation of oxonium ions, and this is integral for enabling the esterification of sulfate groups which must take place in order to ensure the necessary colloidal stability of CNCs. Furthermore, it is important to note that during the acid hydrolysis of cellulose there exists a competition between the dehydration of cellulose (i.e., cleaving of the glycosidic bonds) and the dissolution of lower DP sugars resulting from the hydrolysis.^79,178−180^

While the use of enzymes has been reported for the isolation of CNCs, they are typically implemented in the pretreatment of cellulosic substrates prior to high shear mechanical treatments.^181^ Hydrolysis of the cellulosic substrates through enzymatic hydrolysis significantly decreases the amount of energy required to mechanically isolate individual cellulose microfibrils from cellulose fibers. In nature, the degradation of cellulose is accelerated by more than 17 orders of magnitude by cellulases, which include a variety of enzymes called glycoside hydrolases (or glycosidases) that catalyze the hydrolysis of the β-1,4-glycosidic bonds of cellulose.^182^ Enzymatic hydrolysis involves complex interactions between enzyme, cellulose, and the reaction environment, and although the complete mechanism of action of the above-mentioned enzymes is still unknown^183^ it has been shown that enzyme folds and crevices are formed in water into which the substrates fit.^184^ Different cellulases will catalyze the hydrolysis at different locations along the cellulose polymer chain, and all of these enzymes act synergistically in order to fully degrade cellulose to glucose for the production of biofuels, for example.^185^ In the production of CNFs, however, the enzymatic activity of cellulases is carefully controlled to minimize undesired cellulose degradation. Furthermore, the isolation of CNFs is often aided by the introduction of enzymes such as xylanases, laccases, and lytic polysaccharide monooxygenases, which have an affinity for the glycosidic bonds in other polysaccharides (hemicelluloses in particular).^186−190^

##### 2.3.2.2 Water as a Medium in Nanocellulose Production

Cellulose and cellulosic fibers are insoluble and relatively inert in water under ambient conditions. Nonetheless, the hygroscopic nature of cellulose enables the swelling of cellulose fibers as a result of water sorption, which is key in the production of CNFs. Water-induced swelling of cellulosic fibers “opens” their structure (Figure 4b), increasing their accessibility, and in turn facilitates the penetration of chemical reagents and activity of enzymes (Figure 4c) during the pretreatment stages of CNF production. Additionally, these pretreatment methods often rely heavily on the water present to act as the reaction medium (e.g., in the case of TEMPO-mediated (Figure 4d) or enzymatic oxidation).^191^ Pretreatment methods such as swelling or partial dissolution in ionic liquids or deep eutectic solvents (DES) are also heavily dependent on water, further highlighting its importance in the many possible routes available for nanocellulose production.^192−195^ It is important to note that even in production methods where water does not play an active role in the breakdown of the cellulose structure to the nanoscale (e.g., nanocellulose production through oxidation using an electron beam), water is omnipresent in the purification, workup (e.g., alkaline treatment, sonication, and high-pressure homogenization), and often storage stages of nanocellulose production.^195−197^

### 2.4 Nanocellulose–Water Systems: Properties and Dynamics

Before introducing the interactions between water and nanocellulose specifically, it is important to highlight the distinction between the swelling of cellulosic (plant-based) fibers and nanocellulose networks in water. In nature, the geometrical constraints of the cell wall set by their hierarchical structure, especially with rigorous microfibril alignment in the secondary wall, greatly restrict the swelling capacity of fibers. As already stated, swelling in water is highly anisotropic for fibers, as the volume increase occurs solely in the lateral dimension. The isotropic nature of most nanocellulose networks, together with the significant increase in specific surface area (SSA), leads to a much higher water sorption capacity per mass of cellulose. In conjunction with this increase in SSA comes an increase in the accessibility of surface hydroxy groups which can easily interact with water. Furthermore, any charged groups introduced to the surface of the nanocellulose during isolation will also alter its ability to interact with water. Phenomenologically, the nanocellulose–water interaction involves multiple overlapping phenomena such as hydration, condensation, wetting, and diffusion, which are all mediated by various interaction forces including hydrogen bonding, electrostatic interactions, and van der Waals forces. These processes unfold along different length scales, which ultimately gives nanocellulose its extraordinary hygroscopic character.

#### 2.4.1 Water and Nanocellulose Interactions at the Molecular and Supramolecular Level

##### 2.4.1.1 Accessibility of Cellulose Chains to Water

The accessibility of cellulose chains to water is governed by the availability of hydroxy groups on the surface of a cellulose crystal. While cellulose–cellulose hydrogen bonds often take precedence over cellulose–water hydrogen bonds (hence the insolubility of cellulose in water), there is also an abundance of hydroxy groups on the crystallite surface that have the propensity to hydrogen bond with water.^201^ A common method by which to quantify the availability of hydroxy groups in cellulosic materials is to substitute the hydrogen in available hydroxy groups for deuterium through a water/deuterium oxide (H–D) solvent exchange.^202^ The availability of hydroxy groups for solvent exchange is dependent on a number of factors: (i) their position in the cellulose chain (i.e., 2-, 3-, or 6-position), (ii) whether they are within the ordered or disordered region of the cellulose microfibril, and (iii) whether they are located on the surface or embedded within the crystallite (microfibril). The hydrogen atoms in the HO(2) and HO(6) hydroxy groups can act as hydrogen-bond donors to water, but the HO(3) behaves as a hydrogen-bond acceptor from water and donor to their intrachain neighbors O(5) (see, Figure 1). The accessibility of these hydroxy groups on the surface of cellulose crystals correlates with the H–D exchange behavior. For a specific hydroxy group to be available for the H–D exchange, it must be able to donate a hydrogen atom via hydrogen bond to a water molecule.^203^ For this reason, the HO(3) does not participate in the H–D exchange.^203^ Indeed, the HO(2) and HO(6) are more prone to moisture absorption, while the HO(3) has a lower accessibility, given the fact that the intramolecular hydrogen bond with O(5) has a predominant role in stabilizing the cellulose structure.^204^ In addition to the molecular position of each hydroxy group, the crystalline structure of cellulose plays a crucial role in the cellulose–water interactions (and therefore −OH group accessibility). On the crystallite itself, water accessibility is also based on the geometrical requirements of the available hydroxy groups of the cellulose that come into contact with water molecules.^155^

The degree of crystallinity of the cellulose substrate plays a role in extreme cases: the accessibility of hydroxy groups in amorphous cellulose (as measured through deuteration using dynamic vapor sorption (DVS)) is estimated to be 63%, whereas the equivalent accessibility for microcrystalline cellulose is 51%, indicating that a higher crystallinity leads to lower −OH group accessibility.^205^ Similarly, multiple works in the literature correlate the difference in water uptake capacity of CNFs and CNCs to the difference in their degree of crystallinity and (supposedly) consequent hydroxy group accessibility.^206,207^ As mentioned earlier, CNFs are isolated cellulose microfibrils which exhibit the semicrystalline structure, whereas CNCs represent the crystalline portion of the cellulose microfibril. As such, it has been speculated that the more frequent presence of the disordered region leads to a higher water uptake capacity in CNFs than CNCs.^208,209^ Yet there is no evidence of increased swelling due to an increased number of disordered regions in microfibrils, although (as already mentioned) data published by Nishiyama et al. showed the hydrogen atoms in the hydroxy groups of the disordered domains were in fact susceptible to deuteration.^73^ It is also evident that water interacts solely with the surface of CNCs and is unable to penetrate (at least to any significant degree) into the crystal structure,^208,210^ and deuteration is indicative of the number of available surface hydroxy groups^206,211^ unless substantial temperature and pressure is applied.^212^

Instead of the disputed role of the crystallinity index, the hydroxy group availability in native cellulosic structures is rather governed by the size of the cellulose crystallite, as shown by Driemeier and Bragatto in their seminal work on microcrystalline cellulose with varying crystallite widths.^213^ Also, the amount of residual hemicellulose plays a marked role in general water uptake and must not be confused with hydroxy group accessibility in cellulose per se.^213^ Particularly with CNFs, hemicellulose is often intentionally left in the structure because it facilitates the separation of microfibrils from one another.^214^ Another factor in accessibility with water is the imperfect packing of aggregated crystallites that may allow a concentration of water molecules in voids created by the phenomenon.^213^ Therefore, when examining the differences in hydroxy group availability and subsequent water uptake capacity, it is important to also consider differences in morphology, chemical composition, and flexibility. We will discuss the difference in water uptake capacity of assemblies of different nanocelluloses in section 2.4.1.2.

The accessibility of cellulose to water may also be governed by its polymorphism. Some studies have indicated that nanocelluloses of cellulose II have higher water sorption capacity than those with a cellulose I crystal structure due to the possible changes in the crystallite dimensions and the decrease in overall crystallinity as a result of the mercerization process used to convert cellulose I to cellulose II.^215,216^ Therefore, unlike cellulose I that has small disordered regions that cannot generally be regarded as fully “amorphous”, cellulose II has been speculated to exhibit genuine crystalline–amorphous transition, akin to many synthetic polymers.^217^

Water uptake is also substantially influenced by the number of charged groups in the cellulose matrix because they directly contribute to the osmotic pressure.^218^ Intrinsically linked to the osmotic pressure, the induced electrostatic forces direct the water molecules and determine much of the texture of the nanocellulose matrix, as governed by the Derjaguin–Landau–Verwey–Overbeek (DLVO) theory^219^ The counterion on the charged group also has a pronounced impact. For example, the sulfate half ester group’s counterion on CNCs directly affects the critical coagulation concentration following the Hofmeister series (N(CH_3_)^4+^ < NH^4+^ < Cs^+^ < Rb^+^ < K^+^ < Na^+^ < Li^+^), which also correlates with the interactions they have with water molecules.^220−222^

##### 2.4.1.2 Wettability of Cellulose

The mundane definition of hydrophilic/hydrophobic character of a surface is to have the static water contact angle below or above 90°, respectively. Striving for higher thermodynamic accuracy, alternative takes on the issue have used Gibbs free energy of hydration (Δ*G*_sl_): van Oss, for example, proposed that hydrophobic compounds attract each other in water when their Δ*G*_sl_ > −113 mJ m^–2^ and repel each other when Δ*G*_sl_ < −113 mJ m^–2^.^223^ Moreover, factors such as surface roughness and morphology, porosity, and fouling all play an important role in the spreading of a liquid at the air/solid interface.^224^

In addition to, and intrinsically linked to, the hydroxy group availability, native cellulose crystals are amphiphilic (as demonstrated in the cross-sectional schemes of the microfibril in Figure 1).^225−228^ This amphiphilicity is present at molecular and supramolecular level of cellulose,^229−231^ and it is governed by the structural and conformational order in addition to the roughness, purity, and porosity of the assembled nanocellulose structures. Cellulose can also undergo conformational changes to accommodate the surrounding medium.^231,232^ The distinction between hydrophilic and hydrophobic faces in the cellulose I crystallite is straightforward (Figure 1), but their behaviors are not. For example, molecular dynamic (MD) simulations have shown that the hydrophilic 110 face, which is the most represented in the external morphology of the native fibers, possesses a water contact angle of 43°, while the other hydrophobic 100 face shows a contact angle of 95°.^233^

As a side note, the noncellulosic materials adsorbed on the surface of cellulose can change the surface characteristics. Dried CNFs have been reported to accumulate a layer of noncellulosic material on the surface, which renders them less reactive than one might expect of a material rich in hydroxy groups.^232^ As a conclusion, it is fair to define nanocellulose as a family of cellulosic materials with amphiphilic nature, whereby the free energy of hydration depends on morphological factors on all length scales.

Materials with similar surface energies are inherently compatible, suggesting that understanding the surface energies of both water and nanocelluloses can give us a rough picture of their potential interactions. Surface energy can be divided into a dispersive (i.e., hydrophobic) and a polar (i.e., hydrophilic) component, the former describing the ability of a surface to participate in long-range London type nonpolar interactions and the latter in short-range “polar” interactions. In the case of liquids interacting with surfaces of differing surface energies, one can discuss their interfacial compatibility in terms of wettability (or the ability of water to spread over a surface). A surface can be considered wettable when its θ with a liquid is between 0° and 90° and not wettable at θ above 90°.^234^ We will discuss the water sorption capacity (or wettability) of nanocellulose assemblies in sections 2.4.2.4, 4.1.1, and 4.2.2. Simply put, the wettability and the water retention of nanocellulose assemblies is highly dependent on their overall crystallinity, surface chemistry (i.e., charged, uncharged, or chemically modified), and purity of the nanocellulose along with the roughness, morphology, and porosity of its assemblies.^235^

In section 2.4.1.1, we described the ambiguous role of crystallinity in the accessibility of hydroxy groups in cellulosic materials. One can imagine that on the nanoscale the wettability of crystalline cellulose with water is also dependent on its crystal structure and the subsequent degree of amphiphilicity. However, a comparison of the surface energies of nanocelluloses extracted from several plants did not reveal a significant difference (regardless of variability in crystallinity), most likely due to the analogous surface chemistries as a result of the similarity between the biological processes of cellulose production in nature.^236^ To provide the reader with an idea of the order of magnitude of the surface energy of cellulose, a few reported values are shown in Table 1.

**Table 1:** Values for the Dispersive and Polar Surface Energies (γ) of Various Cellulosic Materials^a^

cellulosic material	γD (mN m^–1^)	γp (mN m^–1^)	γT (mN m^–1^)	method of quantification^b^	ref
hardwood α-cellulose	31.9			iGC-SEA	(237)
hardwood α-cellulose extracted with acetone	47.4			iGC-SEA	(237)
Avicel MCC	31.8	23.9	55.7	CA	(238)
Avicel MCC	51.8	0	51.8	TLC	(239)
Sigmacell 20	52.9	4.2	57.2	TLC	(239)
Whatman paper	32.1	20.2	52.3	CA	(238)
Technocel fibers	20			CA	(240)
amorphous cellulose beads	70.5			iGC-SEA	(241)
TEMPO-oxidized CNF	42–46			iGC-SEA	(242)
enzymatic CNF	51.5			iGC-SEA	(242)
cellulose II, critical CO_2_ dried	49.6	6.1	55.8	iGC-SEA	(243)
cellulose II, freeze-dried from *t*-BuOH	52.3	6.9	59.1	iGC-SEA	(243)
bacterial cellulose	47.2–58.3			iGC-SEA	(244)
amorphous cellulose	ca. 35	ca. 17	ca. 52	CA	(245)

a γ_D_ refers to the dispersive component, γ_P_ to the polar component, and the γ_T_ to the total surface energy.

b Abbreviations for method of surface energy quantification: inverse gas chromatography–surface energy analyzer (iGC-SEA); thin layer chromatography (TLC); contact angle measurements (CA).

In general, the total surface energy of cellulose is between 50 and 60 mN m^–1^ although the contribution of the dispersive and polar components can be significantly different, depending on the type of the cellulose in question.

It is evident that altering the surface chemistry of cellulose will have a significant impact on its interactions with water. The surface energies of nanocelluloses are highly tunable through a multitude of surface modification routes,^3,118,119,246−252^ which can render the material more hydrophilic or less hydrophilic. These modification pathways will be discussed in detail in section 2.1.

It should also be noted that nanocellulose structures can readily swell upon exposure to water. However, the dynamic changes caused by the wetting and consecutive drying of these structures may significantly alter the perceived surface energies of the nanocellulose surfaces.^232,245,253,254^ For example, CNFs and CNCs dried through freeze-drying tend to have a lower surface energy than those dried by air-drying, spray-drying or supercritical-drying, which is linked to their different state of aggregation.^255^

##### 2.4.1.3 Water Insolubility and the Effect of Water on Cellulose Dissolution

**Figure 5:**
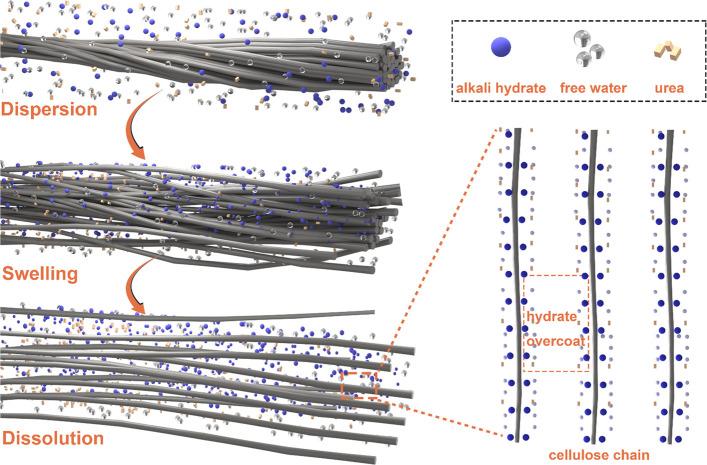
Schematic representation of dispersion, swelling, and dissolution of cellulose in water.

Cellulose is insoluble in water and other conventional solvents, inorganic and organic alike. However, several solvent systems for cellulose include water as a central component. *N*-Methylmorpholine *N*-oxide (NMMO)/water, NaOH/water, and urea/NaOH/water are among the most commonly used water-containing cellulose solvents. Unusually rapid dissolution of cellulose has been reported in urea/LiOH/water and urea/NaOH/water,^256^ albeit at temperatures below 0 °C (Figure 5). The role of water in cellulose dissolution differs a great deal depending on the solvent. In NMMO, for example, a rather precise water content of 13.7 wt % is necessary to establish the monohydrate form of NMMO, necessary for dissolving cellulose. In many recipes for dissolving cellulose, water plays a role in swelling and improving the accessibility of the chains to the solvent. Chen et al. have studied the kinetics of the dissolution and swelling of different cellulose fibers in the ionic liquid 1-ethyl-3-methylimidazolium acetate ([EMIM][OAc]) and reported that the solvent power was modified from very good (neat ionic liquid) to moderate (with 5 wt % water) and weak (15 wt % water). They showed that while the rate of fiber dissolution in neat ionic liquid depends on fiber accessibility and solvent viscosity, the water-induced decrease in solvent power dominates the general fiber behavior.^257^ Furthermore, factors such as cellulose DP, degree of crystallinity, morphology, surface chemistry, degree of substitution, and the surface tension of the solvent all play a pivotal role in the solubility of cellulose. Because dissolution is not a prominent or often-utilized phenomenon with nanocellulose (it obviously destroys the nanoscale morphology), we shall not discuss it further. Several reviews discussing the mechanism of cellulose dissolution in various solvent systems are available in the literature.^258−263^

#### 2.4.2 Behavior and Dynamics of Water within Nanocellulose Matrices

##### 2.4.2.1 Network Formation and Viscoelastic Behavior

Nanocellulose dispersions form arrested phases (i.e., a state of kinetic arrest), in the case of CNFs even at very low solids contents. Different factors such as nanocellulose aspect ratio and flexibility, surface charge density, counterion of surface charge groups, along with ionic strength of the dispersion, contribute to critical concentration at which this phenomenon occurs.^130,264−266^ The formation of an arrested phase is driven by the decrease of nanocellulose mobility either by increasing the suspension concentration or by reducing the effects of electrostatic or steric repulsion between nanocellulose fibrils/crystals.^265^ In either case, once the nanocellulose suspension reaches a critical concentration, it will go through a transition from a dispersed liquid-like state to an arrested solid-like phase. Depending on the dominant interparticle forces, two kinds of ideal arrested phases exist. In a system dominated by electrostatic Coulomb repulsion (more specifically from electrostatic double-layer repulsion due to osmotic pressure), a decrease in the interparticle distance is hindered by so-called caging effects, which leads to the formation of a colloidal glass.^265,267,268^ When van der Waals attractive forces are the dominant forces, the increase in concentration leads to the formation of a gel characterized by a percolated network often with a given fractal dimension.^265,267,268^ In nanocellulose suspensions, both repulsive and attractive forces play a role. For dilute suspensions (i.e., volume fractions below 0.05 wt %), concentrating the nanocellulose suspension will lead to the formation of a mostly reversible colloidal glass with a threshold concentration that is inversely proportional to the aspect ratio of the nanocellulose. On the other hand, increasing the ionic strength of a dilute nanocellulose suspension will result in a screening of repulsive forces between nanocellulose fibrils/crystals and the suspension will transition into an irreversible gel.^265^ At higher solids content, the dynamics involving the formation of an arrested phase may be more complicated. For example, the colloidal interactions of carboxylated CNF suspensions with concentrations ranging between 0.5 and 4.9 wt % are dominated by electrostatic Coulomb repulsion in the lower range of concentrations and by van der Waals attraction forces in higher range.^121^

The transition from a suspension to an arrested phase is quite different between CNF and CNC suspensions. Due to their rod-shaped structure and inflexibility, CNCs form a gel at relatively high concentrations. The critical gelation concentration of CNC suspensions is approximately 10 wt %. However, this is of course dependent on their aspect ratio, purity, ionic strength, and surface charge density. At critical concentrations below their gelation concentration, CNCs have a tendency to phase separate into a chiral nematic (anisotropic) phase in which the alignment of CNCs results in birefringence and an isotropic phase where the CNCs remain in suspension and repulsive electrostatic interactions dominate.^130^ In the case of CNFs, the formation of an anisotropic phase is hindered by the early onset of an arrested phase promoted by their tendency to form entanglements as a consequence of their higher aspect ratios and their flexibility. Nevertheless, CNFs can form anisotropic nematic phases under some conditions.^121,269^

##### 2.4.2.2 “Types of Water” within Nanocellulose Networks

**Figure 6:**
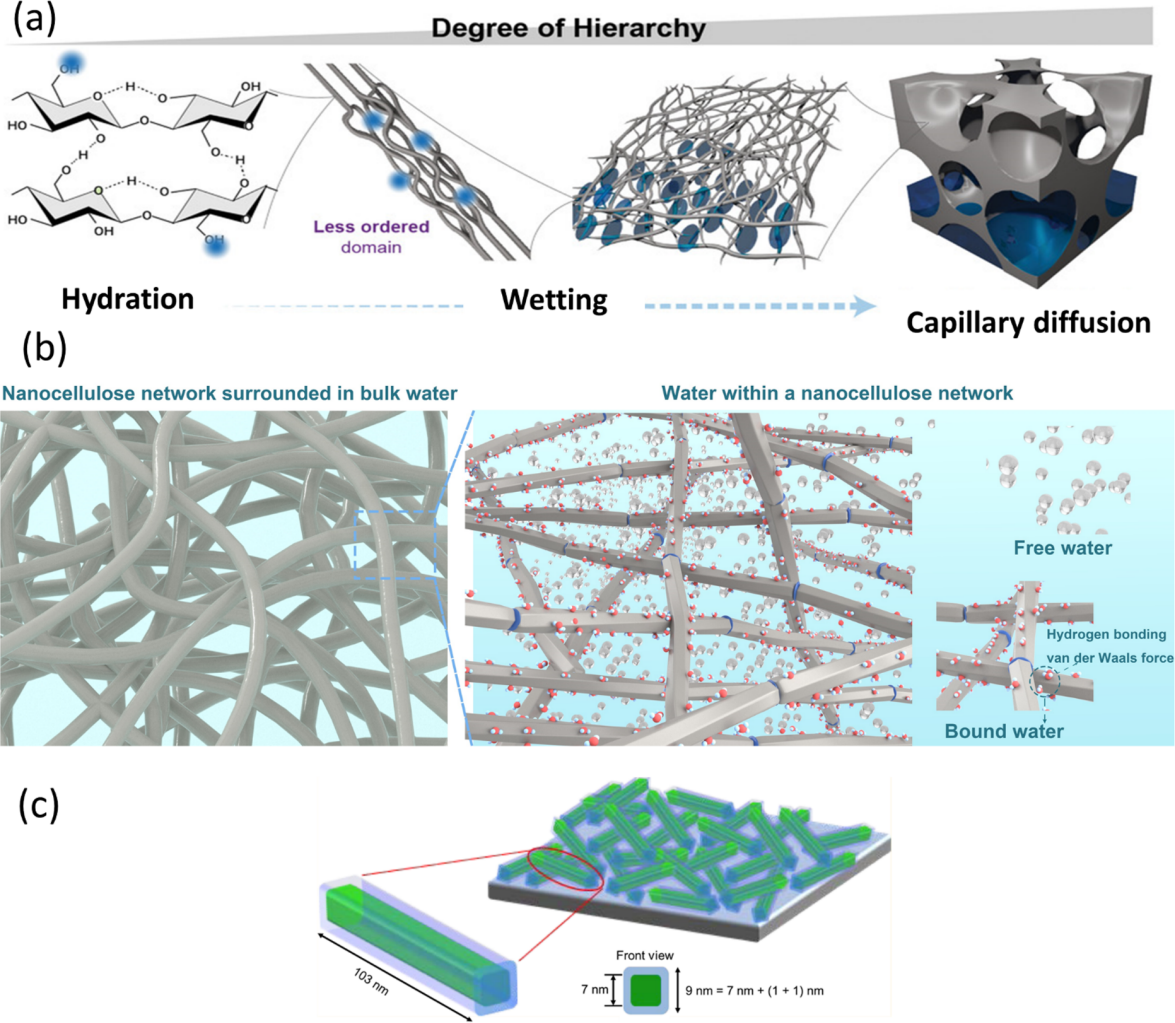
Schematic representation of wetting and hydration of nanocellulose. (a) Water and nanocellulose interactions from a supramolecular hierarchical point of view.^26^ (a) Adapted from ref (26) under the terms of CC_BY. Copyright 2020 John Wiley and Sons. (b) Wetting of cellulose nanofibers, highlighting the different states of water (bulk, free, and bound water) within nanocellulose networks. (c) Wetting of cellulose nanocrystals (in green) surrounded by adsorbed water (light blue)^271^ (c) Adapted with permission from ref (271). Copyright 2015 American Chemical Society.

Water within a nanocellulose network exists either as free water, which fills any voids due to capillary forces, and bound water, which interacts with the cellulose at specific sorption sites. At this stage, it is important to make a clear distinction in terminology: any water that is taken up by a cellulosic matrix is absorbed. However, absorbed water does not necessary interact with the cellulose through molecular interactions. When an interaction between the cellulose and water does occur, water is said to adsorb on the cellulose. Another means by which to distinguish the nature of water within a cellulose matrix, is that bound (or adsorbed) water is water present at moisture contents far below capillary condensation (i.e., the saturation point), while free (or absorbed) water is the water present in the matrix far above capillary condensation (i.e., close to saturation) (Figure 6a).^270^ With many routine experimental methods, such as dynamic vapor sorption (DVS) or water retention value (WRV), it is difficult, if not impossible, to distinguish between absorbed and adsorbed water, and hence, the predominant term for quantified water uptake is often “water sorption”.

Interestingly, the properties of water within a nanocellulose network are highly dependent on whether the water is free (absorbed) or bound (adsorbed) (Figure 6b) based on the melting and freezing behavior of water within a nanocellulose network (as measured through differential scanning calorimetry (DSC) and explained in detail in section 4.2.5), water can be categorized as (i) free water, (ii) nonfreezing bound water, or (iii) freezing bound water.^272,273^ The thermal properties of free water are the same as pure water, while bound water shows lower transition temperatures due to its strong interaction with cellulose surfaces (primarily hydroxy groups) mediated by hydrogen bonding and the consequent restructuring of local water environments and nanoconfinement.^270,274^ Bulk water surrounding nanocellulose is distinguishable from free water, as it does not cause observable swelling in the cellulose matrix as in the case of free water.^275^

It is possible to detect whether water bound to cellulose is freezing bound water (or surface bound water) or nonfreezing bound water (or confined bound water) depending on its mobility as measured through ^2^H NMR and ^1^H NMR.^270^ This classification is particularly interesting (explained in more detail in section 4.2.4), as it allows one to directly connect the properties of water with its distribution inside cellulosic matrices such as fibers and nanocellulose networks. The characteristics of these networks have a great effect on the physicochemical properties of nanocellulose derived materials and on the properties of sorbed water.^208^ Another way to distinguish different types of water is thermoporosimetry, also discussed in detail in section 4.2.

CNFs form percolated fractal networks due to the formation of arrested phases upon an increase in concentration during which the 3D network of nanofibers shrink, decreasing the packing space between CNFs and forming agglomerates.^121^ Alternatively, phenomena such as coagulation, cross-linking, or ion exchange can be used to promote the formation of CNF networks. At the fiber saturation point, all of the intra-agglomerate (CNF–CNF interface) and interagglomerate (agglomerate–agglomerate interface) pores are fully hydrated.^208^ At the interagglomerate level, the surface bound water is located at the surface of the nanofiber belonging to two different agglomerates, while at the intra-agglomerate level, the confined bound water is present at the nanofibers interface belonging to the same agglomerates.^270^ Upon increasing hydration, the surface bound water becomes gradually more and more mobile due to the high accessibility between cellulose agglomerates. In contrast, the confined bound water is only marginally influenced by the hydration level due to the lower accessibility within the agglomerates^270^ Due to these dynamics, the surface bound water also is sometimes called “movable or mobile bound water”, while the confined bound water as “fixed or immobile bound water”.

##### 2.4.2.3 Water Sorption Dynamics

The dynamics of water sorption by nanocelluloses are highly dependent on whether the nanocellulose is exposed to water vapor or liquid water as the sorption of liquid water is governed by hydrostatic pressure and capillary forces, which are not present in the sorption of water vapor. Generally speaking, the degree of water vapor sorption by nanocellulose is significantly lower than its liquid counterpart.^276−278^

Nanocelluloses can uptake a significant amount of water due to their extremely high surface area to volume ratio and their abundance of accessible hydroxy groups. Furthermore, the swelling of nanocellulose networks exposes even more surface, resulting in a higher number of hydroxy groups available for water sorption. Because they are the primary sites of interaction for water on the nanocellulose surface, rate of sorption, and desorption of water in nanocelluloses has been associated with the accessibility of hydroxy groups.^215^ The accessibility is governed by factors such as surface charge content (and charge counterion), degree of aggregation, geometric constraints, porosity, crystallinity, and the thermal history of the nanocellulose (see also section 2.4.1.1).

A number of quartz crystal microbalance with dissipation (QCM-D) and surface plasmon resonance spectroscopy (SPR) studies have been carried out on CNC^271,279^ and CNF^280−282^ model films to understand the swelling of nanocellulose networks. The kinetics of CNC film swelling as a function of solvent ionic strength and CNC surface charge was evaluated in an SPR study by Reid et al.^283,284^ Interestingly, in this work, the total water uptake capacity of the CNC films was independent of both CNC surface charge and the ionic strength of the solvent, seemingly due to the restrictions on swelling capacity resulting from the presence of van der Waals forces. However, it was clear that the rate of swelling was greatly impacted by surface charge and ionic strength. Niinivaara et al. gained quantitatively similar results in a combined QCM and ellipsometry study on CNC thin films: apparently a 1 nm layer of water surrounds the CNCs at high (>90%) relative humidity values (Figure 6c).^271^ Similarly, other works have also demonstrated that the water sorption capacity of (nano)celluloses generally increases with increasing surface charge content, in addition to changes in charge counterion, ionic strength, and pH.^134,285^

Hakalahti et al. studied the water vapor absorption mechanism and dynamics into model carboxylated CNF films.^282^ They showed that below 10% relative humidity (RH) water vapor is adsorbed mainly onto the surface of CNFs through specific interaction (e.g., hydrogen bonding), whereas at humidity values between 10% and 75%, multilayers of water molecules were built up inside the CNF network following a Flory–Huggins model. When the RH exceeded 75%, the water vapor condensed in the CNF network via cluster formation, promoting the swelling of the thin film. During sorption, the water volume fraction increased from 0.21 at 75%RH to 0.59 at 97%RH.^282^

The water vapor uptake capacity of CNFs is generally higher than CNCs due to the flexibility of the network, higher hydroxy group accessibility, and residual lignocellulosic components such as hemicellulose. At 95%RH, CNFs have at least 10% more moisture than CNCs. Because of their rigidity, CNCs do not form entangled networks such as CNFs. Comparing celluloses I and II, cellulose II has higher uptake due to the difference in crystallinity, texture, and overall morphology of the samples.^215^

##### 2.4.2.4 Mass Transport

The mass transport of liquid water in porous cellulosic materials occurs as a result of capillary flow (or wicking), which is often modeled using eq 1 as established independently by both Richard Lucas^286^ and Edward W. Washburn,^287^ which provides information on how liquids move through porous media and can be used to characterize the surface energies of powdered solids.^254,288,289^
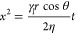
1where *x* is the distance traveled, *γ*_*l*_ is surface tension of the liquid (i.e., water), *r* is the radius of the (circular) flow channel, θ is the contact angle (assumed static) between the solid surface and the liquid (i.e., cellulose and water), η is the dynamic viscosity of the liquid (i.e., water), and *t* is time.

On the contrary, the mass transport of water vapor through porous materials proceeds via diffusion; for dense materials, a solution-diffusion mechanism is usually assumed.^290^ The diffusion driven mass transport of water vapor through porous materials is sensitive to changes in temperature and in relative humidity (and thereby partial pressure).^291^ As in the case of wetting and sorption, material properties such as thickness,^292^ crystallinity,^293^ hydrophilicity,^294^ along with porosity, and pore size and structure,^295^ all influence the rate of mass transport and are all applicable to materials such as nanocelluloses.

As most assembled nanocellulosic structures are porous, the relevant phenomena for water vapor transport through nanocellulose materials are Fickian diffusion, Knudsen diffusion, and surface diffusion. Fickian diffusion refers to classic diffusion governed by local differences in chemical potential, where the interactions between the diffusing molecule and the solid material are insignificant (i.e., when the porosity is high and pore size is large).^296^ Knudsen diffusion, on the other hand, occurs when the pore size of the solid material is comparable to or smaller than the mean free path of the diffusing molecule, leading to significant interactions between the vapor and the solid.^297^ Surface diffusion, on the other hand, occurs when molecules are mostly adsorbed on a surface, only to jump to the next adsorption site.^290^ All of these mechanisms may be found in water vapor mass transport across cellulosic materials and nanocelluloses, depending on their porosity, pore size, and surface energy.^253^ While some efforts have been made to model the observed water vapor transport of various cellulosic materials by these mechanisms, most of the literature on the uptake of water vapor by cellulose does not offer a full theoretical explanation to the experimentally observed behavior.^290,291,298^

##### 2.4.2.5 Dewatering of Nanocellulose

Recently, Sinquefield et al. published an exhaustive review on the dewatering and drying of nanocellulose.^299^ Maintaining the nanoscale properties of nanocelluloses upon dewatering/drying represents a distinct challenge, given the structural changes and irreversible aggregation which occur during the process. The most common dewatering procedures used for nanocelluloses include centrifugation,^300^ pressing,^301,302^ filtration,^303−306^ shear stress induced dewatering,^307−311^ and solvent exchange followed by solvent evaporation.^312,313^ More recently, Guccini et al. have used forward osmosis to reproducibly dewater CNFs suspensions into hydrogels with a solid content up to 12 wt %. Using this approach, they were able to retain the viscoelastic properties of the CNFs upon redispersion.^314^ After dewatering, a further drying stage is often required to bring nanocellulose to the dried state. The most common technologies used for drying are air and oven drying,^126,315−317^ freeze-drying,^318−325^ supercritical CO_2_ drying,^21,315,326,327^ and spray drying.^315,327−329^ Each of these drying techniques results in nanocellulose structures with different properties, such as thermal stability, degree of crystallinity, and char residue (upon heating or carbonization). For example, CNF films/aerogels prepared through supercritical drying have a lower stability and degree of crystallinity than those prepared by air drying or spray drying.^327^ As such, it is important to note that the drying history of nanocelluloses and nanocellulose-based materials must be carefully taken into consideration, given the influence of the drying method on their physicochemical properties.

Hornification is one of the main phenomena leading to the changes in nanocellulose materials upon drying, presumably by co-crystallization or irreversible binding of hydrophobic sites in the microfibril (see also section 2.2.2). Compared to fibers, hornification is exacerbated to a great deal in a nanocellulose networks because of the immense surface area of CNFs or CNCs. Nanocelluloses can be efficiently dried at temperatures below 100 °C under vacuum or by freeze-drying, but upon exposure to ambient conditions, atmospheric moisture will promptly be resorbed into the material.^330,331^ In fact, in ambient conditions, air-dried nanocelluloses contain between 2 and 5 wt % residual moisture.^327^ Hornification usually becomes dominant already at higher water contents, however, leading to a permanent decrease in hydroxy group accessibility. Additionally, nanocelluloses with surface moieties such as carboxyl and aldehyde groups may undergo chemical cross-linking under these conditions, further enhancing the effects of hornification.^332^ As a result, the dehydrated nanocelluloses are unable to be returned to their initial state upon rehydration.

The accessibility of the hydroxy groups in both redispersed CNCs and CNFs is reported to be reduced by ca. 84% and 82% upon drying, respectively. The structural collapse of nanocellulose during drying predominantly has negative effects on its redispersibility,^21^ chemical modification,^333^ and swelling ability. In the process of nanocellulose preparation, hornification of the source material plays a role as well. For example, pulp fibers subjected to hornification prior to TEMPO-mediated oxidation (in the isolation of CNFs) require higher energy and consume more chemicals in comparison to the never-dried pulp.^334^ In fibers, different strategies such as beating, addition of bulking agents such as sucrose and glycerol, or derivatization with spacers such as poly(ethylene glycol) (PEG) have been applied to prevent hornification.^335^ It is important to note that although the effects of hornification upon drying are usually significant, some sources of nanocellulose, such as Cladophora, show inherently lower extent of hornification.^50,336^ Due to the significant effect of hornification, it is common for nanocelluloses to be stored in their “never-dried” form in order to prevent any additional complications when utilizing these materials.^21^ Should nanocelluloses require drying, the effect of hornification (and as such the potential to redisperse the dried material in water) can be limited through the addition of salt or the exchange of counterion on surface charge groups (e.g., replace H^+^ counterion with Na^+^), which hinders the formation of new cellulose–cellulose bonding during drying.^337^

#### 2.4.3 Nanocellulose Dispersions

Nanocellulose can be colloidally stable in water but only with a sufficient amount of surface charge, as the inherent attraction between the nanoparticles affects dispersion stability. If nanocellulosic particles do not have sufficient surface charge, a material with dispersion properties comparable to cellulosic powders and macrofibers is created due to aggregation. This is similar to the conventional pulp suspensions and have been discussed in the session of aqueous fiber suspensions in a conference proceeding in 1977.^110^ The intermolecular hydrogen bonding between the surface hydroxy groups as well as van der Waals bonding between the hydrophobic sites in nanocellulose play a role in aggregation in many media, such as organic solvents, polymer matrices, and even water. Physical thinning, ultrasonic dispersion, and high-pressure homogenization can weaken these hydrogen bonds and are methods used to facilitate the (re)dispersion of nanocellulose. Introduction of surface charge or long chain molecules to increase either the electrostatic repulsion or steric hindrance between nanocellulose particles have also been used to improve dispersion.^247^ In all cases, dispersion of nanocellulose in water requires high energy input (e.g., sonication, microfluidization, high pressure homogenization) to either liberate individual nano-objects during production or redisperse prepared nanocellulose from concentrated/dried forms (Figure 5).

##### 2.4.3.1 Fundamentals of Colloidal Stability of Nanocellulose in Water

###### 2.4.3.1.1 Electrostatic Repulsion

The surface charge density and electrostatic repulsion are important factors influencing the dispersibility of nanocellulose in water, which are affected by the environmental conditions, such as pH, temperature, and salt concentration in aqueous media.^247,338^ Stable nanocellulose dispersions in water can generally be obtained when the absolute value of zeta potential is higher than 30 mV.^247,339^ Sulfate half ester,^135−137^ sulfonate,^340,341^ carboxyl,^342−346^ phosphate half ester,^139,140,347−349^ phosphonate,^350^ quaternary amine,^351−354^ and amino^355^ functionalities have been used to introduce ionic groups onto the surface and thereby improve the dispersion.^247^ The neutral sodium-form of CNCs dried by evaporation, lyophilization, or spray-drying is more easily redispersed in comparison to CNCs in acid form.^356^ Monovalent salts in the medium can improve the dispersion through hydrogen bond blocking between hydroxy groups and reducing the aggregation of CNFs and can also potentially help to regenerate hydrogen bonds between water and nanocellulose during the redispersion steps.^337^ It is important to bear in mind that the presence of divalent cationic charge in the medium, will result in cross-linking and more aggregation for the nanocelluloses with anionic charge on the surface (discussed more in section 3.3.1).

Water can play a secondary role in desulfation of sulfate half ester groups on the surface of sulfuric acid hydrolyzed CNCs that leads to destabilization and aggregation.^357,358^ Desulfation happens fairly rapidly by acid- or alkaline-catalyzed de-esterification in water. Auto-catalyzed desulfation in acid form CNC suspensions occurs slowly at ambient conditions and fast at higher temperatures and results in loss of surface charge, in addition to aggregation, desulfation has been shown to affect thermal stability, liquid crystal properties, and rheological behavior. Dried solid acid form sulfated CNCs also undergo rapid desulfation in contact with the humidity in air, in which water acts as a medium for de-esterification reaction.^357^

###### 2.4.3.1.2 Steric Stability

Neutral polymers can be immobilized on the surface of nanocellulose and expand in the medium to gain configurational entropy. As the grafted polymer layers overlap, steric or entropic repulsion between nanocellulose particles is generated.^359^ The length of grafted polymers plays an important role in the stability of the dispersion of nanocellulose in water. The common strategies for polymer induced steric repulsion are grafting PEG chains onto the cellulose surface, silylation of the surface, polymerization from the cellulose surface (e.g., surface-initiated electron transfer atom transfer radical polymerization or SI-ATRP), and adsorption of a polymeric surfactant for nonaqueous dispersions.^247,359−361^ Araki et al. covalently conjugated aminated PEG to carboxylated CNCs and the dispersion of the resulting PEG-grafted CNCs in NaCl solution remained stable. After freeze-drying, these PEG-grafted CNCs could be redispersed in water and chloroform easily.^362^ Interestingly, these PEG-grafted CNCs were also used as dispersants for fluorescent probes within cells.^363^ Similarly, poly(ethylene oxide) (PEO) grafted CNCs were dispersed stably in water for several months without precipitation.^247,364^ Some plant-based nanocelluloses disperse well in water even after oven drying due to residual pectins on the surface.^365^ The molecular weight and content of pectin control its inhibiting effect on the aggregation of CNFs during drying process. Attaching surfactants such as cetyltrimethylammonium bromide (CTAB) onto uncharged (and colloidally unstable) CNC via electrostatic adsorption has been shown to improve their dispersibility in water.^366^

##### 2.4.3.2 Manipulating Nanocellulose Dispersions in Water

###### 2.4.3.2.1 Controlling Aqueous Nanocellulose Dispersion Stability by Surface Modification and Blending with Responsive Polymers

When responsive polymers are grafted onto the surface of nanocelluloses, the dispersion stability in water can be controlled by changing environmental conditions. Thermoresposive polymers such as poly(2-hydroxyethyl methacrylate) (PHEMA) or poly(*N*-isopropylacrylamide) (PNIPAM) have been grafted onto or from nanocellulose matrices to control the water response of these systems via temperature changes,^367^ while both thermoresponsive and polyelectrolyte brushes respond to changes in ionic strength.^368^ Yi et al. synthesized the first temperature-sensitive poly[2(dimethylamino)ethyl methacrylate] (PDMAEMA) grafted CNCs by ATRP. In lower temperatures than the critical temperature, the grafted PDMAEMA chains were in extended conformation, leading to a good dispersibility in water. At higher temperatures, water dispersibility decreased.^369^ Similar thermoresponsive CNCs grafted with poly(*N*-isopropylacrylamide) (PNIPAM) were reported by Zoppe et al. Higher ionic strength, graft ratio, and degree of polymerization decreased the dispersibility in water.^370^ Azzam et al. synthesized Jeffamine polyetheramine grafted CNCs, which dispersed well in water (and other media) due to the surface carboxyl groups and polymer brushes; furthermore, the dispersion stability could be tuned by ionic strength, pH, and temperature.^247,371^ Other examples of responsive polymer-grafted CNCs prepared by radical polymerization in water (i.e., not “controlled polymerization” reactions) include pH-responsive polyvinylpyridine^372^ and dual temperature and pH responsive PDMAEMA;^373^ a full review of polymer grafted CNCs, their functionalization routes, and behavior can be found elsewhere.^374^ Larsson et al. tuned the interaction between CNF and water by adsorbing charged, heat-responsive block copolymers onto the fibers. They observed the transition of dispersed modified CNFs in water to a macroscopically aggregated state by heating, during which the adsorbed block copolymer transitioned through a critical solution temperature.^375^

Apart from surface modification, blending with different polymers can also be used to create novel forms of nanocellulose dispersions. Bai et al. have reported the formation of water-in-water liquid crystal emulsions of CNCs with permeable colloidal assemblies. They showed CNCs spontaneously self-assemble into a helical arrangement with the coexistence of nonionic, hydrophilic polyethylene glycol (PEG), and dextran. These two polymer solutions are thermodynamically immiscible. Stable water-in-water emulsions are easily prepared by mixing CNC/PEG and CNC/dextran solutions, where micrometric CNC/PEG form the dispersed droplets and CNC/dextran form the continuous phase. Over time, this emulsion breaks into an upper, droplet-lean isotropic phase and a bottom, droplet-rich cholesteric phase. Osmotic pressure gradient between PEG and dextran phases results in target transfer of CNCs across the water/water interface to reassemble into a liquid crystal-in-liquid crystal emulsion with global cholesteric organization. The authors observed that the colloidal particles in the two immiscible phases experience short-range interactions and form long-range assemblies across the interface.^376^

###### 2.4.3.2.2 Controlling Aqueous Nanocellulose Dispersion Properties by Drying

Water removal techniques have been applied as an “adjustment tool” in nanocellulose preparation with specific dispersion ability and stability in mind. As discussed earlier, redispersion in water and other solvents or polymeric blends is strongly affected by the drying method. In general, nanocellulose obtained by evaporation from aqueous suspensions is extremely difficult to redisperse, while freeze-dried sulfated CNCs and TOCNFs can be redispersed in water with a correctly chosen counterion.^21,377−379^ CNFs are less commonly dried due to their inherently entangled nature and lower charge, which leads to more difficulty in redispersing them. However, some successful drying processes and additive dispersants have been introduced for this purpose.^21^ Hu et al. reported that intrinsic adsorption of hemicellulose imparted a good redispersibility on mechanically defibrillated nanocellulose via good water accessibility of soluble hemicellulose to water comparing to that of cellulose.^380^ Foster et al. reported that the concentration of the CNC dispersion being dried influences redispersibility in water, lower concentrations lead to more redispersible dried materials. When redispersing CNCs in water from dried powders, low concentration suspensions are easiest to achieve, but if a high concentration is needed, it is best to prepare a dispersion below 2 wt % with sonication and then gradually add more CNCs with repeated sonication steps.^21^ It has also been shown that a combination of surface modification and drying techniques can be used to obtain materials with tailored dispersibility. For example, directly adding capping agents (such as specific counterions, polymers, and surfactants) prevents the agglomeration of CNFs during their dehydration (hornification) leading to CNFs with noteworthy dispersibility and colloidal stability.^381,382^

###### 2.4.3.2.3 Nanocelluloses as Dispersing Agents

The ability of nanocellulose to partition at solid/liquid, liquid/liquid, and gas/liquid interfaces has opened up new avenues to control dispersions containing nanocellulose.^383^ Nanocellulose characteristics such as size, charge, and polymorph affect their surface properties, and their ability to stabilize interfaces and act as dispersants.^247^ While nanocelluloses are not strictly “surface active”,^384^ their amphiphilicity is governed by the crystalline polymorph^385^ and any surface modification.^386^ Besides, any colloidal particle has a tendency to enrich at interfaces by default.^387^ Pickering emulsions were one of the first applications of these findings with potential in the pharmaceutics/drug delivery, personal care, food, cosmetics, and porous materials, etc.^384,388,389^ Amphiphilic particles like nanocellulose can be wetted by both water and oil and are excellent stabilizers that are essentially irreversibly adsorbed at the oil/water interface and prevent droplet coalescence compared to typical molecular surfactants. Emulsions, and particularly high internal phase emulsions, allow for formulated products with significantly less water overall but the ability to process nanocellulose under favorable and predictable aqueous conditions.^390^ CNFs have a higher aspect ratio than CNCs and often have a higher adsorption capacity and wettability by oil, which could result in more stable emulsions.^391^ However, their entangled nature often leads them to act more as a rheological modifier in the continuous water phase than as a Pickering stabilizer.^384^ Latexes are an extension of emulsion systems because they are made by emulsion or suspension polymerization, they can also be greatly improved by nanocellulose incorporation, where the role of nanocellulose varies from being a monomer–water interface stabilizer, to a water phase additive, or even a polymer particle “seed” with either active or passive participation in the chemical reactions. Importantly, nanocelluloses can be used as dispersants to improve the interfacial compatibility and prevent the agglomeration/aggregation of other noncolloidally stable particles.^247^ Amphiphilic nanocellulose is associated with 2D nanomaterials via hydrophobic interactions efficiently, whereas the hydrophilic surfaces help to disperse nanocellulose-bound 2D nanosheet in aqueous media. Surface charges stabilize the nanocellulose-bound 2D nanomaterial dispersions in water through Coulomb repulsion, where nanocellulose–water interaction is vital. Nanocellulose has been intensively used as dispersing agent for 2D nanomaterials such as graphene^392−394^ and MXenes,^395^ through intercalation. In aqueous environment exfoliation occurs via double electrostatic layers build up on 2D nanomaterials that can overcome the van der Waals interactions.^396^ Similarly, other nanoparticles, such as metal oxides,^349,397,398^ quantum dots,^399^ metal organic frameworks (MOFs),^400^ polymer nanofibers,^398^ and salt nanoparticles^401,402^ have also been stabilized by nanocellulose to disperse them in water. Lastly, the unique templating ability of nanocellulose can be used to mediate the nucleation and growth of metal nanoparticles, inhibiting nanoparticle aggregation, improving their dispersion uniformity and stability, and in many cases, enhancing their catalytic or electrochemical function, as reviewed previously.^403,404^

Interestingly, Gonzales et al. rationalized that if a surfactant with high HLB (hydrophilic–lipophilic balance) is able to stabilize oil in water emulsions, then the same type of material could hinder the formation of water in oil emulsions. They used nanocellulose as an inhibitor (demulsifier) of water in crude oil emulsion formation, which is a challenge during crude oil extraction and processing.^405^

## 3 Role of Water in Nanocellulose Modification and Applications: A Double-Edged Sword

### 3.1 Pathways to Tune Nanocellulose–Water Interactions

As discussed earlier a few times, cellulose is inherently amphiphilic,^229−231^ and altering its surface energy is possible through a wide range of surface modification methods. Due to the natural affinity between cellulose and water, it is clear that water plays an important role in the modification of nanocellulosic surfaces. Although most covalent routes are based on organic reactions where water is usually detrimental, several synthetic approaches for nanocellulose modification can accommodate having water as a solvent.^74,251,386,406−422^ One of the main motivations to surface modify nanocellulose is to improve its compatibility with other materials. For example, the dissociation of charged sulfate half ester groups in water ensures the colloidal stability of CNCs, but if one wants to handle them in nonpolar solvents (which can then be used as further modification medium), they tend to aggregate. Similarly, while as-prepared nanocelluloses are inherently incompatible with many petroleum-based polymers, hydrophobized nanocelluloses may be more easily dispersed throughout a composite matrix. Additionally, the use and functionality of assembled nanocellulose structures may require a specific surface energy (e.g., hydrophobicity in nanocellulose aerogels designed for separating oil from water). In this chapter, we focus on surface modification methods which tune the amphiphilic nature of nanocelluloses, and we further go on to highlight the role of water in these modification procedures.

We re-emphasize that the distinction between hydrophilic/hydrophobic is oversimplified in the literature, particularly when defined by static contact angle measurements. Still, it is a definition that has stuck and is widely recognized and, as such, we feel that adhering to these terms has significance in the scientific community.

When considering surface modification of nanocellulose, it is not only the nature of the substituents being grafted but also the degree of substitution that influences the hydrophilicity/hydrophobicity. Furthermore, contact angle measurements are highly sensitive to surface roughness, which can be altered after surface modification such that interpretation of results is not always straightforward. In this context, Cunha et al. showed that for the esterification of nanocellulose with trifluoroacetic anhydride even a modest degree of substitution of 0.04 had a strong effect on the hydrophilicity of the surface, resulting in a static water contact angle of 126°.^423^ This demonstrates how small changes to the cellulosic surface may have large impacts on the wetting characteristics due to both chemistry and topography.

#### 3.1.1 Decreasing Nanocellulose Surface Hydrophilicity

In this section, we focus on surface modification approaches that aim to decrease the hydrophilicity of nanocelluloses and the role of water in these reactions. This often-termed “hydrophobization” of nanocellulose leads to the disruption of the solvation of surface structures by water by capping the available hydroxy groups through the attachment of hydrophobic moieties,^424−427^ grafted polymers,^428−436^ or even nanoparticles.^437−443^ Generally, the reasons to hydrophobize the surface of nanocellulose include increasing the hygromechanical stability (keeping good mechanical properties in the wet state), improving the compatibility with hydrophobic polymers or solvents or reducing the effects of hornification upon drying.^12,444−446^ The palette of the available modification pathways is very diverse, and a full review of these reactions is out of the scope of this paper. The reader is encouraged to follow the details of modification reactions in the previous publications on this topic.^1, 3, 118, 119, 128, 246, 247,249, 251, 252, 384, 406, 408, 415, 447−486^ However, a short overview of the most important routes to nanocellulose surface hydrophobization is covered here and summarized in Table 2.

**Table 2:** A List of Covalent and Noncovalent Methods for Decreasing Hydrophilicity of Nanocellulose

altering the hydrophilicity of nanocellulose surfaces					
surface modification	reagents used	required conditions	role/consequence of water	potential target application	ref
Covalent Hydrophobization					
esterification	carbonyl chlorides, acid anhydrides	anhydrous	undergoes side reactions	sensors, mechanical reinforcement, biomedical materials, (super)hydrophobic interfacial materials	(487−493)
	darboxylic acids, active esters (potentially activating agents for in situ generation)	anhydrous/aqueous	reaction byproduct that decreases yields		(490)

darbamate formation	isocyanate	anhydrous	causes side reactions	polyurethane composites	(494)

etherification	alkyl chlorides	anhydrous	may cause phase separation, side reactions depending on substrates and reaction conditions	introduction of functional groups, e.g., carboxymethylation	(495−501)
	epoxides	aqueous/anhydrous	causes side reactions	introduction of functional groups, e.g., quaternization	(495−500)
chlorination (potential subsequent etherification)	thionyl chloride	anhydrous	causes side reactions	further modification	

oxidation (potential subsequent amination)	periodate	aqueous	solvent	further modification	(503)
amidation	EDC/NHS or comparable coupling agents, primary or secondary amines	aqueous	solvent	selective modification, biomolecule immobilization	(504−507)
amination	aldehyde/keto-groups on cellulose, amines, reducing agent	aqueous/anhydrous	solvent	protein immobilization	(503,508)

silylation	chlorosilanes	anhydrous	causes side reactions	stable dispersion in organic media	(509−511)
	alkoxysilanes	anhydrous/aqueous	solvent	porous hydrophobic adsorbent materials	(512,513)

polymer grafting (ATRP)	ATRP-agent, vinyl monomers	anhydrous/aqueous	solvent	tailor-made reinforced hydrogels	(514,515)
polymer grafting (ROP)	epoxides/cyclic lactones	anhydrous	chain transfer agent giving rise to bulk ROP	covalent composite materials	(416)
Noncovalent Hydrophobization					
polymer adsorption	hydrogen bond acceptors, countercharge carrying polymers, hemicelluloses	anhydrous/aqueous	dispersion medium, antisolvent	thermoplastic polymer composite materials	(516, 517, 518, 424, 519−528)
counterion exchange	surfactants, quaternary ammonium ions,	aqueous	dispersion medium, antisolvent	cellulose dispersion in nonpolar media	(424, 522, 529−534)

##### 3.1.1.1 Covalent Nanocellulose Hydrophobization

The formation of esters^487−490^ and carbamates on the surface of nanocellulose usually requires anhydrous environments, which is facilitated by drying techniques or the use of nonaqueous reaction media, such as gas phase esterification.^491−493^ The esterification of cellulose hydroxy groups with carboxylic acids, anhydrides, and acid derivates is arguably the most versatile tool for surface modification due to the variety of reaction pathways, including simple Fischer esterification, acid chloride or anhydride alcoholysis, and transesterification, which can be conducted in a liquid or gas phase, depending on the moiety to be attached.^490^ Polymer grafting through ring-opening polymerization or surface initiation for (controlled or not) radical polymerization on the surface via (trans)esterification have been also widely reported in the literature.^416,515^ Similarly, the grafting of isocyanates onto cellulose surfaces requires an anhydrous environment to avoid side reactions of the reactive electrophiles. The high reaction speed and yield of the addition reaction, the availability of isocyanates, and easy access to polyurethane grafting techniques add to the popularity of this approach.^494^ Etherification via thionyl chloride is also performed in nonaqueous environments, substituting the cellulose hydroxy groups with chloride.^502^ This activation facilitates subsequent substitutions, as the halogen is a better leaving group compared to hydroxy moieties. Eyley and Thielemans employed a chlorination approach on CNFs, followed by substitution of the chloride group with azide to obtain clickable cellulose moieties.^501^

Some reactions for hydrophobization, such as silanization, can be carried out in both aqueous and anhydrous environments.^513,535−541^ Reactive chlorosilanes undergo hydrolysis in aqueous environments, therefore, they are used to silanize nanocellulose in anhydrous environments. These reagents are chosen for their high reactivity and therefore short reaction times when in contact with cellulose. However, the need for tedious solvent exchange procedures and the release of hydrogen chloride are drawbacks in the synthesis of silylated components by chlorosilanes.^509−511^ Alternatively, corresponding alkoxysilanes can be used to obtain the same desired compounds. These reagents are not as sensitive to water but are less reactive, and therefore, reaction times are increased.^512,513^ While the deprotonated cellulose hydroxy groups act as nucleophiles in etherification reactions (explained in the next paragraph), auxiliaries can be employed to transform the cellulose component into an electrophile. Historically, this involved the use of thionyl chloride as explained previously.^501^ Alternatively, in etherification via employing tosyl chloride reactions, the hydroxy groups can be converted into tosylates, which in turn are better leaving groups than chlorides. While the tosylation of cellulose is usually carried out in a nonaqueous environment such as DMA/LiCl,^542^ the reaction can also be conducted in water.^543^ Guo et al. used tosylation on nanocellulose to prepare clickable cellulose azides.^544^

**Figure 7:**
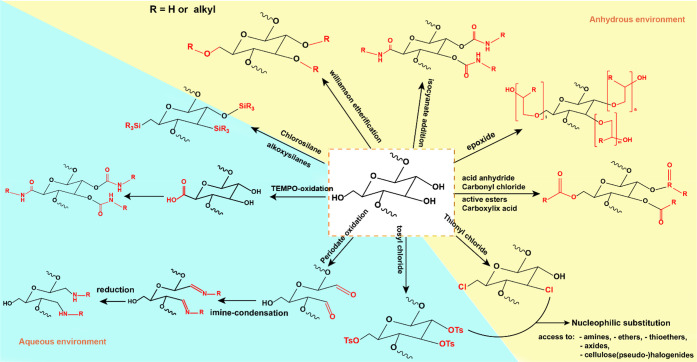
Most common initial reactions of cellulose, as the foundation for more elaborate modifications and their compatibility (blue)/sensitivity (yellow) to water.

Some other cellulose modification reactions are safe to be carried out in water. Etherification of surface hydroxy groups can easily be achieved by reacting cellulose with glycidylethers or chloro-compounds, most prominently chloroacetic acid, in a basic aqueous solution.^495−500^ Basic media are needed to activate the surface hydroxy groups via deprotonation. The formed alkoxide species are sufficiently nucleophilic to add to epoxide moieties or to substitute halogens. The oxidation of nanocellulosic surfaces in aqueous media to aldehydes and acids (which initially increases the hydrophilicity of the surface and on its own will be discussed in section 3.1.2) followed by further functionalization is an important pathway to preparation of hydrophobized nanocellulose surfaces. As mentioned in the previous section, oxidation is also considered to be a very common step in the isolation of different types of nanocellulose, often ending up in carboxylate moieties on the surface.^342,545−547^ Nanocellulose surfaces containing carbonyl groups (such as in aldehydes and ketones) can be used as substrates for Schiff’s base reactions, including the attachment of amines,^503,508^ while the carboxylic groups can further be modified through activated peptide coupling.^504−506^ A variety of coupling agents have been developed over the past decades to facilitate peptide synthesis and can be used for this purpose in aqueous environments.^507^ An overview of covalent routes to nanocellulose modification are depicted in Figure 7.

##### 3.1.1.2 Noncovalent Hydrophobization Routes

In addition to chemical pathways to decrease the hydrophilicity of the surface, physical interactions are other ways to hydrophobize nanocellulose surface in aqueous and nonaqueous systems. Physical surface hydrophobization (essentially adsorption or counterion exchange) can be achieved in a facile and cost-effective process without the need for complex or multiple chemical reactions. These physical routes usually preserve the nanocellulose crystallinity and morphology better, but they are weaker and more likely to be reversible,^386^ compared to chemical modification via covalent bonds. Often, nanocellulose surface modifications via physical interactions are based on electrostatic interactions or polymer adsorption.^548−554^

The simplest practical way to approach adsorption as a modification system is to use water-soluble polymers.^555^ For example, carboxylated CNFs and amphiphilic diblock copolymer poly(methyl methacrylate-*b*-acrylic acid) form a stable complex. Due to the hydrophobic poly(methyl methacrylate) block, the surface modified CNFs exhibit good dispersibility in different organic solvents including DMF, DMSO, ethanol, and methanol.^516^

In addition to hydrophilic interactions, the charges on the surface of nanocelluloses can be employed to physically modify the surface. For example, the negative surface charge on CNCs prepared by sulfuric acid hydrolysis (i.e., sulfate half esters) and CNFs prepared via TEMPO-mediated oxidation (i.e., carboxyl groups) can interact with polyelectrolytes.^103,517,518^ Hydrophilic CNFs can be hydrophobized through the adsorption of octadecyl amine resulting in their dispersibility in solvents such as toluene, tetrahydrofuran, and isopropyl alcohol. However, this surface modification is unstable and will desorb over time.^424^ Esker et al. utilized the adsorption of hydrophobic polyelectrolytes (i.e., cationically derivatized hydrophobized dextran polyelectrolytes) to decrease the surface water on sulfated CNCs and expedite their dewatering kinetics. Using electrostatic interactions, block copolymers based on quaternized poly(2-(dimethylamino)ethyl methacrylate) and polycaprolactone can also be attached to anionic nanocellulose surfaces, resulting in a decrease in the surface energy of the cellulose surface. The benefit of modification pathways such as this is that they can be carried out in an aqueous environment.^424,519−522^ Yahia et al. used poly 1-[4-(bromomethyl)phenyl]-1,2,4-triazole to modify the surface of CNCs through electrostatic adsorption, which significantly altered their electrophoretic mobility and enabled their dispersion in acetone and acetonitrile.^556^ They even took the modification a step further by substituting the bromine with bis(trifluoromethane sulfonyl)imide, which resulted in nanocellulose complexes able to disperse in tetrahydrofuran and ethyl acetate.^386^

A simple counterion exchange of the charged groups on nanocellulose surfaces can also be used to tune surface energy in order to decrease self-aggregation and improve compatibility with nonpolar solvents and hydrophobic polymers. In particular, substituting the counterion of CNCs with an imidazolium and phosphonium cation improves CNC compatibility with polymers such as epoxy and polystyrene and decreases their ability to interact with water; phosphonium ion exchanged CNCs absorbed 30% less water than the sulfate CNCs in their sodium form.^534^ In the case of CNFs, a mixed system of sodium and tetraethylammonium counterions has been used to control CNF film hydrophilicity. The oxygen and water vapor permeabilities of said films increased with an increase in the molar ratio of bulky tetraethylammonium counterions.^529^

Physical adsorption of surfactants onto nanocellulose is another strategy to reduce the capacity for intermolecular hydrogen bonding. Common surfactants such as cetyltrimethylammonium bromide (CTAB), dimethyldidodecylammonium bromide (DDAB), and dimethyldihexadecylammonium have been demonstrated to stabilize nanocellulose dispersions in THF.^522^ Additionally, amphiphilic polymers, which contain both polar and nonpolar moieties, can interact with nanocellulose surface such that their polar end interacts with the nanocellulose and the nonpolar remains in solution.^523^ Additionally, nonionic surfactants, such as sorbitan monostearate, have been used to modify CNC surfaces to improve their dispersibility in organic solvents and prevent self-aggregation.^530^ The acid phosphate ester of ethoxylated nonylphenol surfactant (Beycostat A B09) was also reported to improve the dispersibility of CNCs in chloroform.^531^ Recently, Kontturi et al. presented a nanocellulose surface hydrophobization technique through the adsorption of hydrophobic polymers (e.g., polystyrene and poly(trifluoroethylene)) from aprotic solvents, resulting in nanopapers with water vapor uptake ability yet a strong repellency for liquid water, but this method is restricted to macroscopic substrates such as nanopaper and does not work for individual nanoparticles like CNFs and CNCs.^524^

Carbohydrates and soluble cellulosic materials have also been used to tune the interactions of nanocellulose surfaces with water. Larsson et al., for example, altered the behavior of composite films of both CNFs and CNCs with water through the adsorption of water-soluble hydroxypropylmethylcellulose (HPMC),^525^ while numerous works have shown altered CNC–water interactions as a result of insoluble oligosaccharides precipitated onto the surface of nanocellulose.^526−528^ Adsorption of water-soluble polysaccharides is indeed an often applied approach to alter the surface of (nano)cellulose, but in most cases it does not result in more hydrophobic surfaces. In fact, in many cases the surface becomes more hydrophilic through the attachment of polysaccharides, specifically methylcellulose, hydroxyethylcellulose, guars, carboxymethylcellulose, and hemicelluloses have been demonstrated.

#### 3.1.2 Increasing Nanocellulose Surface Hydrophilicity

In this section, we focus on surface modification pathways to increase the hydrophilicity of nanocelluloses, and we highlight the role of water in these procedures. Despite the hygroscopic nature of cellulose, its amphiphilicity can hinder the use of nanocelluloses in applications where high levels of hydrophilicity are required, such as in the case of superabsorbents, for example. As with surface hydrophobozation methods, both covalent and noncovalent pathways can be implemented. Table 3 is a summary of the most important nanocellulose surface hydrophilization modification routes and the role of water in these procedures.

**Table 3:** A List of Covalent and Noncovalent Methods for Increasing Hydrophilicity of Nanocellulose

altering the hydrophilicity of nanocellulose surfaces					
surface modification	reagents used	required conditions	role/consequence of water	potential target application	ref
Covalent Hydrophilization					
esterification	mineral acids	aqueous	solvent	dispersion/gelation control	(136, 137, 347)
oxidation	TEMPO, hypochlorite	aqueous	solvent	dispersion/gelation control	(411)

polymer grafting	acrylates (polyelectrolytes)	aqueous	solvent for monomer, dispersant for cellulose, grafted polymer	covalent composite materials	(370, 416, 557)
	PNIPAM (thermoresponsive)	aqueous	solvent for monomer, dispersant for cellulose, grafted polymer	covalent thermoresponsive composite materials	(367)
Noncovalent Hydrophilization					
counterion exchange	monovalent counterions	aqueous	dispersion medium	“salting-in” effects for improved colloidal stability	(220)
chaotropic co-ions	chaotropes according to the hofmeister series with equal charge to the cellulose	aqueous	dispersion medium	defined swelling of cellulose hydrogels	(558)
dilution		any solvent, not fully anhydrous	reduction of hornification by dilution		(332)

##### 3.1.2.1 Covalent Nanocellulose Hydrophilization

The hydrophilicity of nanocelluloses can also be improved by grafting hydrophilic polymers (e.g., acrylate based superabsorbent polymers),^370,416,557^ although grafting hydrophilic polymers is less explored than grafting hydrophobic polymers. Kaldeus et al. reported an all-water-based procedure for “controlled” grafting of hydrophilic polymers from CNFs; polymers and copolymers of PEG, methyl ether methacrylate, and poly(methyl methacrylate) were synthesized by ATRP from the CNF surface in water. This was made possible through an amphiphilic macroinitiator that was electrostatically immobilized on the CNF surface, facilitating both hydrophobic and hydrophilic monomer polymerization in water.^514^ Here, the precise mapping of water interactions would be an important fundamental undertaking as the grafted nanocelluloses possess a corona that can hold rather vast amounts of bound water compared to pristine (or charged) CNFs and CNCs that have bound water primarily on their surfaces.

##### 3.1.2.2 Noncovalent Nanocellulose Hydrophilization Routes

The most popular approach to increase the hydrophilic character of nanocellulose is to impart additional charge onto the surface.^559^ Nearly always, nanocellulose bears some degree of surface charge directly after production with a certain degree of tailoribility based on reaction conditions. With TEMPO-oxidation^79,132,342,560^ and periodate oxidation,^503^ the surface charge can be varied by up to an order of magnitude, whereas sulfuric acid hydrolysis “harshness” can only affect the degree of sulfation within a relatively small window.^561^ In addition to mere hydrophilic character, subtle changes in charge caused by varying degrees of TEMPO-oxidation have been applied to control wetting and antifouling properties.^562^ However, it is important to bear in mind that surface charge makes the behavior of nanocellulose in water susceptible to ionic strength. The easiest way to suppress electrostatic interactions is to increase the ionic strength of a nanocellulose suspension and this will lead to nanoparticles aggregation and loss of colloidal stability according to the DLVO theory.^220^ Furthermore, the counterion on the nanocellulose surface charge group affects the water interactions and leads to different coagulation behavior in water and other solvents.^220^ Zhang et al. illustrated the effect of cosolvent choice (i.e., sodium salts of various anions) on the swelling behavior and thus the mechanical properties of polymeric hydrogels.^558^ While these principles also apply to cellulose, the overall ionic strength may lead to a screening of the surface charges and in turn to different levels of interaction between water and (aggregated) nanocelluloses.^563^

### 3.2 Role of Water in Controlling Surface Modification Reactions

Unfortunately, the presence of water can be detrimental in some nanocellulose surface modification mechanisms, due to its incompatibility with the required chemical reagent or reaction. In these cases, it is crucially important to remove any residual water prior to initiating the modification of nanocellulose surfaces. An anhydrous chemical environment can be achieved through techniques such as solvent exchange, the subsequent use of molecular sieves, or drying by evaporation and diffusion. However, each of these techniques has its own inherent drawbacks. Drying solvents is tedious and requires working in conditions where contact with air (and ambient moisture) is strictly prevented by working with a vacuum line for example. Molecular sieves, on the other hand, can introduce impurities into the reaction medium, which can also inhibit the surface modification reactions of nanocellulose. There are also other methods for water removal prior to chemical modification, such as adding alkaline materials or exploiting ionic liquids while water is being removed.^312^ While solvent exchange is a fast and relatively easy method to remove water from nanocelluloses, residual water often remains in the system afterward. For surface modification techniques requiring anhydrous conditions, one would typically use fully dried nanocellulosic substrates, but it is important to recognize the effect of hornification (irreversible aggregation) on the efficiency of these chemical modifications.^332,564,565^ Indeed, many accounts utilize solvent exchange or extensive drying step to introduce nanocellulose into anhydrous media, perform reactions on the nanocellulose, and report a certain degree of substitution. However, they do not necessarily disclose the morphology of nanocellulose after the reactions. It may be that in some cases large swathes of, for example, CNC particles remain completely unmodified in larger aggregates that have accommodated all the substitutes on their surfaces. All in all, it appears that aqueous medium is the safest place to work on comprehensive modification of nanocellulose in a way that all nanoparticles are evenly modified.

#### 3.2.1 Controlling Dispersion in Modification Reactions by Tuning the Water Content

Solvent-exchange often leaves behind a fraction of residual water in nanocellulose systems,^510^ which might be detrimental to some organic reactions, but minute amounts of water may under specific conditions play a positive role, for example, in CNC dispersion in organic media.^247^ Viet et al. found that a small amount of water is critical to colloidally stabilize sulfated CNCs in polar aprotic solvents such as DMSO and DMF. Freeze-dried CNCs with residual water contents of 6–8% were able to form stable and well dispersed suspensions. Yet further removal of moisture using molecular sieves resulted in the aggregation of the CNCs. Interestingly, when 0.1% w/v of water was reintroduced into mixture, a stable suspension reformed. The authors concluded that a small amount of water (<0.2% w/v) is indeed necessary for a colloidally stable dispersion.^566^ Belgacem et al. investigated the effect of residual water on the particle size of the CNC aggregates by dispersing CNCs in water and subsequently carrying out a solvent exchange with ethanol and acetone. When measured through dynamic light scattering, the apparent size of the solvent exchanged CNCs was ca. 300 nm, whereas dried CNCs redispersed directly into acetone had an apparent size of ca. 635 nm. They concluded that the interactions of residual water with CNCs were not totally lost during solvent exchange, leading to a more stable suspension in acetone.^567^ Chang et al. systematically investigated the influence of water on the redispersion of CNCs in DMF. CNCs with a residual moisture content of ca. 4% have the same hydrodynamic radius as the theoretical radius of monodispersed CNCs, indicating that the CNCs were completely dispersed in the DMF.^568^

#### 3.2.2 Controlling Modification Outcomes by Tuning Water Content

When macroscopic nanocellulose substrates, such as films or nanopapers, are subjected to modification, the role of water is ambiguous. The high swelling capacity of CNF networks in water means that under aqueous conditions, the whole network, including its interior, will be modified. The water-immersed modification can also impair the bonding in the network if the fiber–fiber bonds are cleaved and modified. By contrast, when utilizing nonswelling hydrophobic solvents, only the surface of the network will be modified, and the network bonding remains intact. In this realm, Kontturi et al. used aprotic solvents instead of water as medium to adsorb hydrophobic polymers on the surface of nanopapers in the study already quoted in relation to the adsorption-based methods in section 3.1.1.2. Due to the limitations to the ability of these solvents to swell the nanofibers, these nonporous nanopapers were hydrophobized only on their surface. This resulted in CNF nanopapers with tunable hydrophobicity on the surface, while their water vapor absorption capacity was demonstrated to be similar to the pristine, untreated nanopapers. The strong mechanical properties, based partially on the hydrogen bonding between the CNFs have been retained, while the vapor transmission of the surface-hydrophobized nanopapers may be useful in applications such as textiles or building insulations.^524^

Sometimes water is not a good solvent for one of the reagents in a cellulose modification reaction, and in these cases, although water does not interfere with the reaction per se, the concentration of the reagent in water is too low, and this prevents the reaction to proceed to completion. Odabas et al. cationized bleached kraft pulp in systems with different water-miscible organic solvents. Replacing 90% of the water with 2-propanol and particularly with tetrahydrofuran yielded higher degrees of substitution and increased reaction efficiency. The degree of substitution depends on the concentration of the cationization reagent in the reaction medium. Replacing most of water with tetrahydrofuran resulted in a higher concentration while maintaining supramolecular properties such as crystallinity and polymer chain integrity.^569^ Although this particular study has been done on pulp fibers, the concept is likely applicable to all cellulosic materials, including different nanocellulose types.

When working with nanocellulose suspensions in water (or other solvents), there is a strong effect of concentration on viscosity and potential flow alignment.^570−573^ Unless a controlled environment is maintained around the sample, water evaporation can occur during the course of the modification reactions and significantly change the concentrations, viscosity, and alignment due to flow. Water evaporation can (and should) be reduced by implementing a closed or reflux system during surface modification reactions.

### 3.3 Nanocellulose–Water Interactions in Materials Applications

**Figure 8:**
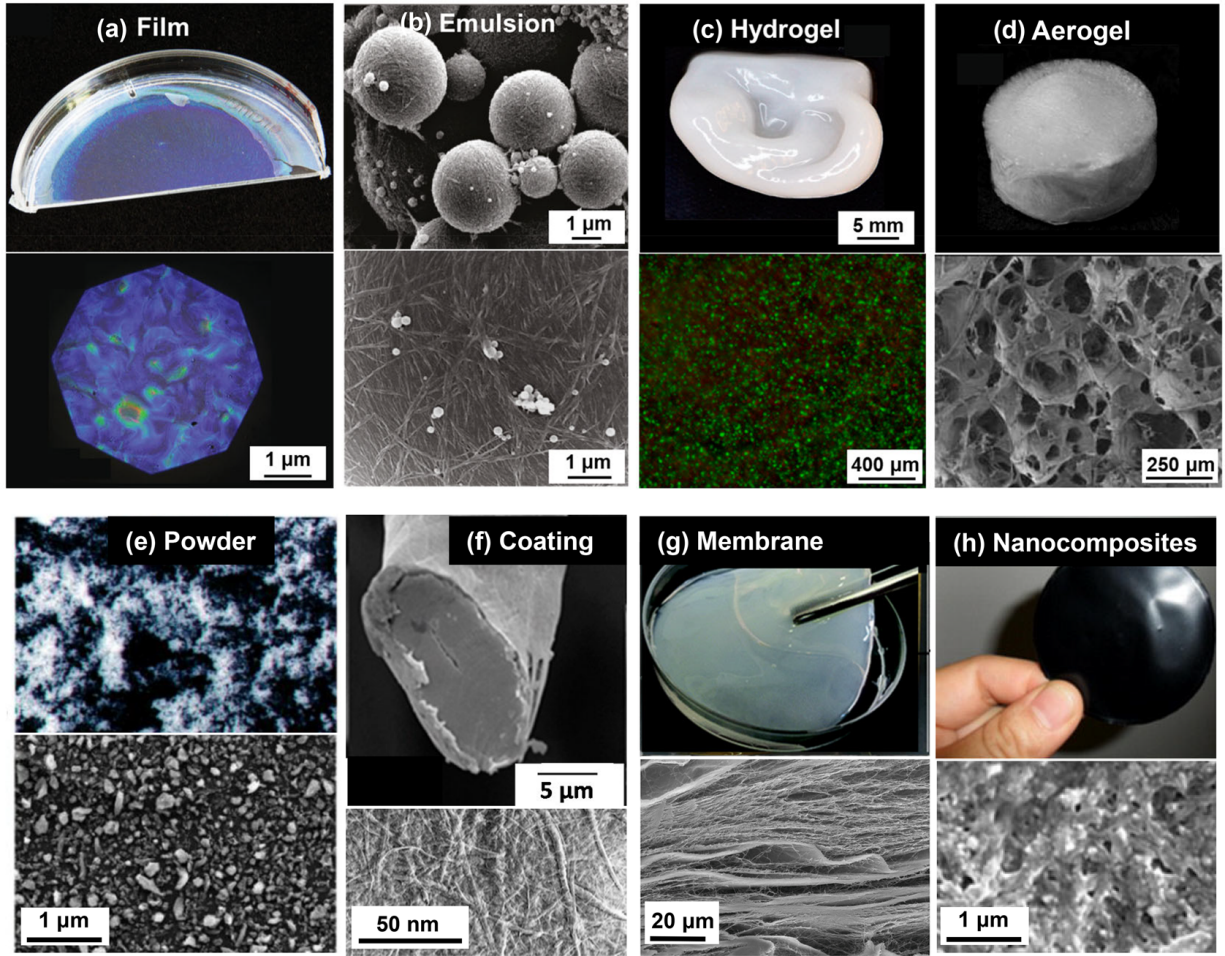
Different morphologies and systems of nanocellulose materials: (a) film,^574^ (b) emulsion,^388^ (c) hydrogel,^575^ (d) aerogel,^319^ (e) spray-dried powder,^299^ (f) coating,^576^ (g) membrane,^577^ (h) nanocomposite.^578^ (a) Adapted from ref (574) under the terms of CC-BY. Copyright 2014 John Wiley and Sons. (b) Adapted with permission from ref (388). Copyright 2011 American Chemical Society. (c) Adapted with permission from ref (575). Copyright 2015 American Chemical Society. (d) Adapted with permission from ref (319). Copyright 2005 The Royal Society of Chemistry. (e) Adapted with permission from ref (299). Copyright 2020 American Chemical Society. (f) Adapted from ref (576) under the terms of CC-BY. Copyright 2020 Multidisciplinary Digital Publishing Institute. (g) Adapted with permission from ref (579). Copyright 2018 John Wiley and Sons. (h) Adapted with permission from ref (578). Copyright 2014 Elsevier.

The following subchapters highlight the connection between water interactions and nanocellulose material applications, providing an overview of where water can be a benefit or detriment. Moreover, we discuss different synthetic and technical approaches toward tailoring the water response in nanocellulose-based materials for certain applications. Depending on the production and processing methods, different morphologies and hybrid systems containing nanocellulose can be obtained, e.g., colloids and emulsions, hydrogels, films and membranes, aerogels and foams, and nanofillers, which offer a range of properties useful across various fields (Figure 8).

The overview on nanocellulose applications is not exhaustive. Rather, we aim at exploring how water interactions are influencing the functionality of the materials described by focusing on relevant examples on a certain class of applications where nanocellulose has been used as an integral component. For the readers interested in exhaustive treatises, substantial review articles have been published, and we refer to those reviews with each relevant topic throughout this chapter. Table 4 summarizes the water related applications of nanocellulose in different forms (dispersion, hydrogel, film, aerogel, and powder) and important properties of each form for the mentioned application.

**Table 4:** Applications and Properties of Various Nanocellulose-Derived Materials Regarding Water^a^

application	dispersion	hydrogel	film	aerogel	powder
drug delivery	carriers (water sorption, swelling, biocompatibility)				emulsifiers in formulations (amphiphilicity)
tissue engineering		scaffolds (water sorption, swelling, biocompatibility, mass transport, mechanical strength)			
diagnostics		sensors (anisotropy, self-assembly, water sorption, mass transport, swelling)			
robotics		actuators and responsive materials (anisotropy, self-assembly, water sorption, mass transport, swelling)			
energy			separators, binders, and electrodes in batteries, batteries, energy harvest devices, and super capacitors (mechanical strength, water sorption, mass transport, surface charge, high surface area, self-assembly)		
flexible electronics	coatings on wearable devices (mechanical toughness controllability with water content, affinity toward cellulose-based fibers)				
textile	coatings on textiles (mechanical toughness controllability with water content, affinity toward cellulose-based fibers)				
packaging			gas barriers (high surface area, mechanical strength, structural integrity tunability with water, surface modification)		
membranes		liquid barriers (wet mechanical strength, surface modification)			
absorbents		site specific absorbents in membranes (wet mechanical strength, surface modification)		foams in hydrophobic and hydrophilic liquid superabsorbent (high surface area, porosity, surface modification, water sorption, mass transport)	
composites					nanofillers as reinforcement agents (wet mechanical strength, surface modification, high surface area)
food	rheology modifiers in processed food (water dispersibility, gel formation, shear-depending on viscosity, biocompatibility)				emulsifiers in processed food (amphiphilicity)
cosmetics	rheology modifiers in formulations (water dispersibility, gel formation, shear-depending viscosity, biocompatibility)				emulsifiers in formulations (amphiphilicity)

a This table summarizes the main areas of application of nanocellulose that are directly connected to the interactions with water. There are numerous details that are not reflected in this table.

#### 3.3.1 Hydrogels

**Figure 9:**
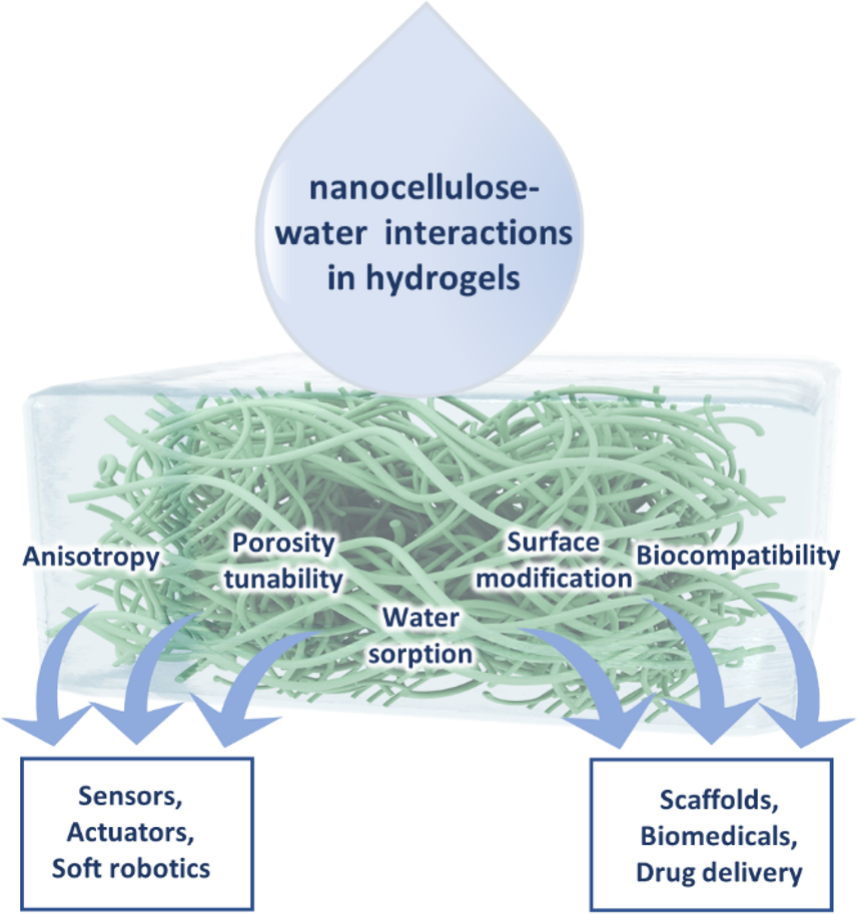
Summary of nanocellulose–water interactions in hydrogels.

**Figure 10:**
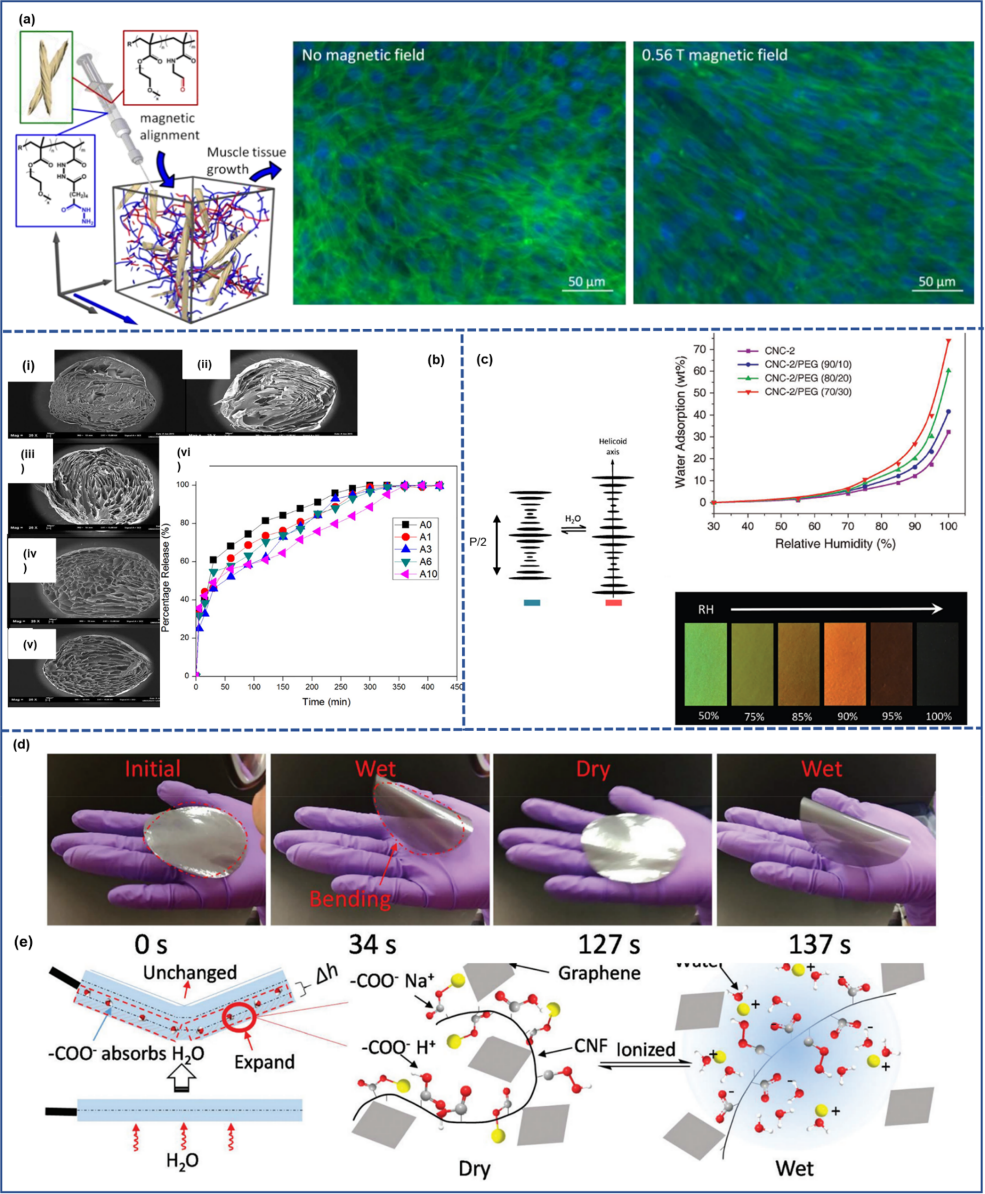
Nanocellulose–water interactions in hydrogels for biomedical applications (a,b), sensors (c), and actuators (d,e). (a) Schematic representation of an injectable CNC–poly(oligoethylene glycol methacrylate) (POEGMA) nanocomposite hydrogel with aligned CNCs directing the differentiation of skeletal muscle myoblasts into oriented myotubes in situ after culture for 8 days.^607^ (a) Adapted with permission from ref (607). Copyright 2017 American Chemical Society. (b) SEM images of aerogels produced by drying magnetic CNC and alginate hydrogel beads (i) 0% magnetic CNC (A0), (ii) 4.7% magnetic CNC (A1), (iii) 11.1% magnetic CNC (A3), (iv) 20% magnetic CNC (A6), (v) 33% magnetic CNC (A10), and (vi) time profile of ibuprofen release from alginate hydrogel beads. The presence of magnetic CNCs improves the physical and mechanical properties of the alginate hydrogel beads, increasing the swelling degree in water, and decreasing the rate of drug release.^608^ (b) Adapted with permission from ref (608). Copyright 2018 Elsevier. (c) Flexible and responsive chiral nematic cellulose nanocrystal/poly(ethylene glycol) composite films as humidity sensors.^609^ (c) Adapted with permission from ref (609). Copyright 2018 John Wiley and Sons. (d) Cyclic bending and recovery of a 90 mm diameter and 23 μm thick graphene/CNF nanopaper (15.2% graphene) upon exposure to two human breaths at 0 and 127 s. (e) Proposed folding mechanism of graphene/CNF nanopaper. Graphene flakes and CNFs in the nanopaper are held together by hydrophobic interactions among graphene flakes and hydrophilic and hydrogen bonding among CNFs. Upon exposure to moisture, the distances between CNF–CNF expand as hydrophilic and charge surface groups on CNFs become ionized by water. (d,e) Adapted with permission from ref (393). Copyright 2009 The Royal Society of Chemistry.

CNFs, including BC, inherently form weak, physical hydrogels via hydrogelation at low concentrations (0.5–1.5 wt %) owing to their flexibility and ability to entangle.^9,580,581^ In contrast to CNFs and BCs, the rod-like CNCs have a limited ability to entangle. Nevertheless, as described in section 1, CNC aqueous suspensions transition from liquid crystalline to birefringent viscoelastic phases at elevated concentrations.^571,582^ Altogether hydrogels are a widely reviewed^9,580,583,584^ class of nanocellulose-based materials. A recent review by Ajdary et al., in particular, gives a comprehensive view on nanocellulose-based hydrogels, inspired by nature toward advanced applications.^26^Figure 9 illustrates the overview of water interactions in hydrogels, their tuning, and their effect on different applications. As explained earlier, the ability of nanocellulosic hydrogels to bind and retain water is a direct result of the nanocellulose surface chemistry, aspect ratio, and flexibility, as well as of the 3D network microscopic and macroscopic structure. Water–network interactions and swelling are therefore related to processes on the molecular (hydration) and supramolecular (wetting, capillarity, and diffusion) level.^26^ However, excessive amounts of water can result in structural disintegration over time, especially in the presence of mechanical stress.^266,585−590^ The water ratio impacts the behavior of nanocellulose gels under mechanical stress and makes the prediction of this behavior difficult.^591^ Many of the nanocellulose hydrogels are formed physically by ionic interactions or physical cross-linking, which become weaker and eventually might fail with increased water content.^592,593^ Covalent cross-linking is the main strategy to minimize the detrimental effects of excessive water on hydrogel structural integrity (Figure 10a). The cross-linked hydrogels swell in excessive water and might change their shape, but normally these changes are less apparent than non-cross-linked hydrogels and the material usually shows better mechanical properties over time and under stress.^594−599^ Hydrogels of CNFs prepared by TEMPO-mediated oxidation have a hierarchical porosity in wet state in the meso- and macroporous range depending on their solid content. Subsequently, their mechanical properties and the characteristics of water within the nanofiber network depend on their hierarchical porosity.^314^

##### 3.3.1.1 Tuning Hydrogel Behavior through Water Interactions

Surface modification/functionalization^248,600,601^ and/or cross-linking^595,602,603^ have been widely used to tune the gelation behavior of nanocellulose and the network properties of their hydrogels. CNCs, for instance, form physically cross-linked nanofibrillar hydrogel networks in the presence of metal salts,^604^ with a sol–gel transition appearing at CNC concentrations far below (e.g., with 50 mM Ca^2+^ at 2 wt % CNC) the range of pristine CNCs.^571^ In the case of CNFs, cation cross-linking has been used to tune the mechanical properties of the hydrogels, i.e., via charge screening of the repulsive charges on the negatively charged nanofibril surfaces.^592^ Hydrogels made of hydrophobized cellulose have also been reported in an attempt to tune the hydrogel properties. Nigmatullin et al. synthesized CNCs that bind to each other through associative hydrophobic interactions. In this process, the sulfated CNCs were modified with hydrophobic moieties such as octyl groups. These octyl-CNCs form significantly stronger gels at lower concentrations than parent CNCs. Atomic force microscopy (AFM) studies revealed favorable interactions between remnant starch and octyl-CNCs, which is thought to be the source of the dramatic increase in gel strength.^605^ They also harnessed these hydrophobic interactions to assemble water-soluble macromolecules and nanoparticles into a transient hybrid network, forming thermosensitive hydrogels with tunable rheological properties.^606^

##### 3.3.1.2 Matrices and Carriers for Biological and Pharmaceutical Entities

As explained in section 1, water holds its pronounced role of being favorably involved in cellulose functions in nature along the dimensional hierarchy from macroplant down to nanofibers. Water is of paramount importance for the bioactivity in plant cell wall and plant growth. The physiological and biomechanical properties of lignocellulose are strongly influenced by the interaction of water with the biopolymer components within cell wall ultrastructure.^610−613^ Similar nanocellulose–water interactions, for example, freezing bound water mediated between the biopolymer matrix, and the surrounding water, contribute to the biocompatible functionality in several applications of nanocellulose outside their natural occurrence.^614,615^

As a result, nanocellulose has been applied in biomedical fields, such as drug delivery and tissue engineering (Figure 10a,b).^406,581,616−620^ In particular, nanocellulose-based hydrogels have attracted enormous interests to be utilized as a biocompatible substrate via different engineering technologies due to structural similarity with collagen.^621,622^ A large category of these systems are the hybrid hydrogels of nanocellulose and hydrophilic natural polymers such as hemicelluloses,^623−632^ pectins,^633−636^ lignin,^624,637−640^ chitosan,^641−653^ alginate,^575,608,654−666^ and gelatin.^661,667−673^ Whereas CNCs have been mostly used in hybrid hydrogels (Figure 10b),^27^ CNFs find wide-reaching applications as single-component systems, especially in the biomedical realm covering cell cultures, drug release, tissue engineering, and wound healing.^9,406^ Their high water content is an essential prerequisite for biocompatibility, and their nanostructure, porosity, and tunable mechanical properties can offer a biomimetic environment for cell immobilization and cell support.^674,675^ Moreover, network flexibility, porosity, and water content enable diffusivity, i.e., the uptake, transport, and release of low- and high-molecular-weight compounds, as exploited in controlled drug delivery (Figure 10.^676−680^

Besides hybrid hydrogels and single-component bulk CNF hydrogels, membranes, films, and microbeads based on swollen CNF networks, that is, somewhat geometrically constrained hydrogels, have been investigated in this context.^681^ For example, the effects of nanocellulose charge density and fibril size on the mechanical properties of the films in liquids were investigated. Swelling behavior of the films was studied in deionized water, in complete cell culture medium (DMEM), and in CaCl_2_ solutions. Cell culture media and CaCl_2_ solutions reduced the swelling of the films observed in deionized water, most probably due to a bridging effect (physical cross-linking) by the calcium ions. The reduction was proportional to the charge of the nanocellulose. The possibility to tune the softness of a surface by the level of oxidation can potentially be a way to influence cell behavior through mechanical cues.^682^ An emerging application of CNF-based hydrogels is support for microalgae with the aim of creating the next generation of solid-state photosynthetic cell factories. The combination of transparency, hydrophilicity and water retention, biocompatibility, good mechanical properties, and appropriate porosity ensures better performance than the alginates traditionally used as substrate.^683,684^ Furthermore, controlling water content and thereby binding of water on nanocellulose enables the manipulation of the diffusion of components within the hydrogel.^314^ This permits the regulation of diffusion of the photosynthetic products inside and out of the cell factory matrix.

##### 3.3.1.3 Anisotropic, Responsive, and Smart Materials

The specific response of nanocellulose to water is the direct functional foundation of a vast area of materials applications including humidity sensors and actuators. Precise tuning of the nanocellulose water-response, chemically or by physical means, enables, e.g., ultrahigh network swelling or water-response gradients in the nanoscale. As an externally effective trigger, water can be harnessed by sorption and desorption with cellulose chains bringing up multiscale movements for sensing.^685,686^

###### 3.3.1.3.1 Water Responsive Materials

The nanocellulose–water interactions have been used as a tool to tune mechanical properties. Capadona et al. developed responsive CNC-reinforced composites where the formation and disruption of a percolating CNC network was selectively and reversibly modulated via a response to water as a trigger. DMA analysis showed that the observed changes in modulus, elongation at break, and tensile strength are the result of switching off the cellulose–cellulose interactions by water molecules and not a simple result of a plasticizing effect. The authors concluded that the stiffness reduction achieved in the rubbery ethylene oxide–epichlorohydrin 1:1 copolymer nanocomposite is related to the decoupling of the stress-transferring rigid CNC network by water molecules as they competitively make hydrogen bonds. As a result, this switching is fully reversible, and the nanocomposite reverses to its original stiffness upon drying.^687^ Annamalai et al. studied the incorporation of cellulose nanocrystals into soft hydrophobic styrene butadiene rubber matrices to create water-responsive mechanically adaptive nanocomposites. In the dry state, all nanocomposites show higher tensile storage moduli than the neat styrene–butadiene rubber (SBR) or the SBR latex. Upon submersion in deionized water, a dramatic reduction of modulus was observed, which was connected to disengagement of the percolating CNC network due to mostly competitive hydrogen bonding of water molecules with the CNCs (solvation effects in the hydrophilic groups play also a role).^688^ Zhu et al. reported reversible formation and disruption of a CNC percolation network in an elastomeric thermoplastic polyurethane (PU) matrix that ultimately led to a rapidly switchable shape-memory effect (SME) activated by water. The researchers concluded that a combination of chemomechanical adaptability of the CNC percolation network and the entropic elasticity of the PU facilitates shape fixity for temporary deformation in the dry state and shape recovery in the wet state.^689^

Furthermore, hybridization with other natural or synthetic (macro)molecules (e.g., polymers, peptides) or nanoparticles has been a popular strategy to introduce multifunctionality or stimuli-responsive properties into nanocellulose-based hydrogels.^690^ The application prospects of these hybrids or multicomponent hydrogels are broad including, e.g., bioactive tissue scaffolds,^596,655,670^ ophthalmics,^691,692^ self-healing materials,^693,694^ high water containing fertilizers,^695^ and fingerprint detectors.^696^ The hydrogel network structure, in nanometer and micrometer scale, defines wetting characteristics, capillarity, and water diffusion. Moreover, the spatial orientation and distribution of (nanocellulosic) elements decides whether the network swells isotropically or in an anisotropic fashion, the latter paving the way toward swelling gradients and controlled movement. Several techniques exist for precise 3D design of hydrogel network structures, among them, 3D printing as probably the most popular one.

###### 3.3.1.3.2 Alignment, Anisotropy, 3D, and 4D Printing

**Figure 11:**
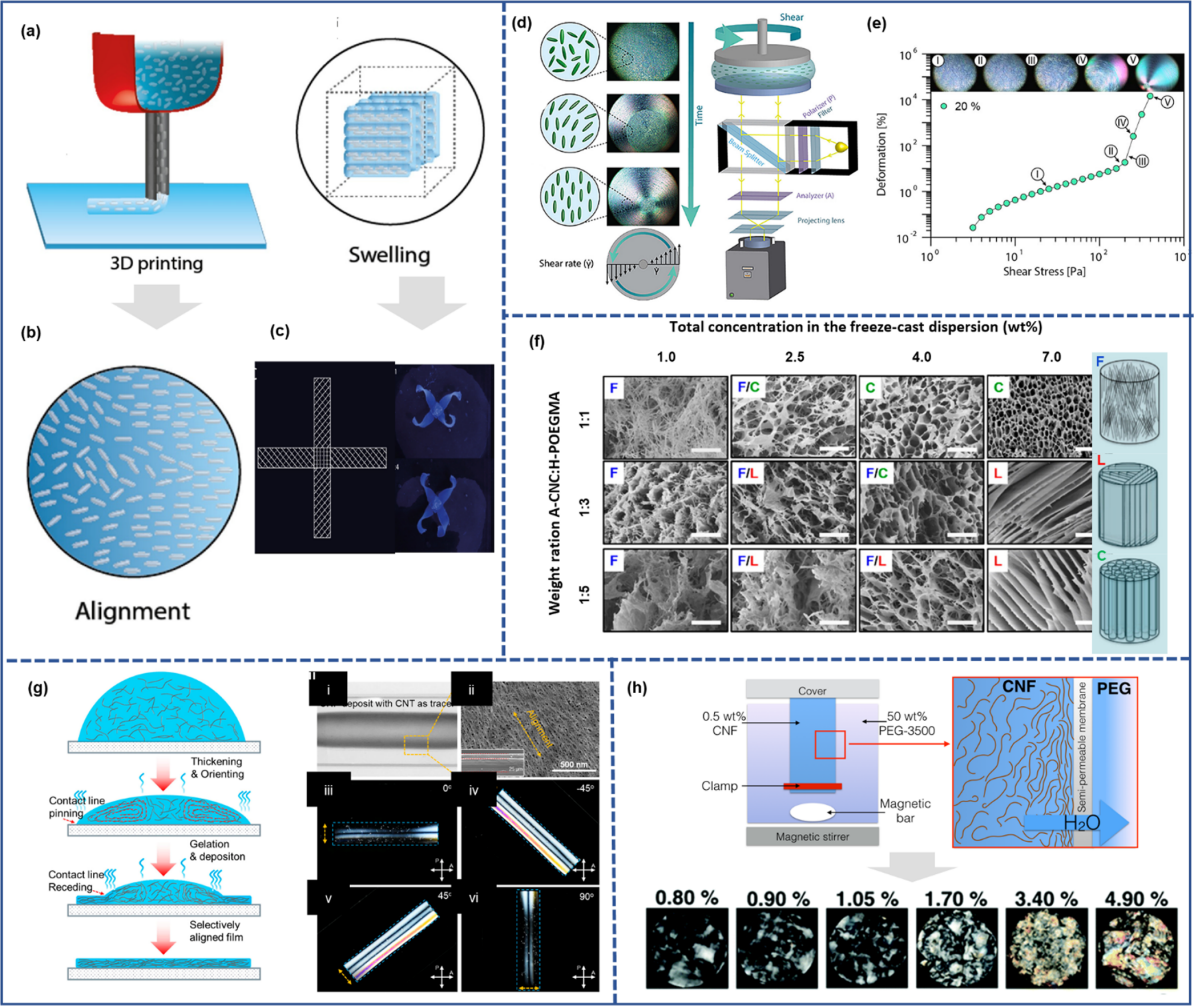
Nanocellulose–water interactions in anisotropic nanostructuring: (a,b) Schematic representation of CNC alignment while exiting the printer’s nozzle,^705^ (c) directional swelling of the material with aligned CNCs in water, used to create controlled water response.^705^ (a–c) Adapted from ref (705) under the terms of CC-BY. Copyright 2021 Elsevier. (d,e) In situ polarization rheology to study shear induced CNC alignment.^704^ (d,e) Adapted from ref (704). Copyright 2018 American Chemical Society. (f) SEM images of aerogels cross-section (the *XY*-plane perpendicular to the ice crystal-growth direction) with morphologies ranging from fibrillar (F) to columnar (C) to lamellar (L) and their combinations, dependent on A-CNC (aldehyde-functionalized cellulose nanocrystals):H-POEGMA (hydrazide-functionalized poly(oligoethylene glycol methacrylate)) weight ratio and A-CNC+H-POEGMA concentration. Scale bars are 20 μm. (right) Aerogels cast in cylindrical molds.^706^ (f) Adapted with permission from ref (706). Copyright 2016 American Chemical Society. (g) (left) Schematics showing the formation of a selectively aligned CNF film on a substrate. (right) Photographs of the CNF deposition with a few carbon nanotubes (CNTs) as a visible tracer on top of a transparent PET film.^707^ (g) Adapted with permission from ref (707). Copyright 2019 Elsevier. (h) Inducing nematic ordering of cellulose nanofibers using osmotic dehydration, images of the CNF suspensions with different concentrations between cross-polars.^121^ (h) Adapted from ref (121) under the terms of CC_BY. Copyright 2018 The Royal Society of Chemistry.

The transformation of digital design to on-demand manufacturing has been one of the predominant trends within the past decade and has established new technologies entering the market.^697^ Especially in the biomedical realm, additive manufacturing enables a customized design of, e.g., biomimetic tissues via tailoring the macroscopic hydrogel structure.^622^ CNC and CNF hydrogels inherently bear shear-thinning properties, which is a prerequisite for direct ink writing (DIW), the 3D printing technology of choice for hydrogels.^622^ Accordingly, the shear during extrusion induces an alignment of the nanoparticles introducing nanoscale anisotropy into the network (Figure 11a,b).^698,699^ This anisotropy is not only a very relevant attribute from a mechanical, thermal, and cell functional point of view, e.g., guiding cell differentiation in hydrogel-based biotissue mimics,^9,700^ but also in terms of directing the networks’ water response. Gladman et al. for instance, exploited the alignment during DIW of CNF embedded in a soft acrylamide matrix during DIW for the design of bioinspired shapes with anisotropic swelling characteristics, converting water-response gradients into a controlled mechanical movement.^701^ Gradient swelling dynamics of the shapes, thus, introduce a fourth dimension, and the transition of 3D to 4D printing has become an emerging trend in the realm of stimuli-responsive or multifunctional hydrogel systems with prospects, e.g., in soft robotics or biomedical devices (Figure 11d,e).^702^ In the case of CNC hydrogel inks, the alignment of the nanocrystals during 3D printing has been observed by a strong birefringence, confirming the anisotropy in the printed scaffolds (Figure 11a,b).^703^ Hausmann et al. made an attempt to understand the interplay between the concentration of CNC suspensions, the applied shear stress, and the dynamics of particle alignment using in situ polarization rheology.^704^ They showed that the precise adjustment of shear, extensional flow, and printing nozzle geometry can effectively tune the CNC orientation from full alignment to core–shell architectures (Figure 11a).

Freeze-casting is another way toward structural anisotropy (more explanation in section 3.3.3.1) (Figure 11f).^706,708,709^ Chau et al., for instance, prepared CNC-based hydrogels, starting from fabricating aerogels by simultaneous freeze-casting and cross-linking.^706^ The morphology of these aerogels, ranging from fibrillar, columnar, or lamellar, was thereby tailored by precisely controlling the composition of a CNC/poly(oligoethylene glycol methacrylate) precursor dispersion and the freeze-casting temperature. De France et al. used a strong magnetic field for the in situ alignment of CNC/poly(oligoethylene glycol methacrylate) nanocomposite hydrogels, which were simultaneously cross-linked via rapidly forming hydrazone bond formation, a promising approach toward injectable, anisotropic hydrogels for in vivo tissue engineering (Figure 10a).^607^ Another way to introduce anisotropy into nanocellulose suspensions was proposed by Guccini et al.^121^ The authors obtained induced nematic order in CNF suspensions over a wide CNF concentration range (0.5–4.9 wt %) by osmotic dehydration (Figure 11h). Figure 11 summarizes the attempts to prepare anisotropic nanostructures from nanocellulose.

###### 3.3.1.3.3 Actuators, Robotics, and Sensors

The controlled movement of hydrogels, realized via aligning the cellulose nanoparticles, as demonstrated by Gladman et al.,^701^ has an exceptional potential for hydrogel-type actuators, soft robotics, or sensor materials (Figure 10c–e). Nanocellulose-based biomimetic actuators that combine hydrophilicity gradients in the material with exploiting the fast dynamics^710^ of hydration have experienced an increasing popularity.^711−713^ Kuang et al., for instance, showed that CNFs align selectively upon solvent evaporation (Figure 11g). They used this self-assembly behavior to design extremely strong (∼1000 times lifting weight ratio) actuators with reversible shape-morphing properties upon hydration and dehydration.^707^ Zhu et al. used CNCs as active coating on poly(vinyl alcohol-*co*-ethylene) substrates driving humidity-induced actuation.^714^ These actuators could bend or twist depending on the CNC alignment direction. In a different example, Wang et al. showed that also asymmetric exposure of CNF thin films to water vapor can lead to controlled movement governed by the humidity difference across the film.^715^ Xu and Hsieh transformed the aqueous exfoliated graphene by amphiphilic nanocellulose into moisture-responsive foldable actuators, as discussed in section 2.4.3. The CNF/graphene film was easily obtained by vacuum filtration into nanopapers that exhibited rapid moisture triggered motion attributed to the highly accessible, charged CNF surfaces.^393^ Exploiting the responsiveness of nanocelluloses to water, CNCs and CNFs have been further explored as 1D, 2D, and 3D scaffolds for sensor applications,^716−718^ some of them for humidity sensing. Kafy et al., for instance, designed humidity sensors based on homogeneous CNC/graphene oxide (GO) composite films, in which changes of the surrounding relative humidity stimulated a change of the composite’s relative capacitance.^719^ Similarly, Solin et al. used a hybrid film of carbon nanotubes and CNF to detect humidity by a means of altered electrical resistivity due to accumulating water in the conductive film.^720^ Another principle toward nanocellulose-based sensors relies on the reversible swelling upon moisture contact and dehydration of chiral nematic CNC films coassembled with poly(ethylene glycol)^609^ or treated with *N*-methylmorpholine-*N*-oxide solution. This is accompanied by a color change, thus allowing easy detection of humidity changes (Figure 10c).^609,721^ Also exploiting the sensitivity of CNCs to moisture, Sadasivuni et al. developed a proximity sensor based on a graphene oxide-modified CNC (CNC/GO) sprayed in layers on polymer substrates bearing interdigitated electrodes.^722^ This setup offered a controlled proximity sensitivity without physical contact, which was investigated by measuring the current–voltage–resistance of the samples. The key for this application was the sorbed water on the CNC surface serving as an electron donor at low relative humidity, which changed the film resistance. At high relative humidity, the ionization of water to H_3_O^+^ ions contributed to the overall conductivity of the material. Additionally, the presence of CNCs in the composite improved the film structure and spatial resolution as an important structural prerequisite for a fast sensor response and signal recovery.

##### 3.3.1.4 Mechanical Load Bearing Hydrogels

Although nanocellulose hydrogels have water in their structure, the mere measurement of their mechanical properties does not give too much information on water interactions, and these studies will not be covered here.^9,583,594,596,723−728^ In many of these cases, nanocellulose was simply added to an existing hydrogel formulation to tune their water holding capacity (refs (641, 654, 655, 658, 691, 692, 729−735)).

###### 3.3.1.4.1 Turning Mechanical Properties of Hydrogels with Water

Water certainly plays an ambiguous role in the mechanical properties of nanocellulose materials. Structurally, with a trace amount of water, the crystallization of cellulose was increased with ordered molecular arrangement in disordered regions.^736,737^ Similar to other biopolymers, such as chitosan and collagen, the molecular packing of natural cellulose takes a more ordered structure in the presence of water.^736,738^ Consequently, the mechanical strength of cellulose increases in a characteristic amount of water. In a CNF hydrogel, water is not only considered a biocompatible element, but it also acts as a cross-linker for holding the gel integrity,^739,740^ up to a certain level after which the water is only loosely retained within the CNF network and not contributing to the swell of the nanofiber network.^314^ Beyond cellulose–cellulose adhesion, water can be essential for nanocellulose adhesion to other materials for producing a certain cellulose nanocomposite or a reinforced thermoplastic.^274^ However, water has also weakening effects on the nanocellulose hydrogels. It may cause the deterioration of mechanical strength over time by weakening cellulose–cellulose adhesion within the gel.^204,687,688^ At lower water contents, moisture can be used for a plasticization effect that enables the bending of wood material without breaking it.^741,742^ The same principle is utilized in the production of mechanical pulps from wood chips or logs.^743,744^

#### 3.3.2 Films, Membranes, Textile, and Coatings

**Figure 12:**
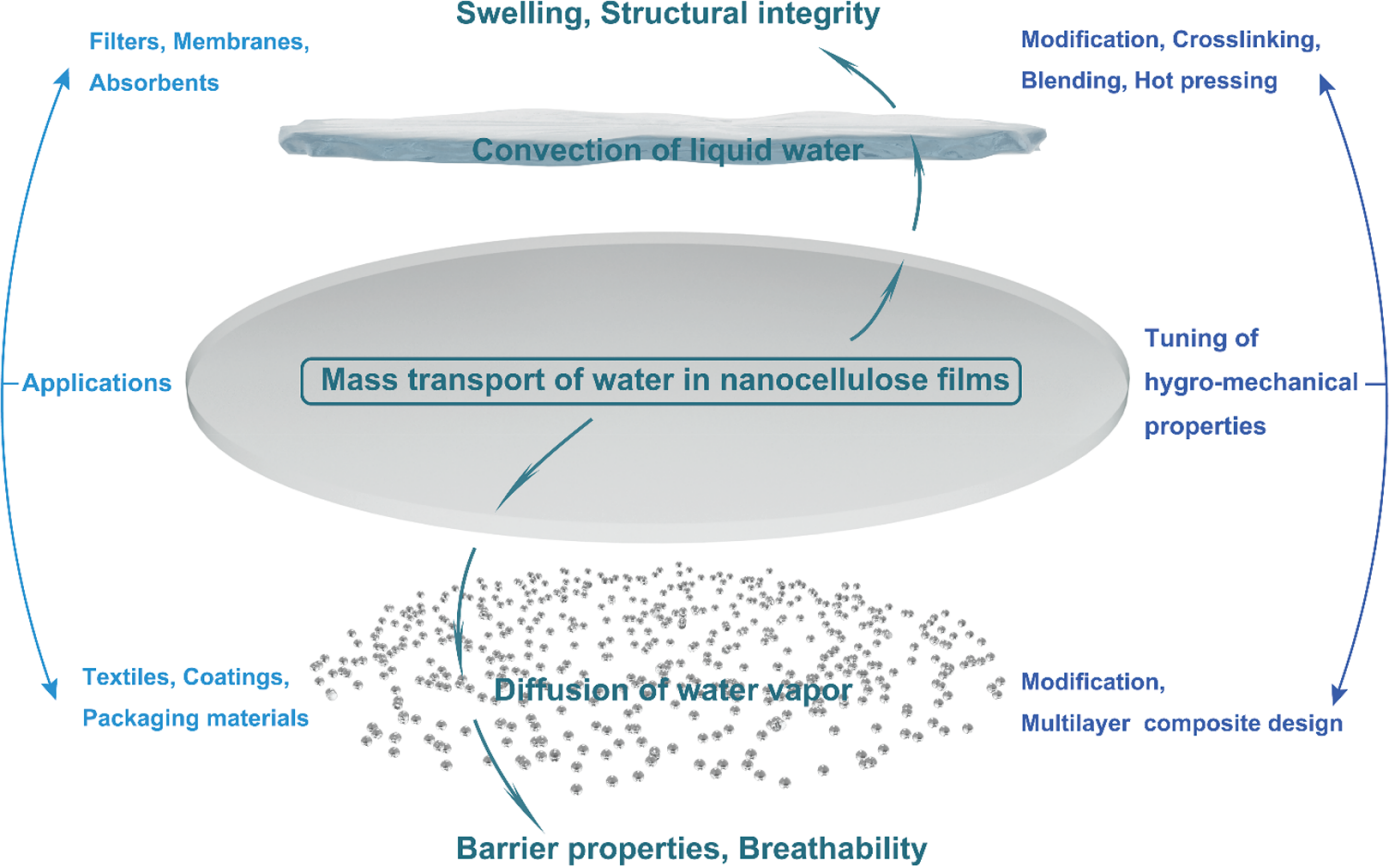
Summary of nanocellulose–water interactions in barrier films.

From a technical point of view, many films, membranes, textiles, and coatings are categorized as either hydrogels, aerogels, or colloids and could be discussed in sections 2.4.3, 3.3.1, and 3.3.3. Hybrid materials made by combining cellulose fibers and nanocellulose belong to this group as well. In the following section, we focus mainly on permeability and barrier properties of these materials and their application in packaging, filters, textile industry, and energy related and electronic devices with respect to water. As mentioned before, the availability of a large number of hydroxy groups in cellulose causes high water vapor adsorption and therefore increasing water vapor permeability and poor barrier properties.^298^ The effect of moisture on the mass transport of water within cellulosic materials is among the most crucial questions when the industrial use of (nano)celluloses is concerned. These effects are of importance in applications such as barrier films,^745^ absorbents,^746,747^ wound care products,^748^ biomedical materials,^692^ as summarized in Figure 12.

##### 3.3.2.1 Cellulose Nanopapers

Films of cellulose nanofibers are commonly known as cellulose nanopapers.^749,750^ Because of the high surface area of CNF and the density of the nanopaper network, the mechanical strength of air-dried nanopaper is outstanding, running up to 200 MPa in tensile strength.^751,752^ Contrary to conventional paper, nanopaper can also be transparent due to the nanoscale width of the CNFs.^753^ The proposed applications of nanopaper range from advanced packaging solutions to electronic supports.

Perhaps the most well-known effect of water on cellulosic materials is demonstrated when water accumulates on relatively dry networks, such as paper or nanopaper, whereupon an imminent strength loss is encountered.^754,755^ In technical terms, this represents a poor hygromechanical stability of the nanopapers. The mechanical properties of nanopapers depend on mastering structure formation processes and understanding interfibrillar interactions as well as deformation mechanisms of cellulose nanofibrils in bulk. Benitez et al. showed how different dispersion states of cellulose nanofibrils and different relative humidity values influence the mechanical properties of these nanopapers. The materials undergo a humidity-induced transition from a predominantly linear elastic behavior in dry state to films displaying plastic deformation due to disengagement of the hydrogen-bonded network and lower nanofibrillar friction at high humidity. A concurrent loss of stiffness and tensile strength of 1 order of magnitude was observed, while maximum elongation stayed near constant. Multiple yielding phenomena and substantially increased elongation in strongly disengaged networks, swollen in water, show that strain at break in such nanofibril-based materials is coupled to relaxation of structural entities, such as cooperative entanglements and aggregates, which depend on the pathway of material preparation. The results demonstrate the importance of controlling the state of dispersion and aggregation of nanofibrils in water by mediating their interactions and highlight the complexity associated with understanding hierarchically structured nanofibrillar networks under deformation.^756^

###### 3.3.2.1.1 Tuning Material Structural Integrity Using Water

Integrity of nanocellulose materials, which is perceived to play a role in mechanical disintegration of nanocellulose materials upon wetting is also strongly affected by water. Swelling is a detriment in many composites where the dimensions and the structure should be preserved, but it can be a benefit in applications such as 4D printing or biodegradable materials, where interaction with water and the change in shape is expected.

When it comes to preparation of flexible CNC chiral nematic films with optical and sensor applications, structural integrity and stability in water is important. Strategies such as cross-linking can limit swelling of the organized chiral nematic films and improve their structural integrity and stability in water and facilitate their use as freestanding template substrate for conducting polymers or metal oxides to form flexible chiral nematic photonic hybrids.^757^

A crucial challenge in characterization of nanocellulose materials is the lack of any standard practice on how to take the swelling into account when reporting mechanical properties at different relative humidity levels or when measuring the mechanical properties of fully hydrated materials, which limits comparisons between different studies. Walther et al. reviewed the current approaches and proposed a potential best practice for measuring and reporting mechanical properties of wet nanocellulose-based materials, highlighting the importance of swelling and the correlation between mechanical properties and volume expansion.^758^

###### 3.3.2.1.2 Improving Humid/Wet Mechanical Properties

Tremendous efforts have been taken to minimize the adverse effect of water on mechanical properties. Most of the studies with focus on improving mechanical properties of nanocellulose materials measure the strength, modulus, or ductility to assess the mechanical performance of the material in humid or wet state in comparison to dry state to confirm that the applied modification or blending techniques have improved hygromechanical stability. In this regard, the focus of these studies is mostly on the blending or modification they used to obtain waterproof or water-resistant materials rather than interactions of water and nanocellulose. Addressing these cases one by one is out of the scope of this review (some of them have been mentioned in section 3.1), and we refer the readers to the original publications for details.^444,759−761^ Here we focus on studies that provide deeper analysis of the interaction with water in their method to improve the hydromechanical stability.

Österberg et al. presented a rapid method to prepare robust, solvent-resistant, CNF films that can be further surface-modified for functionality by hot pressing the films. Drying of the films using high pressure and heat resulted in a film with good resistance to solvents. The films could be soaked in both polar and nonpolar solvents (including water) for more than 18 h. They swelled considerably in the solvents. However, their wet strength remained high, and they were easy to handle in the wet state.^760^ The enhanced properties are due to a decrease in the film porosity, which restricts the solvent diffusion through the film. In addition to the densification of the film, the hot-pressing affects the hydroxy groups at the surface, further restricting solvent penetration. This system results in structure-controlled hydrogels upon wetting that limits swelling and therefore the loss of mechanical properties by excessive water penetration.^760^ Shimizu et al. prepared TOCNF films, dried them, and soaked them in aqueous MgCl_2_, CaCl_2_, AlCl_3_, and FeCl_3_ solutions to change the counterion and form TOCNFs–COOM films. Dry TOCNF–COOM films showed high Young’s moduli and tensile strength. They found out that TOCNF films with aluminum and iron(III) carboxylates showed good mechanical properties in the wet state. These results are explained in terms of the high water resistance of the films, which is caused by the formation of interfibrillar electrostatic cross-linkages through multivalent metal ions, limiting swelling and consequent loss of structural integrity.^762^ Wang et al. pursued the design of ordered hard/soft nanocomposite structures with balanced supramolecular interactions for biomimetic applications. They established a drying procedure that induces a high orientation of CNCs in a matrix of carboxymethylcellulose (CMC) at high level of reinforcements (50 vol %). They showed alignment in thick bulk films and reported synergetic improvement with a simultaneous increase of stiffness, strength, and work of fracture as a function of the degree of alignment. They showed that the decline in the mechanical properties of such waterborne biobased nanocomposites at high relative humidity can be canceled out by using supramolecular modulation of the ionic interactions by exchanging the monovalent Na^+^ counterion, present in CMC and CNC, with Cu^2+^ and Fe^3+^. This leads to a synergetic improvement of the mechanical properties at 90% relative humidity.^763^

Ansari et al. reported on an interface tailoring route to prepare PEG-grafted CNF to address hygromechanical instability. Modified CNF nanopaper shows significantly improved mechanical properties under moist and wet conditions. Fracture surfaces of CNF films soaked in water showed distinct layers and fibrils pulled out in bundles, while modified CNFs showed a dense structure due to polymer grafting, protecting structural integrity and maintaining mechanical properties in water.^444^ Hakalahti et al. bonded TEMPO/NaClO_2_ oxidized CNF with poly(vinyl alcohol) (PVA) covalently to render water stable films. Pure CNF films and CNF–PVA films in the dry state showed similar humidity dependent behavior in the elastic region, while in wet films PVA had a significant effect on the stability and mechanical characteristics of the films. Influence of the amount and the degree of hydrolysis of PVA on the mechanical properties of the films were also investigated.^759^

Duran et al. partially modified CNFs by chemical means to create a shell of derivatized cellulose that surrounds the crystalline core of native cellulose. Through the different modifications, they aimed at creating a toolbox to tune the properties of CNF materials for specific demands. In total, nine different chemical modifications using different aqueous-based procedures were used as chemical pretreatments before CNF production through homogenization. Films produced from the different CNFs were mechanically tested, and it was found that a combination of periodate oxidation and borohydride reduction resulted in a high strain-at-break. The presence of carboxylic acids led to an increase in tensile strength and Young’s modulus, but a decrease in strain-at-break was also observed. The introduction of aldehydes, resulted in brittle films, but also a decrease in the moisture sorption rate, while the modulus even at high relative humidity was maintained.^445^

Zhang et al. suggested an approach for designing a water resistant, assembled nanopaper through controlled and irreversible aqueous complexation of oppositely charged cellulose nanomaterials. They produced cationic cellulose nanocrystals and tempo oxidized anionic cellulose nanofibers and adjusted the features of the nanopaper by altering the cationic CNC/anionic TOCNF ratio. the water resistance and water vapor permeability of obtained NC complexed nanopapers were improved. The full charge neutralization of oppositely charged NCs created a water-stable nanopaper with a wet strength of 11 ± 3 MPa after immersion in water for 24 h.^764^

Some scientists utilized nature inspired designs to waterproof nanocellulose films. For example, inspired by plant epidermis, Heredia-Guerrero et al. sprayed a cutin like coating, aleuritic acid, a polyhydroxylated fatty acid, on CNC films and polymerized the monomers by hot-pressing. Measurement of Young’s modulus and hardness and water uptake and water vapor transmission rate indicated that this design enhances the robustness and waterproof behavior of CNC films.^765,766^

##### 3.3.2.2 Films and Coatings as Barriers

###### 3.3.2.2.1 Liquid vs Gas Barrier in Nanopapers

As explained in section 2.4.2.4, transportation of gases and liquids in nanocellulose materials proceeds differently, and it is easier to block the flow of liquid water with its high cohesion and surface tension than that of individual water molecules in vapor phase. Water has generally detrimental effects on the barrier properties of nanocellulose. In the context of cellulose nanopapers, the intention is often to produce a barrier film, and the focus of the research field has largely been on how to improve the barrier properties in moist conditions.^745,755,767−771^ However, this has been proven to be a difficult task due to the tendency of cellulose to interact with and rearrange itself in the presence of moisture.^210,245,772−777^

**Figure 13:**
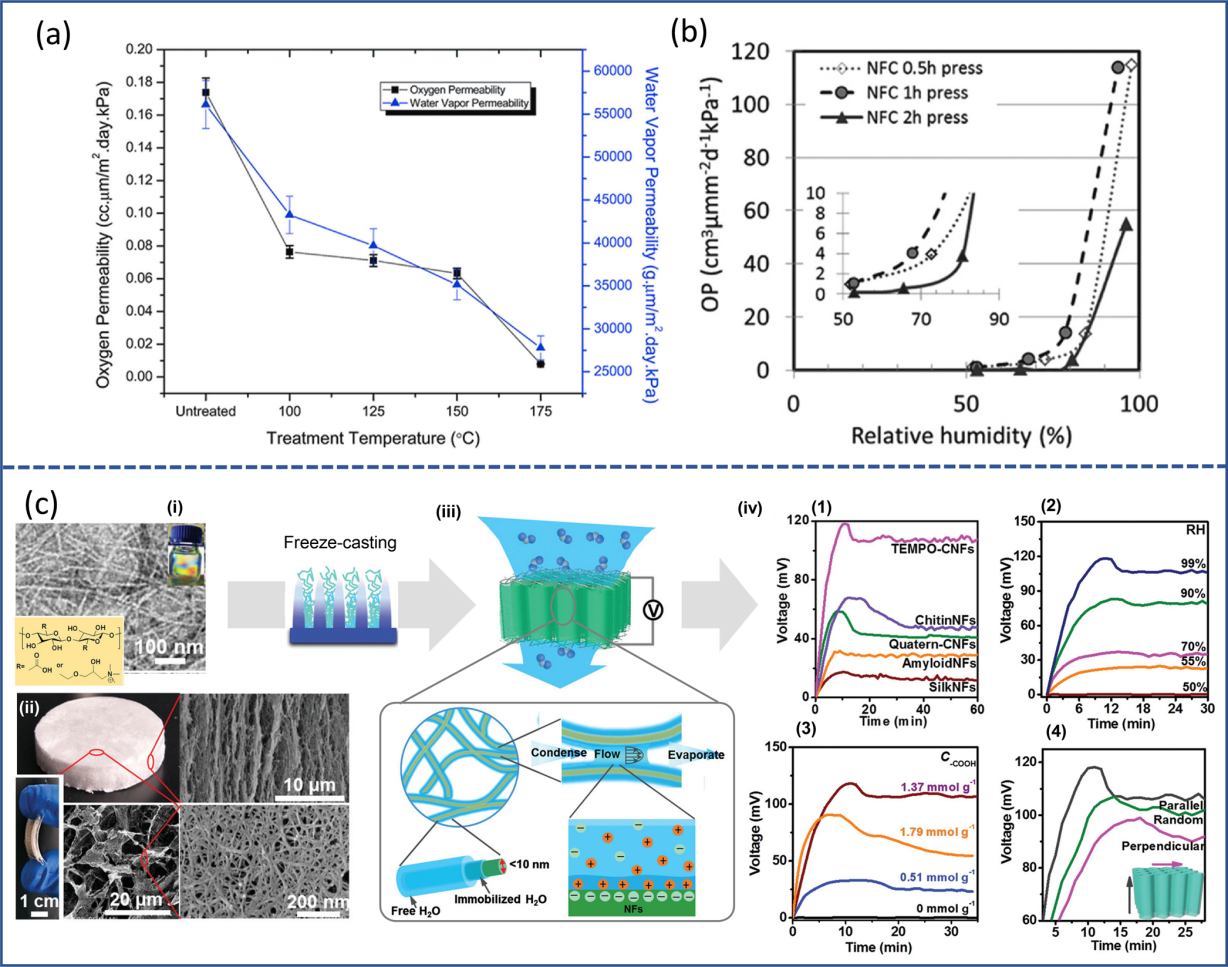
Nanocellulose–water interactions in films in packaging (a,b) and energy related applications (c): (a) Oxygen and water vapor permeability of untreated and thermally treated (100, 125, 150, and 175 °C for 3 h) CNF films,^798^ (a) Adapted with permission from ref (798). Copyright 2011 The Royal Society of Chemistry. (b) Oxygen permeability as a function of relative humidity for CNF films after varying thermal treatment times (0.5, 1, and 2 h) at 100 °C.^760^ (b) Adapted with permission from ref (760). Copyright 2013 American Chemical Society. (c) Electricity generation mechanism of biological nanofibrous generator: (i) Typical polarized photo of nematic CNF dispersion and TEM images of TEMPO–CNFs,^799^ (ii) optical and SEM images of aerogel fabricated by directionally freezing the CNF dispersions,^799^ (iii) schematic illustration of hydrated channels around and between TEMPO–CNFs with the carboxyl content of ≈1.39 mmol g^–1^ and the thickness of 2 mm in a nanogenerator.^799^ (iv) Open circuit voltage (*V*_oc_): (1) Generated by biological generators from different CNFs, (2) variation upon different relative humidity values of the air flow (flow velocity: 15 cm s^–1^), (3) variation upon different carboxyl contents of TEMPO–CNFs, (4) variation upon different air flow directions.^799^ (c) Adapted with permission from ref (799). Copyright 2019 John Wiley and Sons.

Nanopapers exhibit excellent oxygen barrier properties in dry state, and they have been candidates for food packaging for a long time.^778^ However, the strong hygroscopic character of the nanofibers limits their use in environments with high relative humidity (Figure 13a,b). Intercalation of water molecules between cellulose chains weakens the interfibrillar bonds between adjacent nanofibers, which leads to a decrease of the films’ barrier properties.^755,779,780^ Therefore, a common approach to use nanocellulose materials for packaging is to modify or blend them to increase the hydrophobicity of the surface of the material.^766,781−794^ Even some studies reported selective gas separation properties of these materials.^753,795−797^

###### 3.3.2.2.2 Strategies to Improve Nanocellulose Barrier Properties

Nanocellulose materials have been modified in different ways to make their barrier properties more resistant to moisture, (mentioned earlier in section 3.1.1). Polyethylenimine (PEI) surface functionalization, silylating, cross-linking with polyamide epichlorohydrin resins, cross-linking with chitosan, and compounding with montmorillonite are among numerous strategies that have been used to improve gas barrier properties at high relative humidity.^3^ Rodionova et al. carried out heterogeneous acetylation of microfibrillated cellulose to modify its physical properties, specifically improving barrier properties and at the same time preserving the morphology of cellulose fibrils for application in packaging.^800^ Kubo et al. mixed aqueous dispersions of CNF with sodium counterions (CNF-COONa) and CNFs with tetraethylammonium counterions (CNF-COONEt_4_) with various weight ratios. CNF-COONa/NEt_4_ films were prepared by casting and drying with different Na/NEt_4_ molar ratios. The film density, Young’s modulus, tensile strength, hydrophilic properties, and oxygen and water vapor permeability could be tuned by controlling the Na/NEt_4_ molar ratios in the films by the effect of bulky versus small counterion.^529^ However, it is important to note that mere bulk hydrophobization of the films is usually not enough to improve gas barrier properties. Generally, efficient water vapor barriers have not been achieved after bulk hydrophobization treatments that render the nanopapers nonwettable by liquid water.^801^ For example, chemical esterification of CNF with the aim of reducing bulk hydrophilicity and producing hydrophobic cellulose nanopaper with reduced moisture sensitivity has resulted in a decrease in barrier performances toward oxygen and water vapor.^755,779,780^ So far, the most efficient ways to block vapor transmission have been reported to include a (continuous) hydrophobic layer on top of the nanopaper,^801−803^ such as a applying a coating layer of hydrophobic cellulose via hornification or a coating layer of other hydrophobic polymers such as wax via deposition.^804,805^ In the context of food packaging the traditional strategy is to employ cellulosic materials in multilayered or sandwiched systems, which are effective due to isolation of the cellulosic materials from the humid environment and their protection from the effects of moisture.^760,806−812^

CNFs can also be applied as coatings to other cellulosic materials to improve barrier properties. Yook et al. prepared various types of CNFs coated on linerboard and wood-free paper to evaluate the barrier properties of these papers against air, liquid water, water vapor, oxygen, and grease. The average fibril size and hydrophobicity were strongly related to the barrier properties. CNFs with smaller fibril sizes and hydrophobized CNFs improved the water resistance. Air resistance and oxygen barrier properties and grease resistance were related to the average fibril diameter of CNFs.^813^

##### 3.3.2.3 Filters, Membranes, Textiles, and Absorbents

In the context of liquid sorption and permeability, the interactions between nanocellulose and water have been used to design materials as filters and membranes. There are numerous literature accounts on the application of nanocellulose materials as membranes. Besides the often-touted biobased “sustainability” aspect of nanocellulose, their more hydrophilic nature in comparison to common synthetic hydrophobic materials used for membranes, improves their resistance against fouling to some extent, not to mention that regenerated cellulose has been used for over a century to produce membranes and filters for different applications, including water treatment.^2^

Nanocellulose may increase the hydrophilicity of hybrid membranes to improve water diffusion, but this may reduce their mechanical properties. For polyethylenimine (PEI)-CNC films, for example, the Young’s modulus at relative humidity values of 30%, 42%, and 64% were found to be 16, 12, and 3.5 GPa, respectively.^814^ If the polymer that nanocellulose is mixed or modified with is highly hydrophilic, such as starch-based polymers, the nanocellulose actually decrease water sorption and diffusion. Using inherently hydrophilic polymers prevents the reduction of the mechanical properties but reduces the water flux through the nanocellulose membrane, simultaneously. Therefore, there seems to be a trade-off between the flux and mechanical strength of nanocellulose composite membranes.^64,815,816^

Network diffusivity, combined with offering sorption sites for pollutants, are key for the use of nanocellulosic absorbent filters in environmental engineering applications^816,817^ and oil and gas production.^818^ Secondary surface modification, cross-linking, or hybridization with other natural or synthetic polymers enables pollutant-specific membrane designs, with enhanced sorption capacities and rates, as well as mechanical and structural integrity, e.g., to withstand water flow for an extended time.^4,450,819−821^ The sorption of organic contaminants from water has also been demonstrated with modified nanocellulose matrices. The inherent hydrophilicity of nanocelluloses should be reduced to improve the affinity of the material for hydrophobic compounds. Increasing the hydrophobicity for this purpose has been achieved by inclusion of both organic and inorganic functionalities. Xiong et al., for example, designed flexible and multifunctional CNF membranes, coated with titanate–bismuth oxide for the synergistic treatment of anion/cation-containing oily water.^822^ In another study, atomic layer deposition of TiO_2_ nanoparticles onto the surface of nanocellulose aerogels created a low-energy surface on the fibers to yield a hydrophobic and oleophilic material that can absorb oil and a variety of organic solvents from the surface of water at a capacity of 80–90% vol/vol.^823^ Similarly, depositing triethoxyl(octyl)silane or poly(dimethylsiloxane), or freeze-drying methyltrimethoxysilane onto CNF aerogels resulted in even higher sorption capacities.^512,824,825^ Graphene oxide/nanocellulose composites and poly(dopamine)/BC membranes also showed good sorption capacities.^816,826,827^

A distinctive field of applications of nanocellulose that might require water repellence in some cases is the textile industry. Colloidal dispersions of CNFs are good alternatives to dissolution/regeneration into cellulose II in filament spinning. Cunha et al. investigated the effects of postmodification of wet-spun CNF filaments via chemical vapor deposition of organosilanes with different numbers of methyl substituents. Various surface structures such as continuous and homogeneous coating layers, or three-dimensional and hairy-like layers, reduced the surface energy, which significantly affected the interactions with water. Mechanical testing revealed that the wet strength of the modified filaments were almost 3 times higher than that of the unmodified precursors, while the final product maintained the moisture buffering capacity and breathability.^828^

##### 3.3.2.4 Energy Storage Devices

Nanocellulose-based mesoporous structures, flexible thin films, fibers, and networks have been often used in photovoltaic devices, energy storage systems, mechanical energy harvesters, binders, separators, structural supports, and catalyst supports, demonstrating the potential of this material in several energy-related fields.^3,829−842^ The presence of surface-adsorbed water opens completely new perspectives for nanocelluloses in electronics.

###### 3.3.2.4.1 Aqueous Ion Batteries

Nanocellulose attracted numerous interests in serving as a promising building block for electrolyte wettable and thermally resistant separators as an alternative to synthetic polymers in gel polymer and solid polymer electrolytes in diverse rechargeable battery systems including established Li-ion and Li–S to next generation Na-ion, K-ion, and Zn-ion systems.^842,843^ Nanocellulose is also capable of inducing an increase in viscosity of electrolytes solutions even at minuscule concentrations. High porosity offered by nanocellulose network as electronically insulating physical barrier is desirable in separator design to ensure good electrolyte retention and enhanced ionic conductivity as well as prevent internal short-circuit. Nanocellulose also holds a prevalent role of enabling good affinity for liquid electrolytes ensuring preferential interaction with the salt anion to improve salt solubility and cation transference in the electrolyte. Mittal et al. combined CNFs and CNCs constructed a mesoporous hierarchical structure as gel polymer electrolytes, ensuring a close contact with metallic Na outperforming conventional fossil-based separator in sodium ion batteries.^844^ However, the detrimental effect of water on mechanical properties of nanocellulose networks used in energy storage applications, the effect of water on degradation of lithium salts in Li-ion batteries (LIBs) is a concern. Different strategies have been reported to address this issue and unlock the potential that nanocellulose offers in terms of mechanical properties, environmental benignity, and versatility. The manufacturing process of CNFs is essential to improve the performance of CNFs as both an electrode component and a separator. Flexible paper electrodes were also simply obtained using as low as 4 wt % CNFs (prepared by TEMPO-mediated oxidation) as binder.^845^ Such low amount minimizes the moisture introduced to the cell and increases the capacity of the electrodes by total weight. A further study by Lu et al. investigates the optimal processing parameters to enhance the cathode in terms of mechanical and electrochemical performance, achieved using high surface charge (ca. 1.5 mmol g^–1^) and low defibrillation degree.^845^ Using a facile paper-making method, Kim et al. assembled CNFs prepared by TEMPO-mediated oxidation into asymmetric mesoporous separators that represented a more environmentally benign approach compared to conventional fossil fuel-derived materials.^846^ Furthermore, the performance of CNF based separators were crucially improved by substituting the native Na^+^ ions of the carboxylate groups and adding 2 wt % of vinylene carbonate to remove Na^+^ deposition and suppress gas evolution.^847^ In addition to solvent exchange to address the issue of Li salt degradation in LIBs, Jabbour et al. and Leijonmarck et al. suggested that prolonged thermal treatment of cellulose papers can also solve this problem.^848,849^ Generally, cellulose, with its intrinsic chemical and physical properties and microstructures, offers a perfect matrix for manufacturing environmentally friendly LIBs. The good compatibility of cellulose with water allows the utilization of aqueous electrolytes instead of hazardous organic solvents, and the safety concern of LIBs can therefore be greatly alleviated.^850^ Water can also be used to remove unwanted chemicals in the process of binder preparation. Natural cellulose dissolved in ionic liquids can be used for production of binders. After the preparation of cellulose solution in ionic liquid and mixing graphite and LiFePO_4_ (LFP) for anode and cathode, respectively, and coating the slurry on metal foils, the electrodes can be immediately immersed in water to extract the ionic liquid. Water is miscible with ionic liquids but does not dissolve cellulose. In this phase-inversion process, the ionic liquid is completely recyclable.^851^

###### 3.3.2.4.2 Fuel Cells

Recently, Vilela et al. have reviewed the use of nanocellulose-based materials in polymer electrolyte fuel cells.^577^ In such application, the high-water affinity coupled with the mechanical properties of nanocellulose are an excellent combination. Compared to the commercial ionomer Nafion, nanocellulose-based membranes can be fabricated thinner without compromising their gas barrier properties, a factor that can potentially decrease the cell resistance. Bayer et al.^852^ incorporated CNF and CNC nanopapers into membrane electrode assemblies replacing the commonly used and expensive ionomer membrane Nafion or nanocellulose/Nafion composites.^853,854^ The researchers showed a superior performance of the nanocellulose membranes in the fuel cells at high operating temperatures up to 80 °C, with a continuous increase of conductivity with the temperature.^852^ Moreover, the conductivity of CNF and CNC membranes increased sharply with the relative humidity as water acts as a charge transport medium. A decisive role was also given to the surface charge groups (−COO^–^ for CNF and −OSO^3–^ for CNC) functioning as proton acceptors and donors. CNC-based membranes showed good conductivity and gas barrier properties, attributable to their higher surface charge density and crystallinity. As in the case of LIBs, the physicochemical properties of the nanocellulose are crucial in terms of aspect ratio and surface charge of CNFs in their performance as polymeric electrolytes. The decrease in conductivity between 80 and 70%RH, as described by Bayer et al., can be suppressed by using nanofibers with a high amount of surface water to produce well-defined and homogeneous membranes, in particular with thin nanofibers (ca. 2 nm) and high surface charges (ca. 1.5 mmol/g).^855^ In the same study, Guccini et al. achieved a proton conductivity exceeded 1 mS cm^–1^ at 30 °C between 65 and 95%RH, which is 2 orders of magnitude larger than with previously reported nanocellulose materials.^852,856^ Furthermore, despite being 30% thinner, a lower hydrogen crossover than with conventional Nafion membranes was observed, likely given by the combination of the excellent mechanical properties of CNFs and the homogeneous membrane structure.

###### 3.3.2.4.3 Hydrovoltaic Effect

The interaction of nanocellulose with water can generate electricity, a phenomenon which has been denoted as hydrovoltaic effect.^857^ In brief, this effect is based on different water activities, including diffusion, evaporation, and flow, which generate water gradients through solids. An electrical pulse results from the concentration gradient of H^+^ ions.^858^ The ability of nanomaterials, including the eagerly advertised carbon nanomaterials, to harvest electric energy from flowing water and moisture, is based on their exceptional sensitivity toward adsorbed species, providing the ideal 1D nanospace for water binding and a rapid transport.^859^ In a recent contribution, Li et al., translated the hydrovoltaic effect to naturally derived nanomaterials, including CNFs, for harvesting energy from moist air flow (Figure 13c).^799^ They have shown that, analogous to ion channels of cytomembranes, these bionanomaterials can capture moisture from air through hydrated nanochannels due to their inherent hygroscopic properties and surface charges. Accordingly, in a continuous air flow, the dynamic balance between water adsorption and evaporation produced a streaming potential through the aerogel membranes resulting in an open-circuit voltage. Nanocellulosic materials have also been used in solar evaporation systems. A typical interfacial solar evaporator includes a photothermal layer on top, which upon solar irradiation converts light to thermal energy (heat). It also includes a support whose role is to simultaneously provide hydrophilic channels that continuously wick water via capillary forces to the hot layer to generate steam. Nanocellulosic materials have been used as supporting substrates in these systems.^860^

###### 3.3.2.4.4 Solar Energy Harvesting Devices

Solar energy-harvesting devices require high surface area and good charge transport properties so that photons can be absorbed and converted into electrical energy. CNF 3D mesoporous structures offer a very large surface area and present good mechanical properties.^861^ Therefore, CNFs can be an attractive template/matrix for processing structures used in photoelectrochemical (PEC) electrodes.^862^ PEC water splitting is a promising strategy for directly converting solar energy into hydrogen fuels.^863^ Rapid charge generation and separation, large surface area, and broadband light absorption are important aspects of PEC development^862,864^ and nanocellulose based 3D structures can offer a medium for these technologies, due to their water absorption and water retention capacity.^865−868^ The hydrophilic mesopores within the cellulose film could serve as an ideal host matrix for the embedment of the nanoparticle catalysts with minimized agglomeration.^866^ The mesoporous cellulose films can be used as a framework for photosynthetic and photoactive material and to organize the redox couples. The water retention capacity of the mesoporous 3D cellulose nanostructures plays a crucial role in the reactions and processes in these systems because water is the medium for these devices.^867,869^

###### 3.3.2.4.5 Supercapacitors

An important application of CNC-derived porous carbon materials is supercapacitors that are closely connected to the interactions of nanocellulose and water. In the first step, self-assembly of CNCs in an aqueous environment results in the development of a chiral nematic phase. This structure can be further carbonized to form chiral nematic mesoporous carbon.^147,870,871^ MacLachlan and co-workers synthesized composite films with chiral nematic structures by the evaporation-induced self-assembly of CNC with silica precursors. After pyrolysis and etching of the silica, freestanding films of chiral nematic mesoporous carbon were obtained, which were used in symmetrical capacitor with H_2_SO_4_ as the electrolyte.^872^ CNCs and CNFs can be integrated together to form porous carbon materials for supercapacitors as well.^873^

###### 3.3.2.4.6 Flexible Devices

Nanocellulose can be easily manipulated to make stable physical or chemical bonds with other cellulosic materials such as fibers used in textiles. This characteristic has been used to design flexible electronic devices. Hu et al. has constructed breathable structures with decoupled electrolyte and oxygen gas pathways for Li-oxygen batteries using carbon nanotube-coated nanocellulose cotton textile as a flexible substrate and electrolyte reservoir.^874^ Other than Li-oxygen batteries, nanocellulose macrofiber-based textiles have also demonstrated great potential in constructing flexible supercapacitors and lithium-ion batteries.^875,876^ Water is almost always used as the dispersion medium for nanocellulose materials that would be filtered to form positive or negative electrodes in the preparation of flexible electrodes.^848,877−881^ In a typical process to prepare conductive polymer/nanocellulose hybrids by in situ polymerization, the nanocellulose particles are dispersed in a mixture of acid and water to produce a suspension. Oxidant, initiator, and monomers of conductive polymers are then added to the suspension and the polymerization occurs under mild stirring.^882−890^ Water also plays an important role in full recyclability of the nanocellulose used in the conductive flexible material. In the first report of a flexible, transparent, and metal-free triboelectric nanogenerator that is naturally degradable after 60 min stirring, the aluminum-doped zinc oxide/CNF paper was completely dispersed into the water. The final dispersion was clear and was used to form a CNF gel with the small amount of Zn and Al ions coming from the aluminum-doped zinc oxide coating. This coating can be redispersed in water and further concentrated and filtered to produce CNF film again, which indicates an excellent recyclability.^891^ Despite the positive roles of water in these devices, one of the main detrimental effects of water on the energy related applications of nanocellulose is also observed in the case of wearable devices. The washability of these materials exposes them to large amounts of water that can threaten their integrity and function.^842^

#### 3.3.3 Powders, Aerogels, and Foams

**Figure 14:**
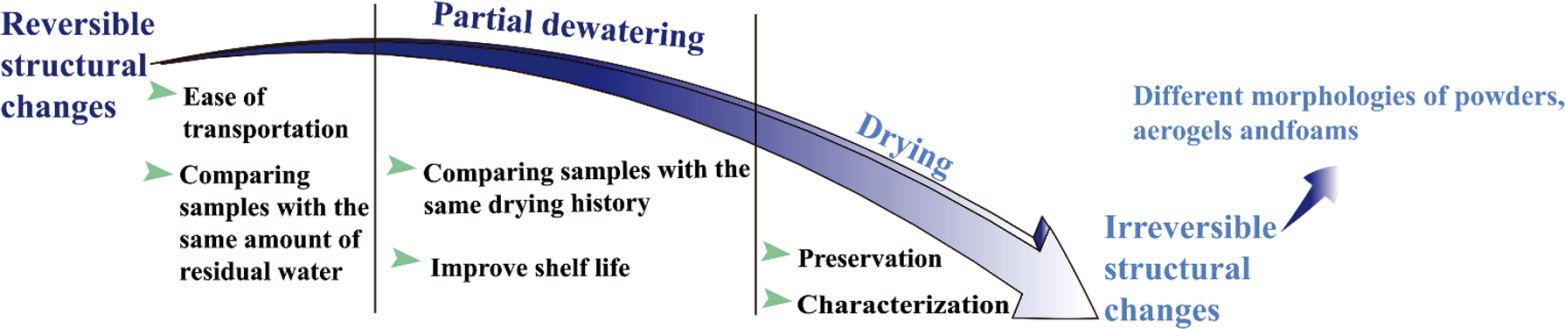
Summary of nanocellulose–water interactions in dewatering process.

Water is usually partially or mostly removed from colloidal dispersions or hydrogels of nanocellulose to produce new diverse products such as powders, aerogels, and foams, to extend the shelf life of the material, to omit the intrusive effect of water on characterization techniques, or simply to facilitate easier and cheaper transportation (Figure 14).^21,299^ The extremely high surface area present in nanocellulose in comparison to other cellulosic materials can be lost easily during removal of water from the water–nanocellulose systems. Thus, an entire family of novel dewatering techniques have been developed especially for the nanocellulose–water systems to minimize this effect.

##### 3.3.3.1 Tuning Dried Nanocellulose Morphology by Water Removal Techniques

**Figure 15:**
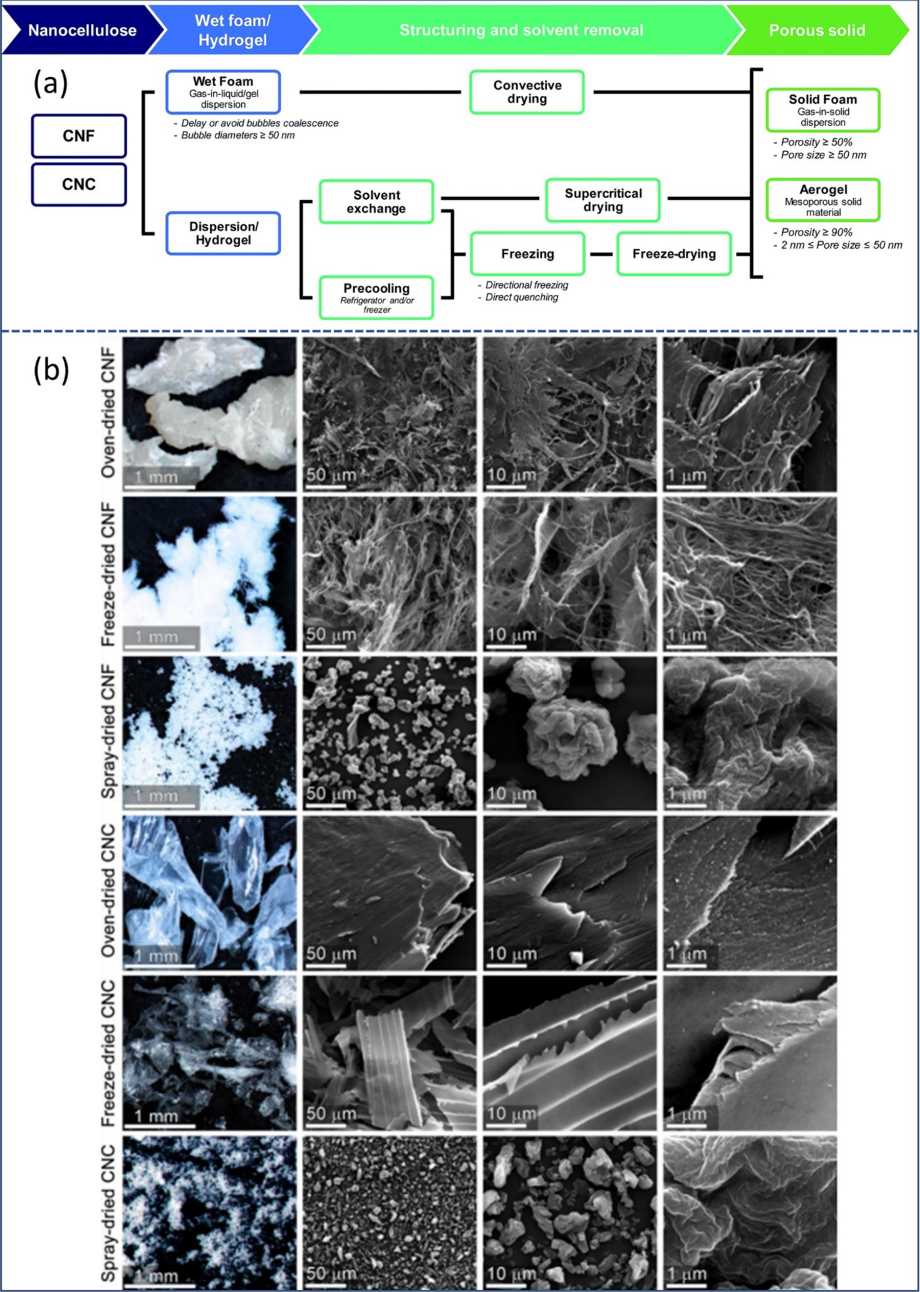
(a) From nanocellulose to nanocellulose-based foams and aerogels: terminology and processing.^320^ (a) Adapted from ref (320) under the terms of CC_BY. Copyright 2017 The Royal Society of Chemistry. (b) Effect of drying method on the morphology of nanocellulose materials. This figure is produced with permission from ref (898). (b) Adapted with permission from ref (898). Copyright 2020 American Chemical Society.

As mentioned before, CNCs can be freeze-dried, spray dried, supercritically dried, oven-dried, or freeze-spray dried, and the drying techniques are known to have an effect on the properties of the product (refs (315, 321, 323, 327, 554, 565, 892−897)). However, the difficulty of removing water from nanocellulose suspensions, while retaining the nanoscale properties of the nanocellulose is a substantial challenge. Lavoine et al. have reviewed the methods to prepare nanocellulose-based foams and aerogels, exhaustively (Figure 15a).^320^ In a review by Sinquefield et al. on the current state of nanocellulose dewatering and drying methods,^299^ the authors present SEM images showing the representative morphology of CNCs and CNFs dried by oven, spray drying, and freeze-drying. Figure 15b illustrates the effect of water removal technique on the morphology of dried nanocelluloses.

For both CNCs and CNFs, oven drying produced the largest agglomerates. Samples of oven-dried CNC and CNF retained some nanoscale surface textures but resulted in a lower surface area than the other drying methods. Spray drying results in aggregates with a wide range of sizes in both CNFs and CNCs. The CNF spray-dried particles were larger than the CNC particles. The nanoscale surface textures of both CNF and CNC spray-dried particles appeared similar.^299^ Wang et al. used supercritical drying to preserve the original gel structure and network for CNC aerogel preparation and showed that the produced aerogels have a nanoporous network structure and high specific surface area.^899^ The products of freeze-drying are usually networked multiscalar structures.^315,377^ Freeze-dried CNFs maintain at least partially the nanoscale features with reduced fibril agglomeration relative to oven-dried samples.

**Figure 16:**
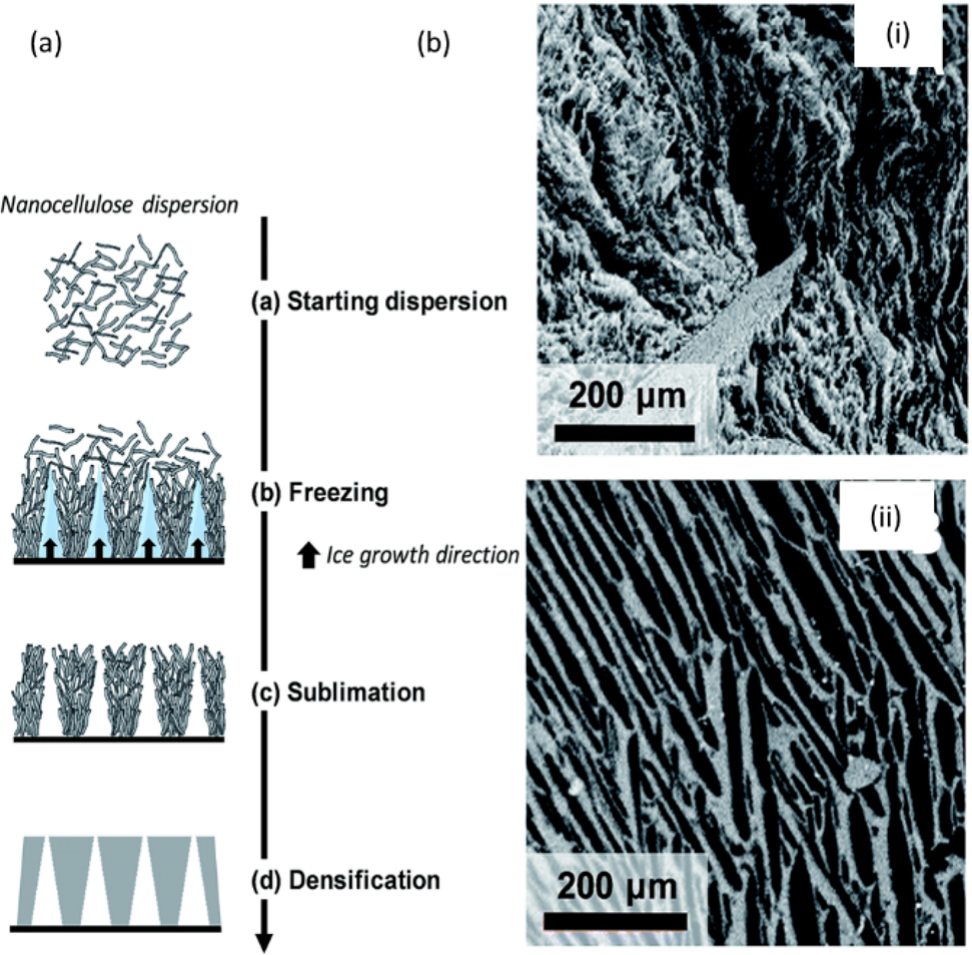
(a) Schematic diagram of directional ice-templating of CNC dispersion. (a) Adapted from ref (320) under the terms of CC-BY. Copyright 2017 The Royal Society of Chemistry. (b) SEM images of the horizontal cross-section of nanocellulose foams obtained by (i) homogeneous freezing and (ii) unidirectional ice templating. (b) Adapted with permission from ref (902). Copyright 2016 American Chemical Society.

The morphology of freeze-dried CNCs can be templated by ice formation during the freezing process which can be adjusted by freezing rate, initial dispersion concentration, and including additives. On the other hand, aerogel preparation with uniform pore size is a formidable challenge (Figure 16a,b).^320^ Han et al. studied the self-assembling behavior of both CNFs and CNCs during freeze-drying. Within a certain range of concentration, the fibrils self-aligned into a lamellar-structure foam composed of aligned membrane layers. The authors described the mechanism of these assemblies, again, as a result of ice crystal growth. When the stable nanocellulose suspension was frozen, ice crystals gradually grew in the same preferred direction and created a lamellar structure oriented in the direction of the freezing front. Nanocellulose was expelled and separated by ice crystals and formed a morphology templated by ice crystals (Figure 18a).^321^ Huang et al. reported that when the solid content in the original suspension of CNF varied between 4 and 10 wt %, the morphology of the aerogel transformed from the membranous to fibrillar network, increasing its specific surface area and redispersibility, while further dehydration was detrimental. They attributed this phenomenon mainly to the entanglement of cellulose fibrils in the cellulose network, which suppressed the growth of ice crystal during the process. The interaction between intra/intermolecular hydrogen bonds was also responsible for the variation of the cellulose morphologies.^894^ The ice templating effect during freeze-drying was also reported by Deville, and the mechanism of ice templating and nanocellulose behavior during freezing was studied in detail,^322^ although their study did not include the drying step. Lewis et al. used cyclic physical confinement of CNCs between growing ice crystal domains to promote aggregation of CNCs. Freeze thawing (FT) cycling was employed to form larger aggregates of CNCs without changing the surface chemistry or ionic strength of the suspensions.^900^ They showed that the rheology of CNC suspensions can be tuned by FT cycling for suspensions with 4% or more concentration. The complex modulus of 4 wt % CNC suspensions after each FT cycle shows a significant increase, reaching a plateau finally. SEM images of the freeze-dried CNC aerogel formed by this method demonstrated the formation of an interconnected, porous cellulosic sheet network.^900^

In the case of BC aerogels, Gromovykh et al. investigated the effect of culturing conditions on the structure and anisotropy of the produced bacterial cellulose aerogels and found out that ice crystal templating is not the only mechanism for formation of layered morphology of BC aerogels. They suggested apart from the dewatering techniques, geometry, source of carbon and nitrogen, and oxygen availability of the aqueous culture influence the anisotropy and structure of the produced bacterial cellulose network and subsequently result in different morphologies in the formed aerogels.^901^

Some efforts have been taken to decrease the detrimental effects of water crystal growth on dispersion stability, and subsequently, the structural uniformity of the aerogel. For example, freeze-drying from water dispersions can result in significant CNF aggregation. If the aggregation is not desired, especially for dispersibility or surface area reasons, solvent exchange to *tert*-butanol prior to freeze-drying can be helpful.^492,903,904^ Saito et al. converted CNF aqueous suspensions into hydrogels, which were afterward solvent exchanged to *tert*-butanol and freeze-dried in order to obtain aerogels with less aggregations.^905^

Peng et al. investigated the effect of drying method on thermal stability and crystallinity of the dried products. Supercritical-drying produced CNFs with the least thermal stability and the lowest crystallinity index. Air-drying or spray-drying produced CNFs which were more thermally stable compared to freeze-dried CNFs. The different drying methods resulted in various char weight percentages at 600 °C for the dried CNFs or CNCs. The dried CNFs are pure cellulose I, while the dried CNCs consist of cellulose I and II.^327,901^

##### 3.3.3.2 Dewatering of Nanocellulose for Characterization and Preservation

###### 3.3.3.2.1 Effect of Water Content on the Results of Characterization Methods

As mentioned briefly in section 3.3.2.1, a very important challenge in the field of nanocellulose characterization is the difference in the analytic data based on the difference in water content. It is important to compare the qualities of different nanocelluloses reported in different studies, only with the same dewatering/drying history. Nanocellulose materials hold residual moisture contents of approximately 2–5 wt %^327^ in their powder form, which can greatly increase the difficulty of analyzing results for many characterization techniques, such as specific surface area measurements and some processing conditions like melt compounding, which imposes serious issues in their applications.^21^ By annealing at 100 °C in vacuum, most moisture can be removed but nanocellulose readsorbs water immediately upon coming into contact with the atmosphere.^330,331^ In practice, characterization techniques such as DVS of celluloses in general, but especially of nanocelluloses, are greatly influenced by the treatment and drying history of the material, e.g., whether air-dried from water or from another solvent,^126^ by freeze-drying,^318^ or by supercritical drying.^21^ The structural changes affect the moisture uptake and its retention in the material, and therefore special care should be devoted to the method of sample preparation to obtain relevant information on its water uptake properties.

###### 3.3.3.2.2 Dewatering for Preservation

As mentioned in section 2.2.2, drying of the cellulose source material before nanocellulose isolation procedure, results in hornification and can result in CNFs or CNCs, with different characteristics than the ones produced from never-dried cellulose sources.^906^ More critically, drying the already produced CNFs and CNCs irreversibly alters their characteristics mainly due to pronounced hornification, as stated on several occasions in this review. However, keeping large amounts of water in the nanocellulose samples causes many problems such as transportation difficulties, storage problems, and vulnerability toward fouling. Dewatering methods (not fully drying) are a compromise to minimize the complexities of having large quantities of water in the system without completely drying the samples. Nanocellulose is typically stored and transported as a gel with a nominal solid content of up to 5 wt % to avoid interfibril hornification, which means a large amount of water should remain in the gel. There are strategies to reduce the volume of nanocellulose gels for preservation and transport. For example, Reverse dialysis in poly(ethylene glycol) (PEG) has been shown to be a safe dewatering method via osmotic dehydration, without causing irreversible aggregation and sample heterogeneity.^907^ Santmarti et al. used low molecular weight PEG as a replacement for the water phase in nanocellulose aqueous gels. These gels had solid contents of up to 70% without interfibril hornification.^554^ Despite such reports, the majority of research laboratories and industries report preservation of nanocellulose in water. Some studies have focused on accelerating the dewatering process by changing the pH and ionic strength of the dispersion.^908^ According to the suggestions for best practices in storing nanocellulose materials, all nanocellulose in wet state should be stored in the refrigerator or small amounts of sodium azide or toluene should be added to the suspensions. Dried powders should be stored under low temperature and humidity conditions. However, if the nanocellulose materials are intended to be used in toxicity testing or biomedical applications the use of antimicrobial agents is prohibited, and dewatering, refrigeration, or a combination of both are the only secure ways to store the materials.^21^

#### 3.3.4 Reinforcing Nanofillers in Composites

**Figure 17:**
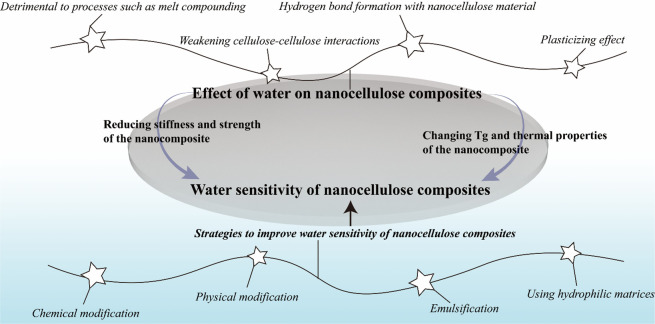
Water in nanocellulose-reinforced polymer composites.

One of the major potential industrial applications of nanocellulose particles, CNFs and CNCs, is their use as reinforcing fillers for nanocomposites due to the inherently high mechanical strength of crystalline cellulose. One of the critical parameters for polymer composites, which governs the properties of the material, is the compatibility of the interfaces between constituting components. The interface is particularly important for nanoscale components because of their immense surface area (refs (12, 119, 247, 344, 430, 449, 452, 467, 470, 473, 474, 477, 480, 483, 486, 540, 551, 909−924)). In this section, we are not delving into the vastly researched topic of how to disperse nanocellulose fillers as efficiently as possible within the continuous matrix in polymer composites. Rather, we investigate the interactions of water and nanocellulose reinforcing fillers, which have been used in polymer nanocomposites and describe the effect of these interactions on the applications (Figure 17). For the full review on strategies on how to solve the issues in nanocellulose applications in polymer nanocomposites, the readers are referred to numerous available reviews.^64,470,477,925−935^

##### 3.3.4.1 Water Sensitivity in Nanocellulose Composites/Blends

**Figure 18:**
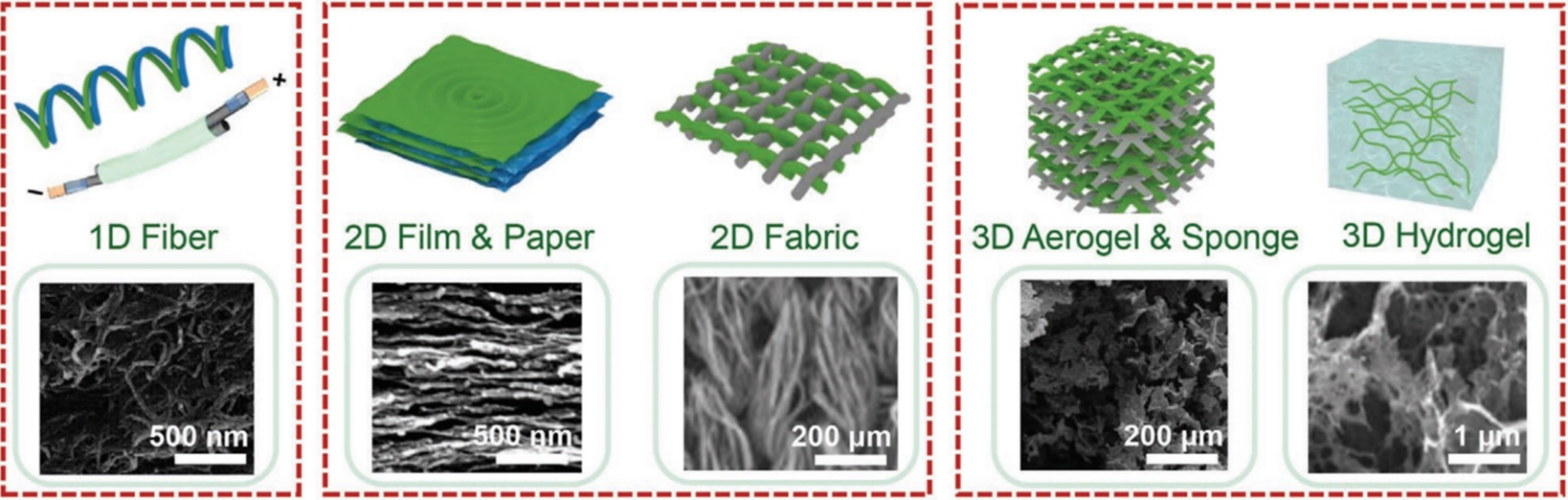
Schematic illustrations of various structures of nanocellulose composites (including 1D fiber, 2D film, paper, and fabric, and 3D aerogel, sponge, and hydrogel).^398,835,937−939^ Adapted with permission from ref (835). Copyright 2019 John Wiley and Sons. SEM figures were originally published in refs (398, 937−939). Reproduced with permission from ref (398). Copyright 2015 John Wiley and Sons. Reproduced with permission from ref (937). Copyright 2018 Elsevier. Reproduced with permission from ref (938). Copyright 2017 John Wiley and Sons. Reproduced with permission from ref (939). Copyright 2017 Elsevier.

**Figure 19:**
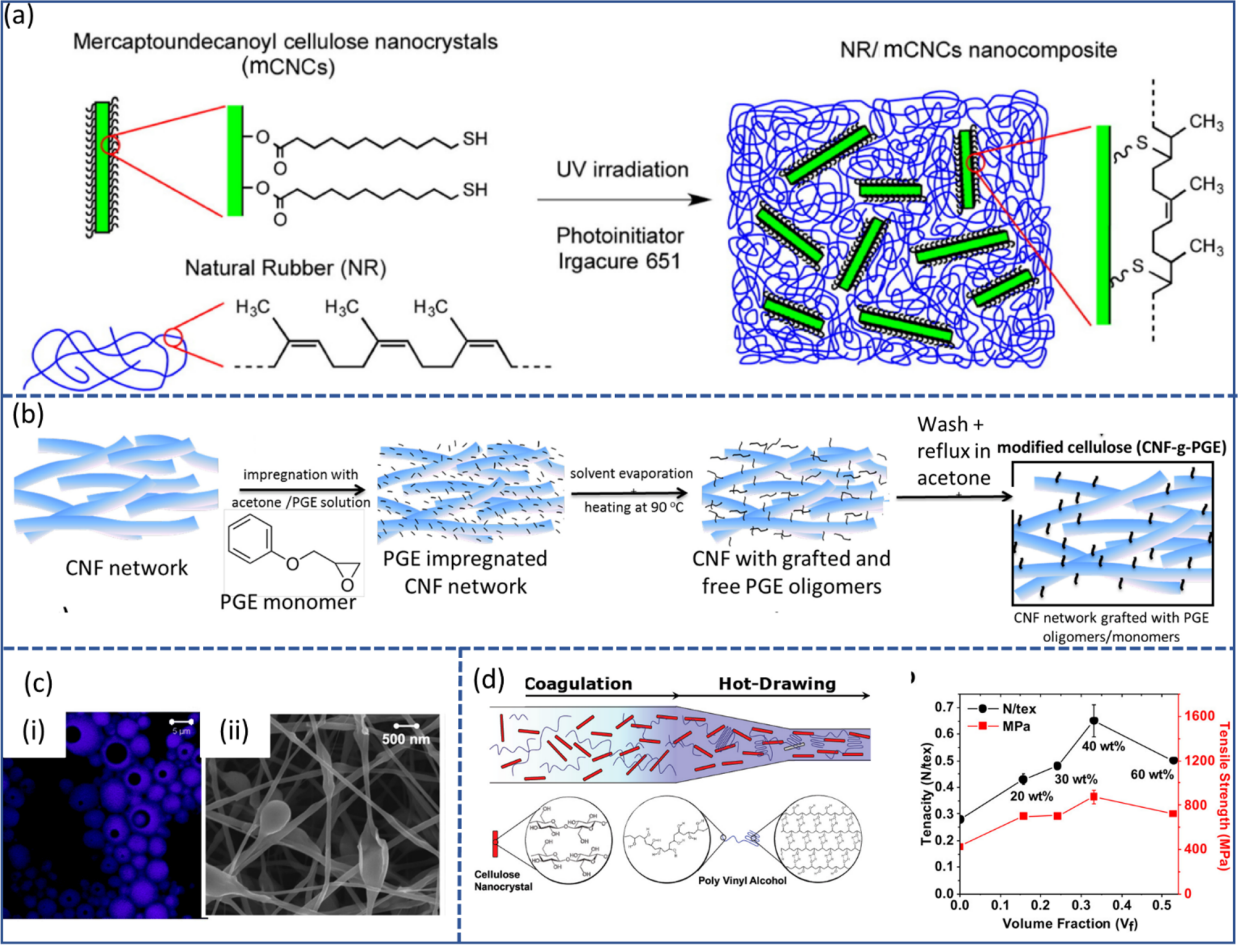
Strategies to minimize the detrimental effect of water in nanocellulose reinforced composites. (a) Surface modification: Illustration of the synthetic route and structure of natural rubber (NR)/mercaptoundecanoyl-modified CNC (m-CNC) nanocomposites.^936^ (a) Adapted with permission from ref (936). Copyright 2015 American Chemical Society. (b) Cross-linking: Schematic of the steps to graft phenyl glycidyl ether (PGE) on CNF, where PGE monomers were impregnated in a CNF network and the reaction was initiated thermally. The free oligomers formed at this stage were removed by extensive washing with acetone.^444^ (b) Adapted with permission from ref (444). Copyright 2016 Elsevier. (c) Emulsification: (i) Images obtained by confocal fluorescence microscopy for emulsions containing poly(styrene) (PS) in toluene and CNFs in the aqueous phase and emulsified at a PS:CNF dry mass ratio of 90:10, (ii) SEM images of electrospun nanofiber mats prepared from double emulsions containing PS and CNF (90:10) with surfactant mixture concentration of 3.^940^ (c) Adapted with permission from ref (940). Copyright 2016 The Royal Society of Chemistry. (d) Using hydrophilic polymers: (i) Illustration of fiber processing to optimize the microstructure of CNC/poly(vinyl alcohol) (PVA) composite fibers: (1) Coagulation of spinning dope (CNC and PVA) by injection into a coaxial flowing stream of coagulant; (2) hot-drawing of the fiber under tension at high temperature (150 °C). Comparative mechanical strength of all CNC/PVA composite fibers and the pure PVA control in textile and materials units. The strength reached 880 MPa, exceeding the properties of most other nanocellulose based composite fibers previously reported.^941^ (d) Adapted with permission from ref (941). Copyright 2016 American Chemical Society.

There are many potential applications where adding an easily dispersible reinforcing nanomaterial to polymer matrices results in nanocomposites, with the advantages such as improved mechanical properties.^485^ These nanocomposites can be manufactured in 1D (fibers), 2D (films, nanopapers, fabrics), and 3D (aerogels, foams, and hydrogels) forms tailored toward different applications (Figure 18).^835^ Most polymers used in nanocomposites are more hydrophobic than CNFs or CNCs and, in consequence, there are usually compatibility issues between the components. Besides the compatibility problems, the main drawback in application of nanocellulose in polymeric matrices is their sensitivity to water, which has a profound effect on dispersion, wetting, interfacial adhesion, matrix crystallization, water uptake, and hydrothermal stability (see sections 3.1.1 and 3.1.2). Nanocellulose surfaces are rich in hydroxy groups, which absorb significant amount of water under moist conditions. Surface water molecules weaken the cellulose–cellulose interactions, act as plasticizers, and reduce the network stiffness and strength.^444^ Apart from compatibility issues water could be also detrimental to the processing and product performance in some techniques such as melt compounding for nanocomposites.^21^ Addition of CNFs or CNCs to nanocomposites may also affect the glass transition temperature (*T*_g_) of the material. This effect, although not reported for all nanocellulose nanocomposites, is shown especially in the case of moisture-sensitive systems. Plasticization effects of water, whose concentration can be increased by the presence of nanocellulose, and the strong interaction between water and the matrix can be the main reasons for this effect. Change in *T*_g_ has a potentially important effect on some applications of these nanocomposites that require thermal stability,^74^ and nevertheless, it is an issue that must be taken into account. Therefore, a large number of studies aim at improving the outcome of nanocellulose applications in nonaqueous polymeric systems and the key to these approaches is usually surface modification (Figure 19a)^936^ and cross-linking (Figure 19b).^444^

###### 3.3.4.1.1 Reducing Water Sensitivity of Nanocellulose Composites/Blends

Several attempts have been taken to modify CNCs and CNFs in composites physically or chemically to address water sensitivity. Models have been developed to investigate the effect of modifications on water–matrix interaction to predict the final materials performance of a nanocomposite structure. Lyubimova et al. performed MD simulations of neutral and negatively charged sulfated CNC in water and employed a statistical mechanical molecular theory of solvation to evaluate the solvation structure and thermodynamics of the relaxed CNC in ambient aqueous NaCl solution. This model predicts molecular recognition interactions in solution and can be used to improve the compatibilization of CNC with matrix polymers to enhance the CNC loading levels in composites. The method was shown to be able to accurately predict the degree and type of CNC surface modifications necessary to achieve a good dispersion in polymer solutions while preserving the desired crystallinity and mechanical properties.^942^ Chemical and physical surface modification, counterion change, and emulsification are among approaches that have been used to improve the qualities of nanocellulose–polymer composites. Wei et al. used computational approaches to obtain understanding of water adsorption and interfacial mechanics of modified CNC surfaces to address the issues regarding the response to moisture. They found both experimentally and theoretically that methyl(triphenyl) phosphonium (MePh_3_P^+^)-exchanged CNCs have lower water uptake than Na-CNCs due to the disruption caused by the bulky ionic structure of MePh_3_P^+^. The adsorbed water accumulates near the cations and is oriented by electrostatic interactions as well as water–water hydrogen bonding. Traction–separation behavior of these interfaces is highly dependent on the surface chemistry. MePh_3_P^+^ cations serve to change the interface in a way that it exhibits hydrophobic behavior, such as formation of capillary bridges and preservation of mechanical properties upon wetting. The researchers showed that chemical surface modification is a viable option for changing the adsorption and traction–separation behavior of CNC as an important first step toward the design of moisture-tolerant CNC–polymer nanocomposites.^943^ Carrillo et al. proposed double emulsion systems for the compatibilization of aqueous dispersions of CNFs with a nonpolar polymer matrix (Figure 19c). Nonionic surfactants were used in CNF aqueous dispersions equilibrated with an organic phase. This method of CNF integration within hydrophobic polymers removed the need for drying or solvent-exchanging of the CNF aqueous dispersion prior to processing, proving double emulsion systems as a novel, efficient, and scalable platform for CNF coprocessing with nonpolar systems in nanocomposite preparations.^940^

##### 3.3.4.2 Hydrophilic Nanocellulose Composites

Due to the hydrophilic character of nanocellulose, the simplest polymer systems that incorporate nanocellulose are water-based systems. In these systems, water plays a positive role. Nanocellulose dispersions can be simply mixed with aqueous polymer solutions or dispersions (both natural and synthetic polymers).^944^ Although these systems suffer from limited applications and are only appropriate for water-soluble or dispersible polymers such as latexes,^74^ they can be very useful in the scope of colloids, emulsions, hydrogels, films, membranes, 3D printable, and responsive materials, as discussed in section 3.3.1, 3.3.2, and 3.3.3 (refs (9, 584, 641, 642, 654, 675, 705, 727, 732, 733, 945−949)). For example, cross-linked CNF/poly(acrylic acid) (PAA) composites have been prepared in order to improve the material properties in humid environments.^950,951^ Nanocellulose/PVA composites have similarly been popular.^941^ In fact, Lee et al. managed to prepare a fiber composite that bears one of the highest reported tensile strength values of all nanocellulose-based fiber composites, ca. 900 MPa (Figure 19d).^941^ But such materials with all-hydrophilic components are usually susceptible to moisture and water, which possesses an intrinsic constraint for their usage.

## 4 Analytical Tools to Probe Nanocellulose–Water System

### 4.1 Computational Methods to Uncover Water–Nanocellulose Interactions

MD simulation is an appropriate tool for performing atomistic computer simulations of moleculear interactions within material itself, with other molecules, and with solvents under given thermodynamic conditions. Both water and cellulose have been studied by MD simulations. For both of these materials, some parallels appear in simulation studies and experimental results. Simulations allow the creation of hypothetical measurements and the predicted results for these measurements. These simulated test results are then compared with real experimental results, and the results of other simulated measurements with different assumptions and conditions, to both understand the real structure of the material, and the origin of the experimental signal.

The first atomistic MD simulations of cellulose–water interface were performed already in the late 1990s by Andreas Heiner and Olle Teleman, who found that the interactions with water gave rise to structural disorder in the first cellulose layer, with respect to the crystal lattice parameters.^228,952^ Since then, the importance of using computational modeling for the understanding of cellulose and cellulose–water interactions has grown rapidly. Specifically, developments in molecular modeling methods such as MD and quantum mechanical density functional theory (DFT) have been instrumental for this advancement and has recently been reviewed by Bergenstråhle-Wohlert and Brady,^953^ Zhang et al.,^954^ and by Buehler and co-workers.^955^

While MD is based on classical physics and treats atoms as point particles that are interacting pairwise through empirical potentials, DFT takes the electronic configuration into account and is therefore considered very close to an ab initio method. This has consequences for its practical use. While DFT is more precise, it is only efficient in systems consisting of a few molecules, whereas MD currently can treat systems of millions of atoms and reach millisecond simulation times. In addition, there has been considerable effort in developing coarse-grained potentials for cellulose simulations, in which atomistic details are sacrificed for the benefit of significantly extending available time- and length-scales.^956,957^ Therefore, in these models, the details such as hydrogen bonding and water molecular orientation are averaged out, and they are less suitable for specific investigations of cellulose–water interaction on the molecular scale.

There exist several empirical force fields for atomistic MD that are specifically designed to treat carbohydrates in aqueous solution. Among the most popular are CHARMM (Chemistry at Harvard Macromolecular Mechanics),^958,959^ GLYCAM (developed and maintained by Complex Carbohydrate Research Center at the University of Georgia in Athens GA),^960^ GROMOS (GROningen MOlecular Simulation computer program package),^961^ and OPLS-AA (Optimized Potentials for Liquid Simulations-All Atom) force field (developed and maintained at Purdue and Yale universities),^962^ which are all frequently used for simulation studies of cellulose. The potentials are developed in close connection with a water model, which thus can be considered part of the force field. The GROMOS potentials uses the SPC (simple point-charge) water model,^963^ while CHARMM, GLYCAM, and OPLS-AA are developed with the TIP3P (a 3-site rigid) water model.^964^ In spite of the relative simplicity of these models, they have proven to be accurate enough for average properties related to hydrated cellulose systems. In fact, one study which used a more complex water model that included electronic polarizability reported only minor differences compared to static models.^965^ For a detailed comparison between the performances of different water models, the reader is referred to the literature.^966^

#### 4.1.1 Cellulose Is Insoluble in Water

From a fundamental perspective the nature of cellulose is ambiguous. Owing to its large number of hydroxyl functionalities, it is considered a hydrophilic molecule. On the other hand, it is completely insoluble in water at ambient conditions (Figure 5). It has been suggested that this phenomenon is due to the many intermolecular hydrogen bonds of the aggregated structure. This view was contested by Lindman and co-workers,^967,968^ who argued that cellulose is an amphiphilic molecule and that aggregation is a consequence of hydrophobic assembly. Indeed, early MD simulations had shown that the contribution from hydrogen bonding to the dissolution free energy is an order of magnitude smaller than from dispersion interactions and the hydrophobic effect.^969,970^ The latter was later explicitly shown to originate in a large entropic penalty of the water in the first hydration shell.^971^ The entropic cost was however significantly reduced at elevated temperature and pressure and as a consequence cellulose was soluble in simulations of water at supercritical conditions.^972^ The entropic cost associated with water can also be mitigated by addition of cosolvents such as urea,^973,974^ which is exploited for enhancing cellulose dissolution in cold alkali^256^ (Figure 5).

#### 4.1.2 Cellulose Twist in Water

**Figure 20:**
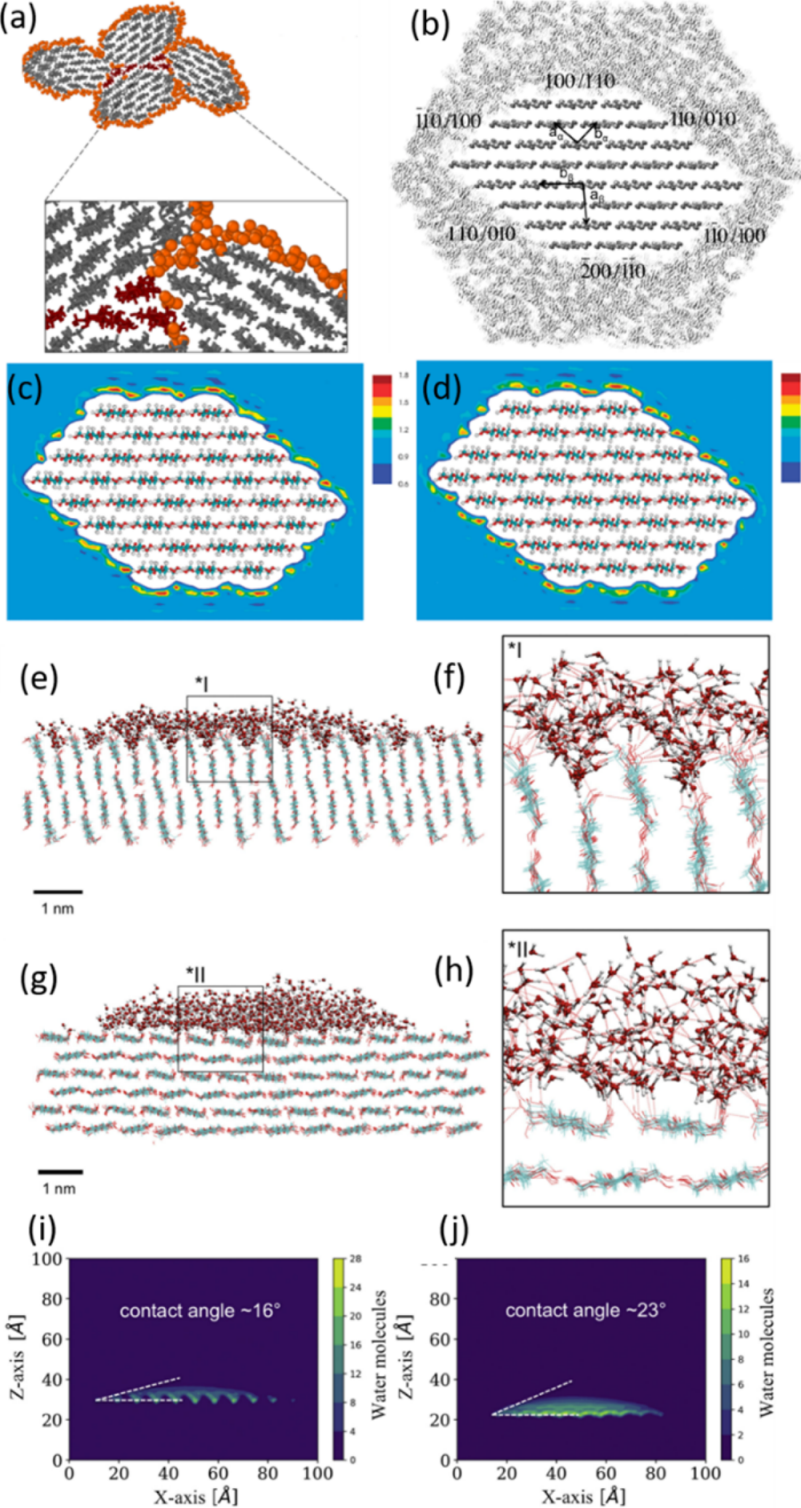
(a) Illustration of water diffusion into a bundle at the cross-section of fibril bundle along its longitudinal axis. Color legend: cellulose in microfibrils (gray), disordered cellulose (dark red), and water (orange). (a) Reproduced from ref (991) under the terms of CC-BY. Copyright 2019 Spring Nature. (b) Solvated water layer on the microfibril cross-section. Both Iβ and Iα are shown next to their corresponding faces with the *c* vector as orthogonal to the plane of the image. (b) Reproduced with permission from ref (992). Copyright 2010 American Chemical Society. Water density of the solvated water at different surfaces of Iβ(c) and Iα (d) with microfibrils represented in the interior. (c,d) Reproduced with permission fromref (992). Copyright 2010 American Chemical Society. (e,f) Side view of water wetting behavior of cellulose Iβ (010) plane. (g,h) Side view of water wetting behavior of cellulose Iβ (100) plane; 2-D density profile of water molecules on Iβ (010) plane (i) and on Iβ (100) plane (j). (e–j) Reproduced with permission from ref (993). Copyright 2019 Elsevier.

The fiber/fibril twist of cellulose is an intriguing and significant observation of morphological changes in model cellulose crystals solvated with explicit water molecules with bulk water properties,^154,155,975−983^ although from an experimental perspective the mechanisms behind its occurrence, and even its existence, are still a debated subject.^152,984−986^ To a certain extent, the twist is speculated to cause the inability of individual microfibrils to coalesce into merged fibrils over long distances. Until now, MD studies have simulated microfibrils with either a rectangular, diamond, or hexagonal cross-section made up of up to 100 cellulose chains and of length between 10 and 50 nm, and a right-handed twist of a few degrees per nanometer of length has been found. Furthermore, the twist has been found to scale inversely with the cross-sectional area yet be independent of fibril length.^154,976,987^ The molecular origins of the twist were suggested to be the result of a combination of intra- or intermolecular hydrogen bonding, electrostatic interactions, van der Waals forces, and the chirality of cellulose chains. Isolated glucan chains in water deviate from the strict 2-fold symmetry assumed in the crystal structure of cellulose rendering them a slight right-handed twist. This is a common feature of all β-1–4 linked oligosaccharides and has been shown to be caused mainly by steric effects with negligeable contribution from intramolecular hydrogen bonding.^988,989^ It is likely that this single-chain twist propagates up within the structural hierarchy giving twisted microfibrils.^155^ This conclusion is reinforced by a study where the microfibril twist is shown to be insensitive to intrachain hydrogen bonding.^990^ Some other studies have also suggested that hydrogen bonding may not influence the twist at all.^981,987^ The microfibril twist in turn progresses to the next hierarchical level of the fibril bundle. However, the twist results in an increase in conformational disorder from the microfibril core toward the surface in the presence of both water and vacuum, causing a longitudinal variation in the bundle structures that prevents cocrystallization and opens up routes for the diffusion of water molecules into said bundles (Figure 20a,b).^991^ The amount of interfibrillar water was found to be roughly half of that of extrafibrillar surface water in MD simulations of microfibril bundles, which correlates well with the experimental analysis of bleached hardwood pulp.^991^

The role of water or solvation for the mechanisms behind fibril twisting is not clear. It has been shown, however, that the physical presence of water significantly mitigates the extent of twisting due to the ability of water to form hydrogen bonds with the crystal surface.^978^ On the other hand, Conley et al. found that water only has a limited effect on the twist unless it disrupts the hydrogen bonds across the glycosidic bonds.^154^ Further, adding an excess of water beyond the first solvation shell does not change the degree of twisting, but this is dependent on the water model used; when using TIP5P water resulted in a noticeable reduction of the twist compared to the TIP3P model, for example.^978^

#### 4.1.3 Effect of Hydration on Cellulose Dynamics

The structure of cellulose at the surface is significantly different from the crystalline bulk structure, as is evidenced by the distribution of the hydroxy groups and torsional angels.^228,952^ These angles are strongly affected due to changes in the hydrogen bonding potential in the presence of water at the cellulose surface. Moreover, the dynamics of surface polymers is distinctly different from those inside the crystal. MD simulations of cross-polarization and magic angle spinning (CP/MAS) solid-state ^13^C nuclear magnetic resonance (NMR) longitudinal relaxation enabled quantitative interpretation of experimental NMR data (explained in detail in section 4.2.2).^994^ The experimental data along with the simulations demonstrated a direct correlation between dynamic and structural heterogeneity at atomic resolution, enabling the understanding of structure–function relationships in controlled hydration conditions. It was found that the doublet at resonance ca. 84 ppm for C4 atoms was due to the nonequivalence of accessible surfaces located on top of different crystallographic planes. Within hydrated cellulose fibril aggregates,^995^ localized cellulose macromolecular dynamics have been deconvoluted into contributions from distinct molecular sources within the aggregated CNFs: (i) the hydroxy groups in the core of CNFs, (ii) the less accessible and accessible surface regions, and (iii) within structurally different surface groups. Upon hydration, this leads to an increased disorder in the hydroxy group conformation at the cellulose surface.^996^ As found in MD simulations, and confirmed with neutron reflectivity experiments, cellulose hydrated to 10% w/w with water is more rigid than dry cellulose as a result of one water molecule forming two or more hydrogen bonds with cellulose to bridge cellulose chains.^996^

#### 4.1.4 Water Structure and Dynamics at Cellulose Surfaces and within Fibril Aggregates

The solvation of cellulose is also of importance in understanding the reactivity of cellulose as it relates to its interfacial properties. Understanding the role of water at cellulosic surfaces and within fibril aggregates will aid the discovery of the underlying process not only for deconstructing cellulose but also for designing functional, chemically modified cellulosic materials for targeted applications.

The solvation structure is determined by asymmetry of crystallographic cellulose surfaces due to the topographical and chemical heterogeneity of each surface. As a result of the heterogeneity resulting form of C–H and O–H rich regions, respectively, the local water density in different hydration layer differs considerably, as illustrated in (Figure 20c,d). Around the O–H rich regions, a high water density leads to a dense first solvation shell, which has been hypothesized to slow down the diffusion of other molecules toward the cellulose surface.^155,992^ Due to the lack of affinity toward the C–H rich regions, water molecules form strong hydrogen bonds with each other, causing small-scale hydrophobic effects.^997^ Along the longitudinal direction of microfibrils, water molecules near the C–H rich surface, i.e., the (2̅00) and (100) planes of the cellulose I_β_ crystal, and the (1̅1̅0) and (110) planes of the cellulose I_α_ crystal, have a higher compressibility than those in other solvation shells (Figure 20b).^992^ Miyamoto et al. focused their simulation specifically on the orientational structuring of water molecules over the (100) crystal plane of cellulose I_β_.^998^ In terms of orientation, they showed that water molecules can approach hydrophilic troughs between cellulose chains and form hydrogen bonds to hydroxy groups to form interchain hydrogen bonding. The hydrophobic strips of cellulose crystals are sufficiently narrower in comparison to the fibril size.^998^

In general, the behavior of the bound water to cellulose surface and fibrils is thermodynamic-driven. With confirmations from quasi-elastic neutron scattering (QENS) studies on deuterated bacteria cellulose on a picosecond scale, O’Neill applied MD simulations to elucidate two unambiguous populations of water (explained in detail in section 4.2.7). As explained in subchapter section 2.4.2.2, “nonfreezing bound” water gradually becomes mobile with increasing temperature in the vicinity of cellulose surface. The second population is “confined water”, which can be attributed to water accumulation in the narrow spaces between microfibrils.^273^ Langan et al. found that fibril aggregation during thermochemical pretreatment with increased temperature up to 160 °C would cause core water expelling among the fibrils. An induced increase of fibril crystalline domain indicating fibril coalescence was also experimentally observed by Kuribayashi et al.^999^ However, water steadily stays between fibrils when the applied temperature is maintained below 76 °C and Chen et al. showed that this population of water is in thermodynamic equilibrium as opposed to being kinetically trapped.^1000^ This means that interfibrillar water lowers the free energy of the bundle and thus acts as an adhesive. On the other hand, interfibrillar water lowers the friction between fibrils facilitating shear deformation,^1001^ contributing to the ductility of cellulose materials.^1002^ Surface charge was also shown to interfere with cellulose–water interactions.^274^ Paajanen et al. presented an informative understanding of the water interactions between fibrils of TOCNF regarding its rheological, aggregation, and disintegration properties using MD simulations.^219^

#### 4.1.5 Wetting and Water Sorption

Cellulose I_β_ crystals with the existence of both hydrophilic surface (e.g., (010), (110), and (11̅0) surface with exposed −OH) and hydrophobic surface (e.g., (100) plane with buried −OH and exclusively −CH moieties) show a featured hygroscopic property. The ab initio studies of interactions between (100) plane and single water molecule were carried out using dispersion corrected DFT method.^1003^ It was concluded that water adsorption on the I_β_ (100) plane is depending on the adsorption size on the plane (Figure 20e,f). Hydrogen bonds could be formed with more accessible CH moieties protruding out of the plane than oxygen atoms of the equatorial hydroxys. Wetting of two cellulose surfaces, (010) and (100), was carried out by MD simulations of contact angle using the native I_β_ model.^993^ In the simulation, a nanodroplet of around 3 nm TIP1P/2005 water was placed near the surfaces. The calculated contact angle in the simulation was around 16° for the (010) plane and around 23° for (100) plane (Figure 20i,j), exhibiting hydrophilic behavior. Similarly, Nawrocki et al. found a contact angle of 25° for TIP3P water with cellulose (100) plane, predicted in their MD simulations using CHARMM force field.^1004^ In a comprehensive study using the CHARMM force field Trentin et al. studied the spreading of water nanodroplets on the different crystallographic planes in several cellulose polymorphs. It was found that all surfaces were hydrophilic (even those commonly termed “hydrophobic”) with static contact angles ranging from <5° (complete wetting) up to 48°. Interestingly, the highest contact angle was found for the “hydrophilic” (001) surface of cellulose I_a_. The differences in wetting were correlated with the conformation and accessibility of surface hydroxymethyl groups.^1005^ Karna et al.^1006^ conducted contact angle simulations between water and cellulose surface at the presence of an external electric field. The application of an electric field with varied direction and magnitude would tune the wettability of cellulose surface, which could be beneficial to the dewatering process of nanocellulose related products. Albeit the wetting behavior is rather similar for both (010) and (100) planes, the solvent molecular organization interacting with each plane is substantially different. It is observed that water penetrating in the interstices between cellulose molecules in the (010) plane forms hydrogen bonds with the exposed surface −OH groups due to high surface roughness. However, on top of the (100) plane, water is inclined to form water structure at the interface along with a characteristic gap on top of the apolar ring (Figure 20g,h).^1007^ Water molecules also prefer to occupy the specific positions, particularly between the glycosidic oxygen atom and the adjacent O-2 and O-3 hydroxy groups.^155^

A phenomenon related to wetting is the formation of capillary bridges. Ogawa et al.^1008^ used MD to study capillary effects during drying of model CNF. As water was gradually removed a meniscus formed, exerting a capillary force large enough to plastically deform the fibrils. At the end of the simulated drying, the two fibrils had partially fused, although some water still remained trapped within the aggregate.

#### 4.1.6 Simulation of the Interactions of Functionalized Cellulose with the Environment

In general, MD simulation studies can be an appropriate tool to understand the role of water in functionalized nanocellulose surface and composite in terms of tailoring interfacial behaviors and tuning compatibilities in composite materials on both bulk and molecular level. As discussed in section 3.3.4, the presence of water at nanocellulose surface detrimentally weakens the interaction between hydrophilic cellulose and hydrophobic polymer matrices, which becomes a problem in cellulose nanocomposite materials. A cellulose I_β_ (11̅0) surface was grafted with caprolactone of different degrees of substitution by Bergenstråhle et al. The hydroxy groups in the native cellulose surfaces interact with other medium such as caprolactone not limited by present water. Surfaces with modified O6H6 and a DS of at least 50% interacted with the surrounding medium mainly through the grafted monomer unit instead of hydroxy groups. However, the adhesion between grafted surfaces and surrounding polymer medium was still prohibited by the presence of water. Increasing the degree of polymerization of the grafts was suggested to diminish the effect of surface water.^994^ Simulations of the interactions between functionalized cellulose surfaces and surrounding medium leading to increased understanding of the self-assembly of nanocellulose would help the development of novel materials. Bouchard et al.^1009^ studied the interaction and adsorption of water and electrolyte on the cellulose nanocrystal pristine surface and its modified surfaces, i.e., carboxylic and sulfate groups, by DFT quantum chemical calculations. The cellulose I_β_ (11̅0) surfaces were more repulsive toward each other, possessing a slightly more negative electrostatic potential map than (110) surfaces. The negative surface functionalities impart a greater CNC surface hydrophilicity, while hydrogen-bonding network within cellulose was restructured in the presence of positive electrolyte ions. Recently, Chen et al. investigated the influence of topochemical modification of cellulose surface on its interaction with water and among the modified nanocellulose particles. It was concluded that acetylation in the C6 position led to hydrophobization of cellulose fibrils and the decrease of the work of adhesion between the acetylated model surface and water. Most interestingly, the acetylation was found to greatly increase the dispersibility of nanocellulose.^376^

### 4.2 Experimental Methods to Uncover Nanocellulose–Water Interactions

This section introduces the key analytical techniques that are currently used to investigate cellulose–water interactions and gives a few selected examples of published works from each that specifically measure the effect of water on nanocellulose-based materials. The overview of analytical methods is not exhaustive and is limited to the key analytical techniques that are currently used to investigate cellulose–water interactions. For each of the discussed techniques, we provide selected examples of works that specifically measure the effect of water on nanocellulose-based materials.

**Figure 21:**
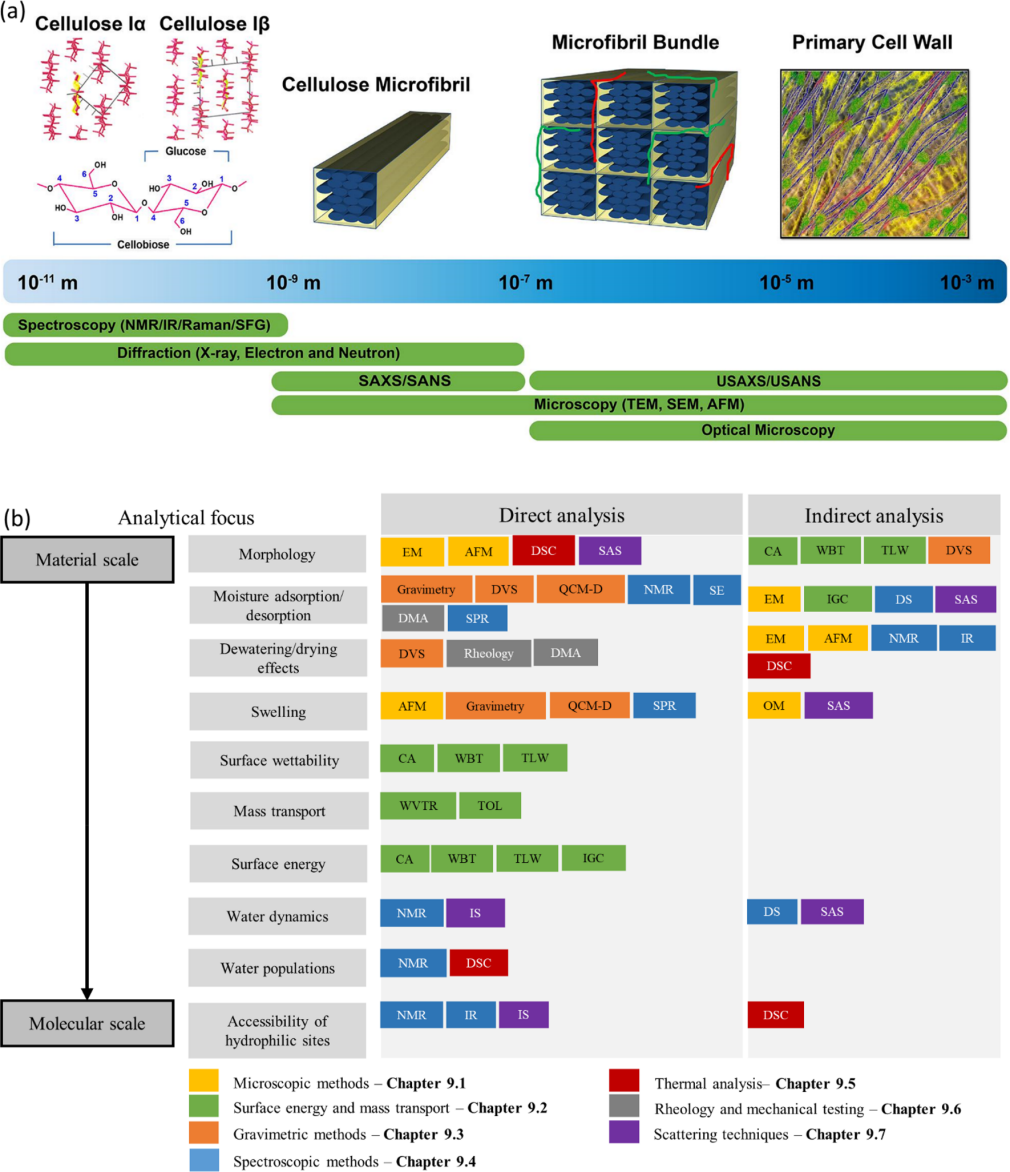
(a) The scale of hierarchical structure of the primary cell wall of plants with respect to cellulose and tools enabling characterization of cellulose in each level of magnitude.^1010−1013^ Adapted from ref (1011) under the terms of CC-BY. Copyright 2019 Frontiers. Reproduced with permission from ref (1010). Copyright 2002 American Chemical Society. Reproduced with permission from ref (1012). Copyright Elsevier. Reproduced with permission from ref (1013). Copyright 2015 Elsevier. (b) Overview of experimental techniques for direct and indirect analysis of nanocellulose–water interactions: AFM, atomic force microscopy; CA, contact angle; DMA, dynamic mechanical analysis; DS, dielectric spectroscopy; DSC, differential scanning calorimetry; DVS, dynamic vapor sorption analysis; EM, electron microscopy; IGC, inverse gas chromatography; IR, infrared spectroscopy; IS, inelastic scattering; NMR, nuclear magnetic resonance spectroscopy; OM, optical microscopy; QCM-D, quartz crystal microbalance with dissipation monitoring; SAS, small angle scattering; SE, spectroscopic ellipsometry; SPR, surface plasmon resonance spectroscopy; TLW, thin-layer wicking; TOL, tritium oxide labeling; WBT, Washburn techniques; WVTR, water vapor transport rate.

The wide variety of analytical techniques and protocols developed to probe nanocellulose–water interactions reflects the complexity of these interactions and the need to examine them from different perspectives. In paper making processes, well-established technical process-orientated standard methods such as freeness value (Schopper Riegler (°SR) test), water retention value, and other online paper web moisture measurements reflect cellulose–water interaction including runnability and processability in a macroscale level. However, most of these techniques are not directly applicable for studies of nanocellulose materials, unless further development takes place. This section introduces the key analytical techniques that are currently used to investigate cellulose–water interactions and gives a few examples of published works from each that specifically measure the effects of water on nanocellulose-based materials. These techniques and their analytical focus are summarized in Figure 21a,b to guide the reader to the appropriate analytical technique for their research needs.

#### 4.2.1 Microscopic Methods

Microscopy, including electron microscopy (EM) and optical microscopy can be used as an indirect analytical tool to shed light on water related phenomena in nanocelluloses, mostly with respect to drying and, in some cases, also to swelling processes.

##### 4.2.1.1 Electron Microscopy (EM)

Conventional scanning electron microscopy (SEM) and transmission electron microscopy (TEM) have been used to observe the morphology of CNCs and CNFs, to determine their dimensions,^297,759,1014,1015^ and to study the impact of different treatments and drying conditions on nanocellulose structures.^299,444,525,760,900,1016^ While an excellent tool to study nanocellulose, EM measurements are carried out in vacuum, and so sample drying (e.g., ambient drying, freeze-drying, heated drying) has an important and sometimes irreversible effect on the morphology of the structures,^1017^ demanding careful image interpretation. Moreover, SEM imaging often involves sputtering of the samples with thin conductive layers which can distort sample features, particularly at the nanoscale. In contrast to conventional SEM, environmental SEM (ESEM) does not require as high a vacuum, thus enabling measurements to be carried out with some residual moisture and/or relative humidity.^1018^ However, controlling the imaging conditions is difficult, and the limited resolution of the microscope may explain the restraint in using this technique to explore nanocellulose–water interactions. Nevertheless, a few examples highlighting the analytical value of SEM and ESEM are briefly described below.

**Figure 22:**
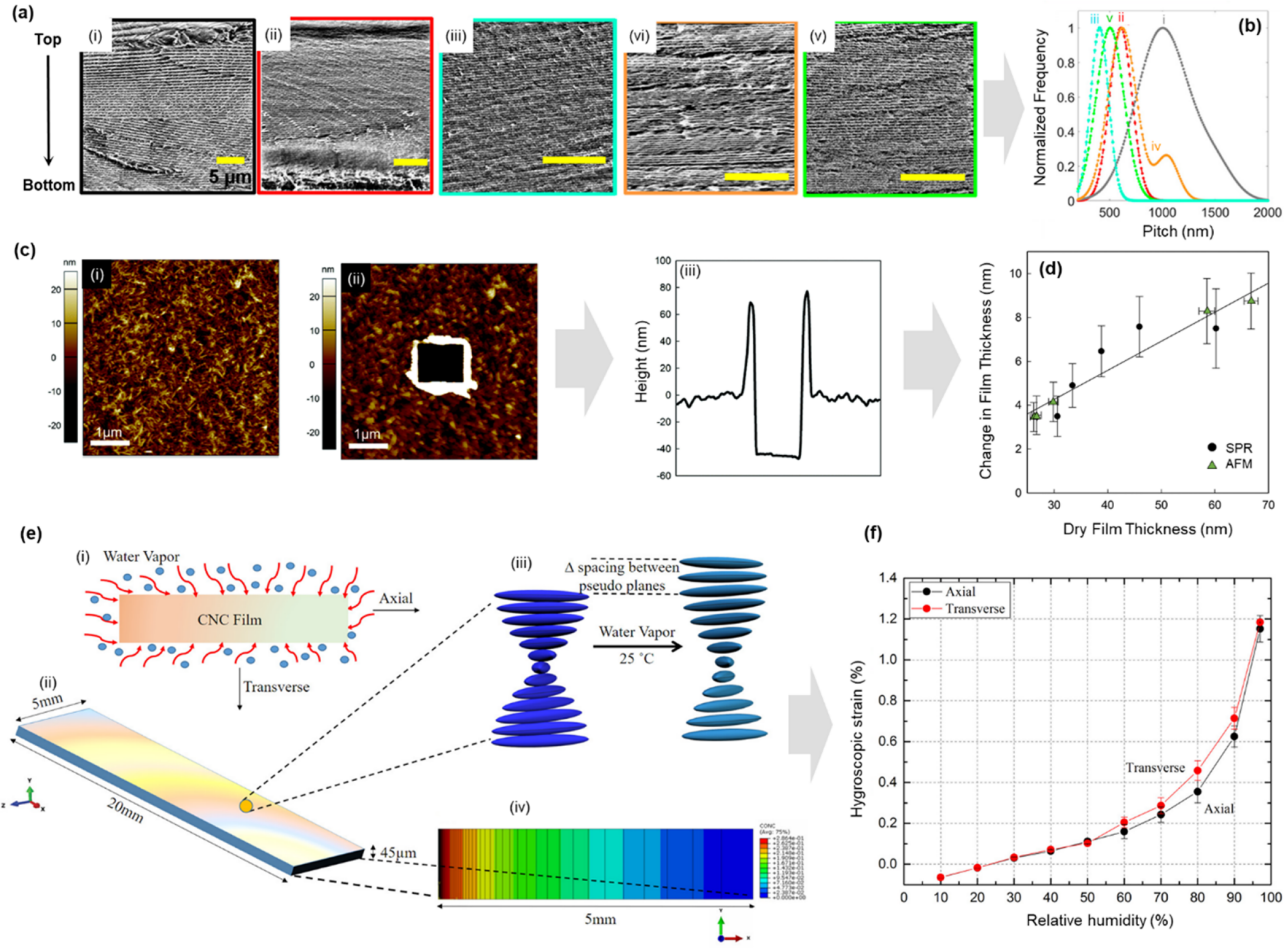
Nanocellulose–water interactions revealed by microscopic techniques: (a) SEM images of the cross-section of pristine CNCs (i) fast-dried, (ii) slow-dried, (iii) slowest-dried, and MePh3P-modified CNCs (iv) fast-dried, and (v) slow-dried. Scale bars = 5 μm.^1016^ (b) Nominal pitch distributions measured from SEM images of films (i–v).^1016^ (a,b) Adapted with permission from ref (1016). Copyright 2017 American Chemical Society. (c) AFM height image of a dry CNC film (i) which was scratched (ii) for cross-section height analysis (iii) to determine the film thickness.^284^ (d) Change in CNC film thickness in water determined by AFM and SPR (solid line for eye guidance).^284^ (c,d) Adapted with permission fromref (284). Copyright 2009 The Royal Society of Chemistry. (e) Scheme representing CNC film moisture sorption. (i) moisture diffusion into the film, (ii) 3D scheme of the CNC film for moisture diffusion analysis by cross-polarized microscopy, (iii) interspace between the CNCs increasing upon water penetration, and (iv) simulation contour of moisture diffusion after 15 h.^331^ (f) Hygroscopic strain, obtained by digital image correlation, as a function of the RH for self-organized CNC films for axial (black) and transverse (red) directions.^331^ (e,f) Adapted with permission from ref (331). Copyright 2017 American Chemical Society.

Natarajan et al. used SEM to quantitatively investigate the morphology and chiral nematic structure of sulfated CNC films, whereby the CNCs were neutralized with different cations and dried under controlled conditions.^1016^ SEM images revealed that faster evaporation rates caused a disruption in the chiral nematic liquid crystal ordering of the film due to vitrification. The images were also used to quantify the nominal pitch (i.e., the helical modulation length) distribution from fractured film cross-sections using a novel image processing method (Figure 22a,b). The calculated pitch distributions were in good agreement with UV–vis–NIR total reflectance spectra. While Gaussian fitting the pitch distribution from SEM helped to obtain peak positions and peak widths, the measured pitch and standard deviation values showed an identical dependence on the evaporation rate of water. For example, greater sample uniformity upon slow evaporation gave a much smaller pitch (i.e., a tighter chiral nematic twist) and smaller standard deviation. Moreover, the SEM was sensitive to the multidomain structures of the fast-dried films. In addition to pitch variations related to different water evaporation rates, a gradient in pitch was observed in going from the top to the bottom of each film down the cross-section, which was larger for films and dried more quickly. Finally, the SEM images showed that the shape of moisture inclusions and water confinement in the CNC films varied with the different counterions investigated.^1016^

Lewis et al. used SEM to investigate the morphology of freeze-dried hydrogels formed by cyclic freeze–thawing of CNC suspensions^900^ and confirmed the formation of an interconnected, porous cellulosic sheet network. Ansari et al. used fractography studies in SEM images of nanopapers from phenyl glycidyl ether oligomer-grafted CNFs to relate property differences to structural changes.^444^ Moreover, differences in swelling could be observed from the SEM micrographs. Rapid dewatering of nanocellulose suspensions was also a key point in the study of Österberg et al., who used hot pressing to prepare robust, solvent-resistant, CNF films. SEM revealed a significantly increased film density after extensive pressing (2 h).^760^ In contrast to SEM studies of dried nanocellulose structures, ESEM enabled Gelin et al. to obtain information about the nanostructure of BC with respect to water content at different relative humidity values.^1018^ ESEM images taken at 77% relative humidity showed that the inner side of the BC gel tubes contained between 10 and 40% water.

Even though TEM has been widely used to investigate the size and shape of nanocelluloses either frozen (cryo-TEM) or dried from water and other solvents, the technique has not been directly used to investigate nanocellulose–water interactions. This is likely due to sample preparation and imaging limitations despite the nanometer spatial resolution. Nevertheless, TEM has been used to corroborate the results obtained from other methods. Gelin et al. coupled the information from freeze-fractured samples imaged by TEM with the magnitude of diffusion coefficients extracted from dielectric spectroscopy, supporting the theory that free (or bulk-like) water present in BC gels is confined as “lakes” rather than forming a continuous phase throughout the gel structure.^1018^

##### 4.2.1.2 Atomic Force Microscopy (AFM)

AFM, a scanning probe microscopy technique, is another visualization tool to investigate nanocellulose. Its versatility is based on the different possible measurement modes and experimental setups including imaging modes (e.g., contact mode or alternating current/tapping mode), and surface force measurement modes (e.g., to determine precontact attractive/repulsive/steric, adhesive, and frictional forces, as well as mechanical properties), e.g., AFM measurements can be done in ambient conditions, under controlled temperature/humidity, or samples can be fully submerged in liquid all while ensuring nanoscale and piconewton resolution. For example, Reid et al., studied the swelling behavior of CNC thin films in the presence of water, by imagining the height profiles of scratches made into the film before and after exposure to water (Figure 22c,d).^284^ Ahola et al. used colloidal probe AFM to measure forces between a cellulose sphere, glued to a tipless cantilever, and a CNF surface.^1019,1020^ They studied the effect of nanofibril charge density, ionic strength, and pH on the swelling and surface interactions of CNF model films; AFM and QCM-D were used in conjunction to infer that both steric and electrostatic forces were present in water and that steric forces dominated between cellulose surfaces under low pH and high ionic strength conditions.^1019,1020^

##### 4.2.1.3 Optical Microscopy

The limited resolution of optical (or light) microscopy compared to other techniques has restricted its application as a visualization tool for nanomaterial characterization.^299^ Nevertheless, there are examples of indirectly using optical microscopy to investigate nanocellulose–water interactions. For example, Shrestha et al. used a contrast enhanced microscopy digital image correlation technique to characterize the dimensional changes induced during hygroscopic swelling of self-assembled and shear-oriented CNC films (Figure 22e,f).^331^ The authors applied the distinct microstructure and birefringence of CNC films to explore the in-plane hygroscopic swelling by correlating cross-polarized microscopy images at relative humidity levels ranging from 0 to 97%. The in-plane coefficient of hygroscopic swelling of the CNC films was thereby determined by optically tracking humidity-driven strain fields in a contact-free manner.

#### 4.2.2 Surface Energy and Mass Transport Methods

As discussed earlier, surface energy together with the surface roughness, determines the wettability of a surface by a liquid. Among the most common ways to characterize the surface energy of cellulosic materials are inverse gas chromatography (IGC), thin-layer wicking (TLW), and different types of contact angle measurements,^479^ which will be outlined in the following section. Moreover, as elucidated in sections 2.4.1.2 and 2.4.2.4, surface energy and wettability are intimately linked with mass transfer, the phenomenon of a net movement of species from one location to another. Here, we will only consider methods to characterize the mass transfer of water in cellulosic materials and make a distinction between the transfer of liquid water and water vapor. The mass transfer of liquid water can be characterized using TLW experiments using the Lucas-Washburn eq (eq 1), introduced in section 2.4.2.4. Moreover, the water mass transfer across, for example, cellulose nanopapers can be measured gravimetrically by different flow tests (gravitational or pressurized)^1021^ or even by diffusion cells equipped with tritium oxide labeling and radioactivity monitoring.^525^ In the case of water vapor transfer over cellulosic films, the vast majority of literature report either the water vapor transmission rate (WVTR) or its close variant water vapor permeability (WVP) using a gravimetric method described in the following.

##### 4.2.2.1 Contact Angle

**Figure 23:**
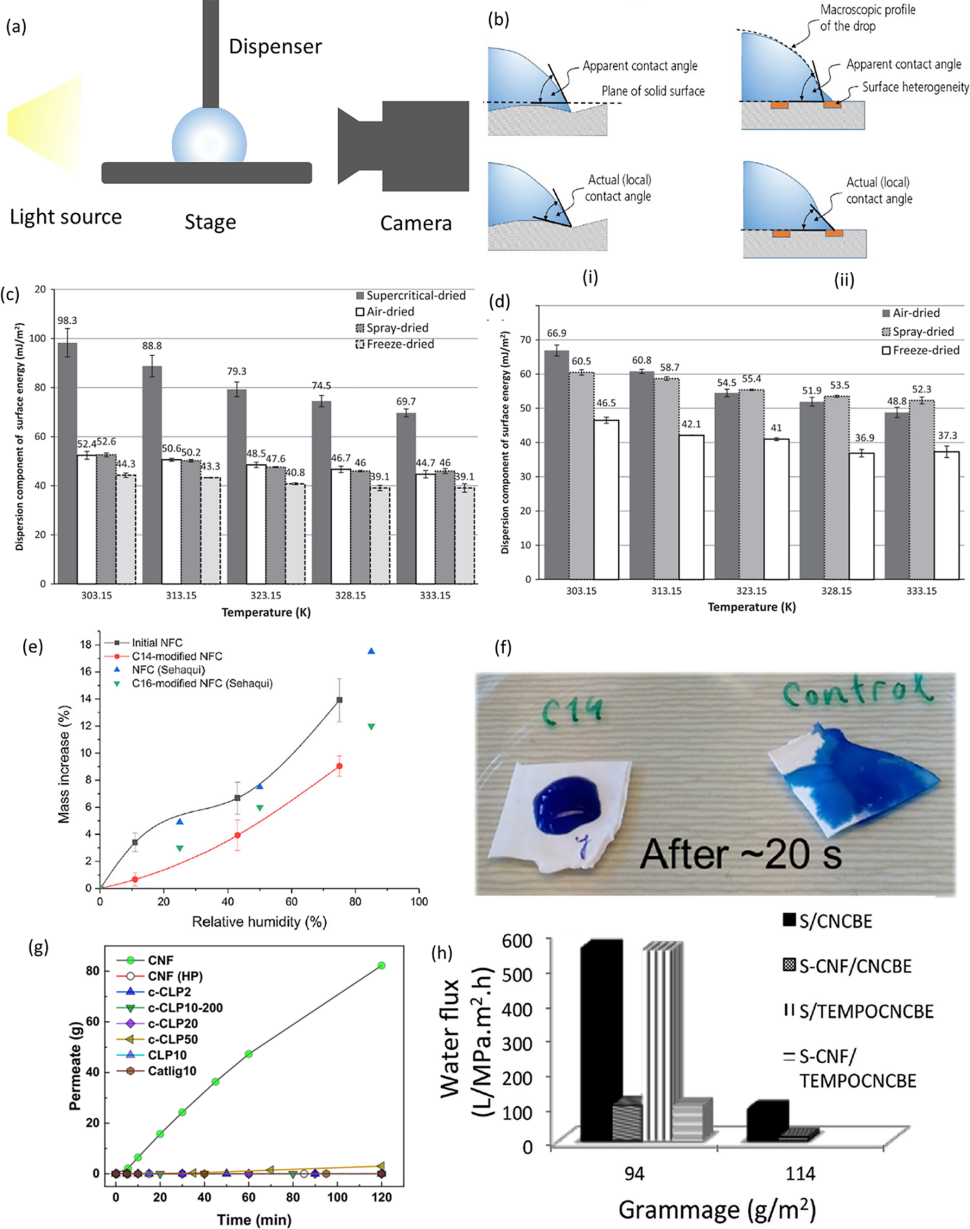
Application of methods to measure surface energy and mass transport in investigating nanocellulose–water interactions. (a) The operating principle of sessile drop goniometry.^1022^ (b) A liquid droplet on a nonideal solid surface: (i) apparent and real contact angles on a rough surface, (ii) apparent and real contact angles on a heterogeneous surface.^1029^ (a,b) Adapted with permission from ref (1029). Copyright 2018 Springer Nature. (c,d) Comparison of the dispersion component of surface energies of CNFs dried with different methods: (c) CNFs and (d) CNCs.^255^ (c,d) Adapted with permission from ref (255). Copyright 2013 Elsevier. (e,f) Water vapor uptake studies to investigate the effect of surface modification: (e) Water vapor uptake isotherms of the initial and C14-modified CNF films (black squares and red circles, respectively), plotted together with data from comparable CNF samples. (f) The qualitative difference in wetting behavior of the two film types 20 s after being exposed to methylene blue dyed water droplets. The hydrophobic C14-modified film is on the left, the untreated control film is on the right.^297^ (e,f) Adapted with permission from ref (297). Copyright 2017 Springer Nature. (g) Permeation of water under ambient pressure and at room temperature for unmodified CNF nanopapers and modified CNF nanopapers by hot pressing and lignin content. Hot pressing and lignin reduce the permeation of water in ambient conditions, significantly.^1021^ (g) Adapted under the terms of CC-BY from ref (1021). Copyright 2019 American Chemical Society. (h) Water flux of fabricated support layers for enhanced adsorption of metal ions with and without sludge microfibers/cellulose nanofibers (CNFSL). Layered fabricated membranes indicate a decrease in water flux but in situ TEMPO oxidation has no significant effect on water flux.^1030^ (h) Adapted under the terms of CC-BY from ref (1030). Copyright 2017 The Royal Society of Chemistry.

Among the most common ways to analyze the contact angle is sessile drop goniometry, where a high-speed camera captures a video of a surface wetted by a droplet of liquid. In the simplest setup, this is done on a horizontal surface (Figure 23a),^1022^ although other possibilities also exist.^1023−1026^ The captured images are then analyzed by computer by numerically fitting a function to the images,^1027,1028^ yielding an estimate for the contact angle at each moment of time. The time-dependency of the contact angle varies a lot: for example, a water droplet will wet a rough, high surface energy solid almost instantly, whereas hydrophobic surfaces will resist wetting by the droplet and the droplet will retain its shape until it slowly evaporates.^509^ It is therefore common to record not just initial (or static) contact angle but also to observe how stable the droplet is over time. One of the limitations of static contact angle measurements is that they are not always reproducible, but one way to overcome this is to record advancing and receding contact angles, which tend to be more reliable.^1022^

A dynamic extension of the static sessile drop technique allows the detection of advancing and receding contact angles by increasing and decreasing the volume of the deposited droplet (Figure 23b).^1022^ The difference between these angles is referred to as contact angle hysteresis,^1026^ which provides an indication of surface roughness, possible surface contaminations, etc.^1026,1031^ In the case of cellulosic materials, contact angle hysteresis can also infer tendency of the material to absorb moisture.^1032^ The more pronounced the hysteresis, the more firmly the droplet is attached to the surface and the more wettable the surface is by the liquid.^1022,1026^

To determine surface energies by contact angle measurements, multiple probe liquids of varying polarities are used, including ethylene glycol, formamide, toluene, glycerol, and diiodomethane.^1033,1034^ Surface energies can then be determined using the Fowkes method, where the surface energy is divided into the dispersive and the polar component, combined by a geometric mean approach.^1035^

As contact angle measurements are sensitive to surface roughness, they are at their most accurate on smooth and rigid surfaces. Nanocellulosic surfaces are not considered smooth or rigid, and as such this limitation should be kept in mind even though contact angle analysis, especially by the static sessile drop technique, is commonplace within the cellulosic materials research literature. Spin-coating and multilayer Langmuir–Blodgett/Schaefer deposition of nanocellulose films can be used to obtain smoother films than those from solvent casting, but the degree of aggregation of nanocellulose in liquid prior to film preparation can also affect surface roughness and porosity; AFM is a good method to quantify these film properties. Owing to its operational simplicity, contact angle analysis has most often been used in a semiquantitative fashion, where the wettability by a static droplet of liquid water on a series of samples is compared.^253,509,755^ At the very least, significant differences in wettability can be detected by this simple approach, allowing for a quick check of, for example, the success of a surface hydrophobization treatment.

The multiple probe liquid approach has been applied to calculate the surface energies of cellulose nanopapers,^784^ CNC thin films,^1032^ ultrathin films of amorphous cellulose,^245^ etc. Moreover, through hysteresis analysis, cellulosic surfaces have been shown to be sensitive to moisture uptake; some water molecules inevitably remain on the surface upon retraction of the droplet, further enhancing their wettability by water.^1032^ It should also be noted that cellulose readily swells and curls under water during contact angle measurements, and these dynamic changes caused by wetting and drying may significantly alter the perceived surface energies of cellulosic surfaces.^232,245,253,254^ These effects complicate the interpretation of contact angle values,^1036^ so an alternative strategy employing the Washburn technique may be considered.

##### 4.2.2.2 Washburn Technique and Thin-Layer Wicking

Closely connected with contact angle analysis, the Washburn technique (or the capillary rise technique) is based on the spontaneous flow of a probe liquid through a column filled with the porous material to be analyzed.^1037^ It enables the simultaneous determination of the pore radius, contact angle, and surface energies of porous samples. The uptake of the probe liquid can be detected either gravimetrically or by observing the change in the height of the liquid layer.^1038^ Very similar in operational principle, TLW is an example of dynamic wetting that is based on liquid penetration into a porous solid.^254,1039^ All of these techniques rely on a phenomenon called wicking, i.e., the spontaneous spreading of a liquid into a porous material caused by the pressure difference resulting from the spherical meniscus of the wetting liquid.^254^ To determine the surface energy components of a solid by TLW, a multiple liquid approach is used, much in the same way as in the contact angle measurements.^254^ At least three probe liquids of known dispersive and polar components are required, and their contact angle, vapor adsorption isotherm, or the penetration rate should be measured.^1040^ Operating under a set of assumptions,^1040^ a modified form of the Lucas–Washburn equation relates the rate of penetration to the surface energy components:^1041^
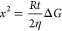
2where *x* is the distance traveled in time *t*, η is the liquid viscosity, *R* is the effective capillary radius, and Δ*G* is the Gibbs energy change accompanying liquid penetration into the solid. Δ*G* may then be converted to the surface energy by different means, depending on the type of the liquid–solid system in question.^1041^

TLW has been applied to measure the surface energy of microcrystalline cellulose (MCC)^239^ and cotton fabrics,^1042^ but its role in the cellulose materials science has remained much more peripheral than the omnipresent contact angle measurements. The same applies to other wicking-based techniques, including capillary intrusion that has also been utilized to measure the surface energy of MCC.^114^ As we noted in the context of contact angle analysis, the adsorption of water vapor on nanocellulose surfaces has an influence on the wicking behavior of water.^254^ This is a relevant concern for an extremely hygroscopic material like cellulose that is in practice covered by a layer of adsorbed water molecules at all times.^1043^

##### 4.2.2.3 Inverse Gas Chromatography (IGC)

In contrast to contact angle and wicking-based experiments that are sensitive to sample roughness and porosity, IGC can be used to characterize the surface energies of particles. The basic principle of IGC is similar to gas chromatography (GC) in the sense that there is a gaseous substance passing through a column of a stationary phase. In IGC, the sample to be investigated acts as the stationary phase, whereas in traditional GC, the sample would be carried by an eluent gas.^1044^

IGC analysis can be run at different relative humidity and temperature and can yield information on, for example, the specific surface area, degree of crystallinity, solubility, and thermodynamic interaction parameters, glass transition temperatures, and the dispersive and polar components of surface energy.^1044,1045^ IGC surface energy analysis is run at infinite-dilution conditions utilizing a number of different probe molecules, usually a series of alkanes (e.g., hexane, heptane, octane, etc.) in combination with polar probes (e.g., dichloromethane, acetone, acetonitrile, and ethyl acetate).^1045,1046^ The retention times and surface coverages are then recorded and used to calculate the dispersive component, polar component, and total surface energies. This requires the assumption that all probe liquid retention is due to adsorption solely to the sample, and that the interactions between the probe molecules are negligible.^1047,1048^

For cellulose and nanomaterials made thereof, IGC has been used to assess the surface energies of wood-based and cotton fibers,^106,237,1049^ MCC,^114,1050^ CNFs,^236,242,255^ and CNCs,^534^ nanostructured cellulose II particles,^243^ and even amorphous cellulose,^241^ although the reliability of the analysis of amorphous samples may be affected by probe diffusion into the material.^1050^

In the context of cellulose–water interactions, the possibility to determine the surface energy components at different relative humidity values offers insights into the rearrangements that take place when cellulose is subjected to moisture (Figure 23c,d). Differences in cellulose reactivity have been reported, depending on the utilized drying method, and it is likely that the measured surface energies are also sensitive to such effects, as evidenced by XPS and contact angle measurements.^232^

##### 4.2.2.4 Gas Permeability Studies

Several standardized tests exist for the permeability of various gases through a number of polymeric materials. Here, we will only consider water vapor permeability measurements of cellulosic films, although the permeability of other gases through cellulose nanopapers also increases at relative humidity values of approximately 70–80%.^745,1051^ The simplest way to assess the water vapor permeability of cellulose nanopapers is gravimetrically, by the so-called wet cup method,^1052^ although corrections have been proposed to the method.^1053,1054^ In the common wet cup setup, a sealable cup is filled with a constant volume of distilled water, covered tightly by the tested material, and placed on the top of a balance that records weight loss as a function of evaporation time.^253,1055,1056^ Relative humidity inside the cup is assumed to be 100%, and the relative humidity outside the cup can be controlled by the use of saturated salt solutions, for example.^253,1057^ An analogous but reversed setup (i.e., the dry cup method) also exists, where a desiccant material is used on one side of the material (Figure 23e,f).^1054^

This diffusion-driven process is sensitive to changes in temperature and relative humidity (and thereby partial pressure) on both sides of the nanopaper,^291^ and meaningful comparisons of mass transfer rates can only be made when these factors are kept constant. Obviously, properties of the nanopaper such as thickness,^292^ crystallinity,^293^ porosity, and pore size as well as pore structure,^295^ and hydrophilicity,^294^ also influence mass transfer rate, and it is usually necessary to characterize these properties in combination with the measured WVTR values to truly understand the material behavior. WVTR can be calculated from the steady-state region of the mass loss:^298^
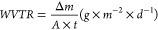
3where Δ*m* is the change in sample mass, *A* is the evaporation area, and *t* is time.

Obviously, the underlying phenomena in WVTR are related to mass transport as elaborated in section 2.4.2.4. As the foremost application potential of cellulose nanopapers is as barrier films, the focus research in the field has largely been on improving their barrier properties in humid conditions,^745,755,767,768^ rather than maintaining efficient mass transfer across the nanopaper.^297^ As the transport of gases and liquids proceed differently, and it is easier to block the flow of liquid water with its high cohesion and surface tension than that of individual water molecules.

##### 4.2.2.5 Liquid Permeability Studies

In the nanocellulose literature, liquid water mass transfer values are often omitted from the experimental setup, even if different iterations of contact angles and WVTRs are frequently reported. The ease of dewatering during cellulose nanopaper preparation has received far more attention^784,1051,1058,1059^ than the permeability of liquid water through pre-existing cellulose nanopapers, although some accounts do exist (Figure 23g,h).^1021,1030^

For relatively water-permeable nanocellulose films, different flow tests have been applied which rely on either atmospheric pressure or an added pressure gradient for mass transfer,^1021,1060^ and the permeated water is recorded as either volume or mass. Despite their tendency to swell and lose their gas barrier function in water, cellulose nanopapers can efficiently slow down the transport of liquid water. To characterize the mass transfer that is too slow to be meaningfully determined by gravimetric or volumetric methods (due to evaporation), an alternative strategy of employing tritium oxide labeling may be considered.^525,1061^ In such a setup, a dual chamber is divided by the tested film and filled with deionized water simultaneously on both sides to avoid any pressure differences. Then, a small quantity of tritiated water (tritium oxide) is added on the donor side of the chamber, and the radioactivity of water on the acceptor side is monitored at regular time intervals (the measurement is carried out under constant stirring).^525,1061^ This technique is more cumbersome than gravimetric flow tests and requires special facilities suitable for working with radioisotopes.

#### 4.2.3 Gravimetric Methods

Several gravimetric methods exist for the analysis of water retention and water uptake capacity of pulp and paper materials,^332,1062−1067^ and many of them have been applied to the analysis of nanocelluloses as well. In this section, we will move from more crude techniques such as water retention value (WRV) and Cobb tests to those of higher sensitivity such as DVS and QCM-D.

##### 4.2.3.1 Water Retention Value and Cobb Test

**Figure 24:**
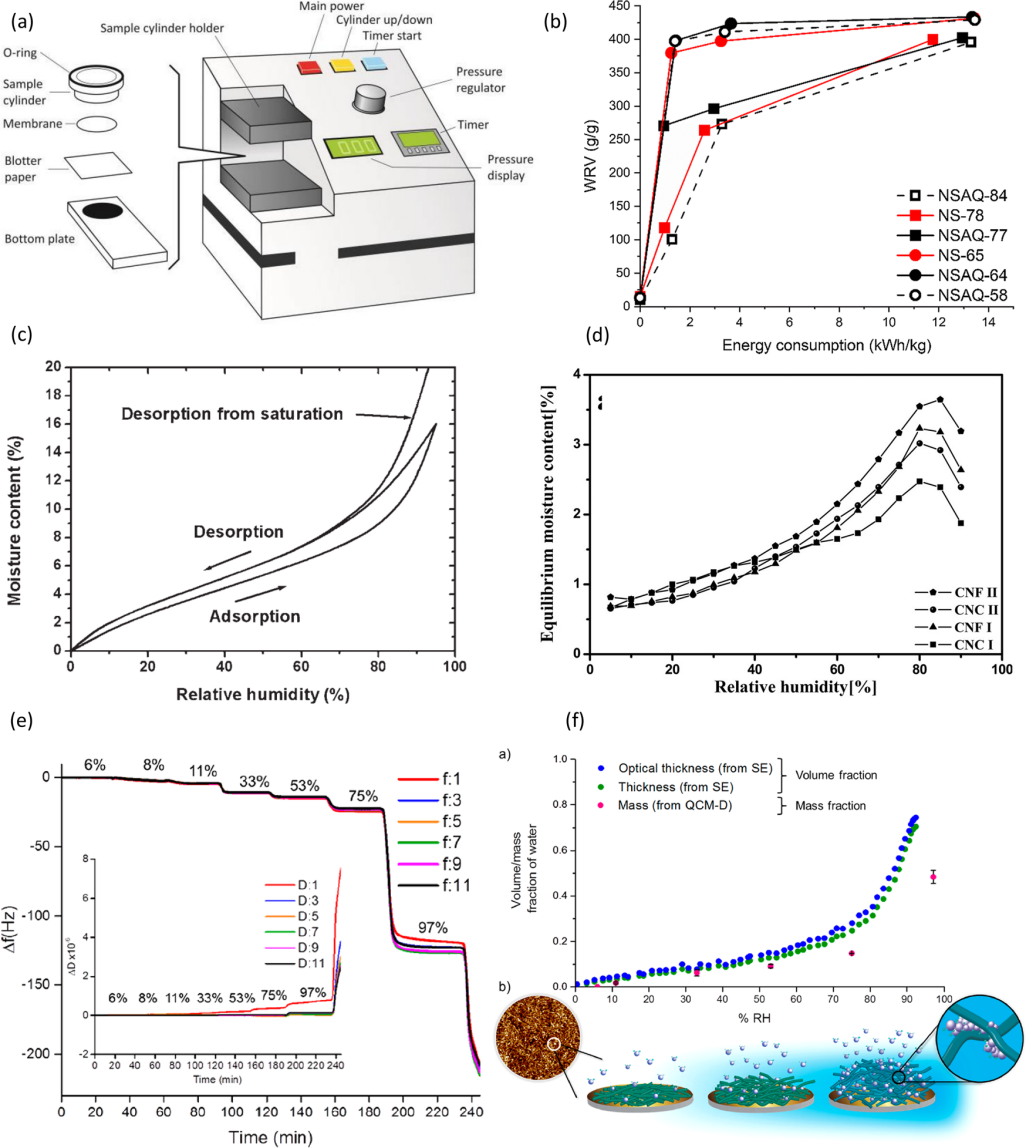
Application of gravimetric methods in the investigation of nanocellulose–water interactions: (a) An example of a gravimetric water retention analysis apparatus.^1068^ (a) Adapted under the terms of CC-BY from ref (1068). Copyright 2014 North Carolina State University. (b) The water retention values (g/g) of the CNFs produced from semichemical pulps obtained from neutral sulfite (NS) pulping (with or without the addition of anthraquinone (AQ)), as a function of specific energy consumption (kWh/kg) in the process.^1069^ (b) Adapted under the terms of CC-BY from ref (1069). Copyright 2020 Springer Nature. (c) Typical behavior exhibited by a lignocellulosic material by DVS, when desorbing moisture from a fully water-saturated state and when desorbing moisture from a nonwater-saturated state. Hysteresis between the adsorption and desorption isotherm is shown.^1066^ (c) Adapted with permission from ref (1066). Copyright 2009 John Wiley and Sons. (d) Sorption hysteresis of four nanocellulose samples (both CNC and CNF), measured by DVS.^215^ (d) Adapted under the terms of CC-BY from ref (215). Copyright 2017 Springer Nature. (e) Change in frequency (Δ*f*) and dissipation (Δ*D*) as a function of time in stepwise increasing relative humidity (% RH) as detected by QCM-D water vapor adsorption measurements for TEMPO-oxidized CNF (TOCNF) thin films (different overtones are indicated with colors).^282^ (f) (i) Mass (pink), optical thickness (blue), and thickness (green) fractions of water in TOCNF thin films due to water vapor uptake as a function of relative humidity. Thickness and optical thickness fractions of water are deduced from data collected by SE. Mass fractions of water are based on QCM-D measurements; (ii) schematic illustration of the water vapor uptake of a TOCNF thin film in dry air (RH < 10%), in humid air (10–75% RH) and at high humidity levels (RH > 75%).^282^ (e,f) Adapted with permission from ref (282). Copyright 2017 American Chemical Society.

The WRV^1063^ and the Cobb test^1064^ provide slightly different perspectives on the water retention and water uptake capacity of cellulosic materials. As the swelling ability of cellulosic materials depends on the pH and ionic strength of the solvent, and the charge group and counterion of the nanocellulose, these should be kept constant when running these types of analyses in order to yield comparable data.^134,285^ WRV can be used for the quantification of the liquid water that remains in a saturated cellulose sample after a controlled centrifugation cycle (i.e., a capillary swelling test) (Figure 24a,b), whereas in a Cobb test, a preconditioned cellulose sample is wet with a constant volume of water for a given time, followed by a rapid removal of any excess water.

The quantity of retained (WRV) or absorbed (Cobb test) water is then determined through gravimetric analysis. For WRV, the quantity of retained water is expressed as g of water per g of cellulose and calculated by eq 4:^1063^
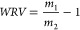
4where *m*_1_ is the mass of the centrifuged wet test pad and *m*_2_ is the mass of the test pad after oven drying.

The Cobb test result, or absorptiveness, is expressed as g of water per m^2^ cellulose, and calculated by eq 5:^1064^
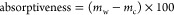
5where *m*_w_ is the mass of the wet sample, *m*_C_ is the mass of the conditioned sample, and the multiplication factor 100 is used for the standard specimen area of 100 cm^2^.

Both WRV and the Cobb test have been applied with slight modifications for the analysis of microfibrillated cellulose and CNFs,^1058,1068−1074^ whereas WRV has been used to characterize BC.^1075^ However, the characterization of CNCs using these means it is not advisible due to the risk of film disintegration and/or material loss during the experiments.

##### 4.2.3.2 Dynamic Vapor Sorption Analysis (DVS)

DVS has been widely applied to study the adsorption–desorption behavior of gases on solids. In relation to cellulose–water interactions, it has been utilized extensively to study wood,^1076^ various plant fibers,^1066^ MCC,^1065^ CNFs,^1077^ and CNCs.^215^ In its most simple application, DVS can be used to quantify how much a given mass of material “takes up” water vapor at a given relative humidity and temperature (i.e., its equilibrium moisture content) (Figure 24c,d). The measuring device is essentially a high-precision mass balance that measures the weight of the sample at different relative humidity values (or partial pressures of gases other than water vapor) and temperatures. DVS can also be used to study the kinetics of the water uptake.^215^ The rate of adsorption and desorption of water from nanocellulose structures has been associated with the accessibility of cellulose hydroxy groups, another parameter frequently researched using DVS, often in conjunction with deuterium exchange.^205,1078^ Moreover, cellulose hydroxy accessibility has been found to correlate well with the equilibrium moisture content of the material.^106,205^

When the relative humidity is increased and decreased gradually, a sigmoidal adsorption–desorption isotherm is obtained, the shape of which provides indirect information on material properties such as pore size, cumulation mechanism of the vapor (i.e., monolayer or multilayer formation), and capillary condensation.^215,1066,1079^ It should also be noted that the adsorption and desorption isotherms are different; this phenomenon is referred to as hysteresis, reminiscent of the difference between the advancing and receding contact angles. Part of the reason may lie in that the mechanisms of adsorption and desorption are different (sorption on an initially dry surface as opposed to evaporation from a capillary meniscus). Another proposed explanation is that the adsorption and desorption take place from different material states (a dry/collapsed vs a swollen one).^1065,1066^ The sorption hysteresis of freeze-dried CNFs has been reported to be larger than that of CNCs, which is interpreted to be the result of the higher portion of disordered cellulose in CNFs.^215^ DVS data has been compared with that extracted from techniques such as WRV^1078^ and thermoporometry,^1065^ during both the initial building of a hydrated layer and after all of the material pores have been filled with water (i.e., saturation). DVS and thermoporometry data from macroscopic cellulose fibers by Driemeier et al. suggested that both the hysteresis behavior and wet porosity are the result of the suprafibrillar organization of cellulosic material^1065^ and that there may be an “ink bottle” type of effect that would further explain the difficulty of water removal upon desorption.^1066^

The topic of sample preparation is crucial, especially when applied to nanocellulose. Usually, DVS experiments are carried out for predried samples that are wetted during the measurement, although it is in principle possible to dry the sample in situ while monitoring the weight loss of the sample. As discussed in sections 2.4.2.5, 3.1.1, and 3.2.1, all cellulosic materials undergo structural collapse upon drying, and this is especially true for CNFs that change from a swollen hydrogel state to a rigid film-like material. This structural collapse has an influence on the swelling ability and accessibility of nanocelluloses, as is evident from experiments attempting to redisperse dried CNFs^1080−1083^ or modify them chemically.^333,413^ In practice, DVS analysis of celluloses (and especially of nanocelluloses), is influenced by the treatment and drying history of the material (e.g., air-dried from water or another solvent,^126^ freeze-dried,^318^ or supercritical drying^1080^). The structural changes affect the moisture sorption and retention in the material, and therefore special care should be devoted to the method of sample preparation to obtain relevant information on its water uptake properties. The main limitation of DVS is that it only provides quantitative assessment of the vapor uptake of a material, with no information about the structural changes that take place during the process such as swelling or changes in viscoelastic properties as is the case with QCM-D.

##### 4.2.3.3 Quartz Crystal Microbalance with Dissipation Monitoring (QCM-D)

QCM-D is a microgravimetry technique which simultaneously measures changes in the resonance frequency and energy dissipation of an oscillating piezoelectric quartz crystal sensor as a result of changes in the mass of the crystal.^1084^ Experiments can be carried out in both liquid and vapor environments, yielding quantitative information on the adsorption of, for example dissolved polymers^1085^ or water vapor, respectively.^271^ QCM-D is a highly sensitive microbalance that detects extremely small changes (<1 ng/cm^2^) in mass of the sensor as a change in the oscillation frequency (Δ*f*).^1084^ For uniform and rigid layers, the change in mass of the sensor (Δ*m*) is proportional to Δ*f*, according to the Sauerbrey equation:^1086^
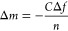
6where *C* is the sensitivity constant of the device (typically *C* ≈ 0.177 mg m^2^ Hz^–1^), and *n* is the measurement harmonic number (*n* = 1, 3, 5, ...).^1087^ The simultaneous monitoring of energy dissipation when the sensor stops oscillating provides a measure of the viscoelastic properties of the film. The dissipation of energy is defined through eq 7:
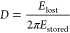
7where *E*_lost_ is the dissipated energy during one cycle of oscillation and *E*_stored_ is the total energy stored in the oscillator.

QCM-D therefore provides quantifiable information on the mass of adsorbed molecules onto an ultrathin model film and also yields information on the changes in the physical properties of the film as a result of adsorption. Indeed, a number of studies have been carried out on model films of regenerated cellulose,^245,1088^ CNCs,^271,279^ and CNFs^280,281,1089^ to better understand the swelling of these materials (Figure 24e,f). It is important to note that the method of film deposition onto the quartz sensors greatly influences film morphology and thereby also its water uptake capacity.^280^ For cellulose solutions, the main techniques of ultrathin film preparation on QCM sensors are spin-coating^1090,1091^ and Langmuir–Schaeffer deposition,^1092,1093^ the former being much faster but yielding films that are not in thermodynamic equilibrium, whereas Langmuir–Schaeffer films, are deposited slowly (one monolayer at a time), allowing for the organization of the cellulose into stable films. Spin-coating has been widely used for the preparation of CNF^281^ and CNC surfaces^271^ but accounts on their adsorption and electrophoretic deposition exist too.^280^ QCM-D has been used in multiple studies to determine the water adsorption capacity of the cellulose following a solvent-exchange protocol, whereby H_2_O is exchanged for D_2_O (originally on regenerated cellulose).^276^ Delepierre et al. used the same technique to measure the bound water in CNC films and reported that a decrease in remnant surface oligosaccharides of different grades of CNCs corresponded to an increase in the water binding capacity.^1094^ Niinivaara et al. surface modified CNCs with oligosaccharides and used the same technique to show that oligosaccharide coated CNCs demonstrated slightly lower water adsorption capacities^528,1094^

#### 4.2.4 Spectroscopic Methods

##### 4.2.4.1 Nuclear Magnetic Resonance Spectroscopy (NMR)

^13^C solid-state NMR has long been recognized as viable tool to determine the degree of crystallinity of celluloses by taking advantage of variations in chemical shifts and ^13^C spin–lattice relaxation time constants in ordered and disordered molecular regions.^1095^ Consequently, the amorphous and crystalline signals of the C4 and C6 carbon appear at a different chemical shift, and their ratio can be used to calculate degree of crystallinity with sufficient accuracy.^1096,1097^ Besides providing information on the molecular order of cellulose, different 1D and 2D solid-state NMR spectroscopy techniques are also used to characterize specific cellulose–water interactions. For instance, signals of surface crystalline domains observed through ^13^C CP/MAS NMR provide qualitative insights into the (irreversible) structural changes upon drying, as a consequence of cellulose microfibril aggregation (i.e., hornification).^105,1098^ As with many of the analytical techniques discussed, sample preparation is of particular importance here to ensure minimal changes to the cellulose crystallinity due to, for example, previous drying, milling, or chemical treatments.^1098,1099^ For example, Newman et al. were able to demonstrate changes within the surface crystalline domains of softwood kraft pulp simply due to wetting and drying cycles.^105^

Both ^1^H and ^2^H (after a hydrogen–deuterium exchange) solid-state NMR experiments can be used to probe water interaction dynamics with nanocelluloses and gain an understanding of the state of the water present (i.e., free or bound water). ^1^H MAS NMR has also been used to investigate water mobility as a function of the moisture content. Vittadini et al. showed that with decreasing relative humidity (<12%), the ^1^H spectra of cellulose show a sharp Lorentzian signal typically observed for the free water component, and a wide Gaussian contribution, which dominated the spectrum at low relative humidity and was attributed to the rigid protons (i.e., low mobility) of the cellulose backbone and bound water.^1100^

**Figure 25:**
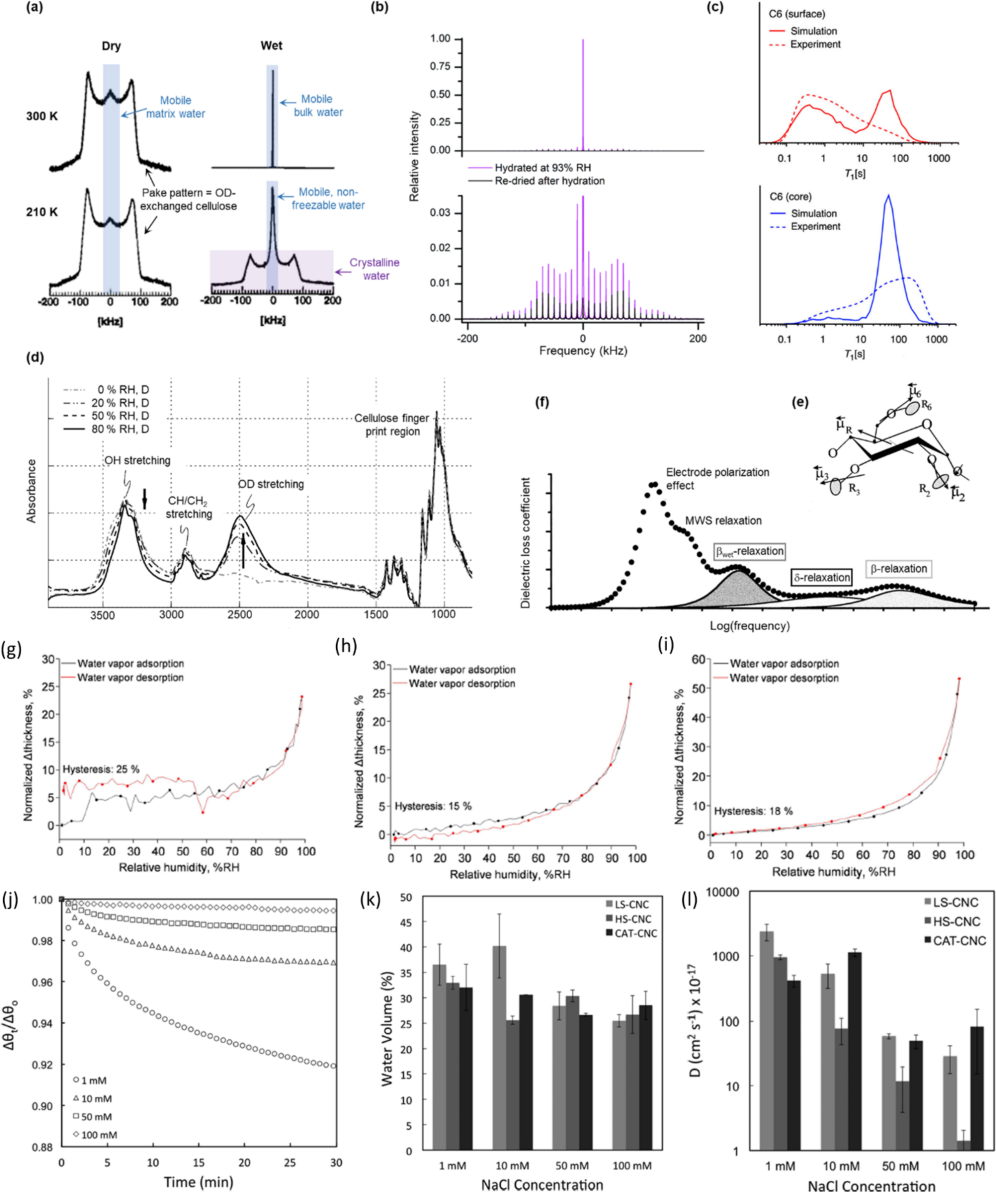
Analysis of cellulose–water interactions by NMR (a–c), IR (d), and dielectric (e,f) spectroscopy, and by spectroscopic ellipsometry (SE, g-i) and surface plasmon resonance spectroscopy (j-l): (a) Solid echo ^2^H NMR spectra of dried and wet cellulose at 210 and 300 K. Transition of the typical Pake pattern (slow ^2^H exchange) to narrow Lorentzian peaks (fast ^2^H exchange).^1102^ (a) Modified with permission fromref (1102). Copyright 1996 American Chemical Society. (b) ^2^H MAS NMR spectra of hydrated and redried MCC in full scale (top) and magnified (bottom) illustrating the signals arising from mobile water (intense central peak) and from the other deuterium-containing sample fractions (SSBs spread over a broad frequency range).^270^ (b) Modified under the terms of CC-BY from ref (270). Copyright 2017 Royal Society of Chemistry. (c) Comparison of experimental and simulated distributions of ^13^C *T*1 relaxation times, normalized to the same value, for C6.^995^ (c) Reproduced with permission from ref (995). Copyright 2019 American Chemical Society. (d) Static FT-IR spectra of spruce cellulose at increasing relative humidity with deuterium vapor.^204^ (d) Modified with permission from ref (204). Copyright 2006 Springer Nature. (e) Dielectric site model of a polysaccharide repeating unit. (f) Principal structure of a dielectric loss spectrum of a wet polysaccharide.^1104^ (e,f) Adapted with permission from ref (1104). Copyright 2001 Elsevier. (g–i) SE adsorption isotherms of (g) 5, (h) 10, and (i) 20 g L^–1^ CNC thin films. Hysteresis of swelling is a measure of the difference between the integrated areas below the adsorption and desorption isotherms.^271^ (g–i) Adapted with permission from ref (271). Copyright 2015 American Chemical Society. (j) Swelling profiles of highly sulfated CNC (HS-CNC) films measured by SPR. Curves are normalized to the angular shift immediately following NaCl solution addition. (k) Calculated volume of water within the CNC films (LS-CNC, low sulfate content CNC; CAT-CNC, cationic CNC) in various concentrations of NaCl after 30 min of swelling determined from SPR profiles. Film porosity (the volume of air in the films initially) is taken as 20%. (l) Diffusion constants calculated for water in CNC films measured from SPR swelling experiments in NaCl solutions.^283^ (j–l) Adapted with permission from ref (283). Copyright 2017 American Chemical Society.

High-resolution ^2^H NMR has significant advantages over ^1^H NMR for identifying different water populations in a cellulose sample as it is (i) selective toward accessible hydroxy groups and (ii) based on the quadrupolar coupling of intramolecular and single-spin properties, thus favoring intramolecular interactions.^270,1101^ Combined, these features enable the identification and quantification of distinct water populations. Less mobile molecules (i.e., those with a slow ^2^H-exchange) give broad, Pake patterned ^2^H NMR spectra, while narrow Lorentzian peaks are typically observed for highly mobile molecules (i.e., those with a rapid ^2^H-exchange) (Figure 25a).^270,1102,1103^^2^H single-pulse experiments are known to result in broad spectral components, making the interpretation of immobile domains at low water contents speculative.^1100^ On the other hand, ^2^H static quadrupolar echo (QE) NMR provides a high-resolution information by refocusing the large quadrupolar broadening, which enables the observation of slow molecular dynamics.^1102^ However, the experiment is restricted by a low signal-to-noise ratio (SNR), which complicates the identification and quantification of the different water populations.^270^

Furthermore, ^2^H MAS NMR has been used to confirm the existence of an immobile and a mobile adsorbed water phase in hydrated cellulose, and to quantify the fraction of each population.^270^ Through magic angle spinning, orientation averaging can be used to remove chemical shift anisotropy and produce spectra resembling that of liquid water with an improved SNR. Such experiments rely mainly on the interpretation of spinning sidebands (SSBs) (i.e., the contribution of immobile water and cellulose ^2^HO) (Figure 25b), which become visible if the sample is spun at a rate less than the magnitude of the anisotropic interaction. The ratio of integrals and line width of the SSBs compared to the central peak (i.e., mobile water) at different relative humidity, thus providing direct quantitative information on the different adsorbed water phases after subtracting the contribution of cellulose deuteroxys. Furthermore, it provides information on the different locations of immobile and mobile adsorbed water on/within the microfibril aggregates. Moreover, ^2^H MAS NMR can provide information on the state of the cellulose hydroxy groups and site specific ^1^H–^2^H exchange.^203^

In addition to 1D NMR experiments, 2D heteronuclear ^1^H–^13^C wide-line separation (WISE) NMR can provide information on water localization and organization in cellulose and cellulose–polymer blends.^1102^ This technique is based on the correlation between a high-resolution ^13^C spectrum with a wide line ^1^H spectrum, where the proton line width refers to the polymer chain dynamics.^1105^ Moreover, the insertion of a spin diffusion time enables direct observation of dipolar couplings between mobile water and cellulose protons, where the spin diffusion coefficient is related to the distance between cellulose and water.^1102^

Another approach to characterize cellulose–water interactions is through the acquisition of spin–lattice (i.e., longitudinal, *T*_1_) and spin–spin (i.e., transverse, *T*_2_) relaxation times using NMR relaxometry.^1101,1106,1107^ The relaxation behavior of nuclei after excitation is a fingerprint in molecular dynamics that can provide information on, e.g., water adsorption. Terenzi et al. used ^2^H transverse and ^13^C longitudinal NMR relaxation measurements to assess the hydration of CNF/xyloglucan nanocomposites.^1108^ The length and width of *T*_2_ as measured by ^2^H NMR provided information on the orientational molecular mobility of water molecules present in the nanocomposites, in addition to water molecule distribution and characteristic length scales, respectively. They demonstrated that a short *T*_2_ can be attributed to slow water mobility and, thus, strong polysaccharide–water interactions.^1108^ On the other hand, *T*_1_, as measured by ^13^C CP/MAS, was used to quantify the difference in molecular mobility between ordered and disordered (including surface) regions in CNF as a function of relative humidity. Chen et al. compared molecular dynamic simulations and experimental relaxation data to quantify nanoscale structure–function relationships of CNFs in a highly hydrated and aggregated state.^995^ This approach enabled the deconvolution of experimental ^13^C NMR *T*_1_ distributions into contributions from both accessible and inaccessible regions of the aggregated CNFs and to distinguish between local C–H dynamics at different carbon sites (i.e., C1, C4, and C6) (Figure 25c). In addition to ^13^C and ^2^H, ^17^O relaxation data has also been used to study cellulose–water interactions.^1101^ Similar to NMR relaxometry, low-field time domain (LF-TD) NMR can also be used to study the dynamic properties of polymer chains from the relaxation of ^1^H spins. Felby et al., for instance, used FT-TD NMR to study cellulose–water interactions during enzymatic hydrolysis.^1109^

##### 4.2.4.2 Fourier Transform Infrared Spectroscopy (FTIR)

FTIR spectroscopy is commonly used to elucidate cellulose–water interactions. Particularly, it is sensitive toward differences in OH conformation and the hydrogen bonds of different cellulose polymorphs,^1110^ even though the exact assignment of the strongly overlapping bands of cellulose hydroxy groups, and the different water populations is a matter of debate.^206^ For example, when observing cellulose–water interactions, strongly bound water is associated with the 3200 cm^–1^ band (−OH stretching range) in the cellulose spectrum, whereas the 3600 cm^–1^ band is associated with loosely bound water that bridges via another water molecule to cellulose.^610^ Moreover, the main spectral regions affected by water adsorption are between 3700 and 3000 cm^–1^ (mainly −OH stretch), 1740–1618 cm^–1^ (−OH bending), and 1190–1139 cm^–1^ (C–O–C asymmetric stretch of the glycosidic linkage).^1110,1111^ Thereby, the signal at ca. 1640 cm^–1^ is very often loosely used as a reference for adsorbed water.^1112−1114^

Different IR instrumentation setups offer various possibilities to characterize cellulose–water interactions. Micro-FTIR is equipped with a light microscope, which allowed for the combined analysis of changes in sample morphology and in situ spectral analysis during water sorption.^1111^ Polarized IR, on the other hand, relies on the polarization dependence of specific IR absorptions of oriented molecules and can be used to unravel the origins of the −OH signals found in the vibrational spectra of crystalline cellulose.^1115^ Hofstetter et al. used dynamic FTIR, which couples dynamic mechanical analysis with step-scan FTIR and has better spectral resolution compared to static FTIR, due to the mechanical extension to investigate the interactions between cellulose and moisture.^204^ Moreover, the authors conducted a controlled ^1^H_2_O–^2^H_2_O (H_2_O–D_2_O) exchange, which provided direct evidence on the accessibility of the different cellulose domains^204,1116^ (Figure 25d).^203,204,206^

##### 4.2.4.3 Dielectric Spectroscopy (DS)

DS is a powerful tool to examine molecular motions of polymeric systems in an extended time span (0.1 ns to 100 s) by probing the dielectric properties of the material.^1117^ Hence, the central prerequisite of this method is the existence of mobile molecular units with a permanent dipole moment or the presence of free mobile charges creating space charges at interfaces.^1118,1119^ In the case of cellulose, for example, only the dipolar sites (Figure 25e) in its specific orientation are of interest.^1119^ The application of DS in cellulose research dates back over a century, and its principles, parametrizations, and experimental aspects are presented in an excellent way by Einfeldt et al.^1104^

In brief, DS measures the response of the dielectric properties of a material in an alternating electric field with a frequency range typically between 10^–6^ and 10^12^ Hz (i.e., broadband dielectric spectroscopy (BDS))^1117^ The latter is of particular relevance for cellulose,^1104^ and in fact cellulose has often been characterized in the low-frequency range, i.e., by dielectric relaxation spectroscopy (DRS), in which the dielectric response is largely dominated by structural effects.^1119^ Consequently, sample preparation for DRS analysis is highly important, and the dielectric response is highly sensitive to water content.^1104^

The response of the sample’s electric dipole moment (i.e., electric polarizability) is directly measured as complex impedance (Z*), giving the real (ε′, dielectric store coefficient) and the imaginary (ε″, dielectric loss coefficient) part of the complex permittivity (ε*(*f*, *T*)).^1104,1117^ Both parameters, ε′ and ε″, are central for the interpretation of cellulose–water systems and are typically recorded and interpreted as a function of the field frequency (*f*) and/or the temperature (*T*).^1018,1120^ Isothermal measurements at varied frequencies are thereby favored because of their possible quantitative interpretation.^1104^ Moreover, if the sample has a noticeable conductivity (e.g., wet samples), conductivity spectra (i.e., σ*(*f*, *T*)) are necessarily discussed.^1018^

As explained by Einfeldt et al., the macroscopic dipolar moment (*Μ*) considers all molecular groups with a permanent dipole moment, which in the case of cellulose is three types of dipolar and movable sites: (i) the pyranose ring (orientational motions around the glycosidic linkage), (ii) the C2/C3 side groups (rotational mobility around the C–O linkage), and (iii) the C6 side group (mobility around the C5–C6 linkage and the C6–O linkage).^1104^ Water bound to cellulose hydroxy groups increases the dipolar moment of the side groups affecting the main polymer chain mobility and produces an additional relaxation process.^1118^ Moreover, the polymeric relaxation, expressed by *Μ* and ε*, is affected by cross-correlation functions of different dipolar moments within the same chain and between neighboring chains, as a consequence of the complex hydrogen bonding network.

Parts e and f of Figure 25 show the different secondary relaxation processes for wet cellulose in the form of a dielectric loss (ε″) spectrum, in which β and β_wet_ relaxations are the most relevant processes. They are observed below the glass transition temperature and in the low frequency range. β relaxations have been related to local motions of the main chain at low temperatures, whereas β_wet_ relaxations are observed in wet samples in the room temperature range^1121^ and are attributed to orientational motions of cellulose and water in a mixed phase.^1118,1119^ The latter disappears completely in dry samples and must be distinguished from σ relaxations which are observed for well-dried polysaccharides in the high temperature range.^1104^ The relaxation strength of β_wet_ relaxations decreases with lower water contents, accompanied by a shift of β_wet_ to lower frequencies, which has been related to the effect of water on the activation energy of local polymer reorientational motions.^1104,1118^

The presence of water in a cellulose sample increases the electric conductivity, which superimposes the dielectric processes of the loss spectrum. This has been associated with the ionization of water, leading to an increase in the number of OH^–^ and H^+^ ions. Thus, the dielectric loss spectrum must be corrected by a simultaneous measurement of the conductivity (σ_0_).^1018,1122^ As consequence of the drastic effect of water on the electric conductivity of cellulose, the investigation of cellulose–water interactions and mixed-phase dielectric processes using DRS is limited to low water contents (max 12–15 wt %). Seemingly, low water contents (<6 wt %) do not affect the high frequency ε′ of cellulose, which is related to bound water that does not contribute to the dielectric response.^1123^

##### 4.2.4.4 Sum Frequency Generation Vibration Spectroscopy (SFG-VS)

SFG-VS is a versatile method for in situ study of molecular arrangement at surfaces and interfaces. SFG is a second-order nonlinear optical process where a tunable pulse infrared (IR) laser beam is mixed by spatial and temporal overlap with a fixed wavelength visible (VIS) laser beam to generate a sum frequency output beam. Because SFG at the phase-matching condition require noncentrosymmetry, it is highly sensitive and specific to surfaces and interfaces as long as the bulk phase is amorphous or centrosymmetric.^1124,1125^ Crystalline cellulose bulk has a noncentrosymmetrical environment, which makes it difficult for SFG to confidently distinguish the OH groups of the surface vs bulk. However, regenerated cellulose models (amorphous cellulose)^1091,1126,1127^ would be a promising substrate for circumventing this problem with cellulose–water interface studies using SFG.

##### 4.2.4.5 Spectroscopic Ellipsometry (SE)

SE is an optical technique which can be utilized to measure the thickness of thin films based on changes in the polarization of light as it is reflected through the film. A beam of single wavelength or spectroscopic light is passed through a polarizer, which is then reflected off the surface of a thin film. Traveling through the film, the light undergoes a change in its elliptic polarization dependent on the thickness (and porosity) of the film.^1128^ The magnitude of the change in polarization can directly be used to model thin film properties such as thickness, refractive index, and extinction coefficient. For cellulosic materials, the classic Cauchy model^1129^ is typically applicable to nonabsorbing transparent films, and as such is commonly used to model cellulose thin films.^280^ SE is a well-established and nonintrusive technique with very high precision and accuracy reaching a resolution in the order of 1 Å for optically uniform and transparent films. However, it is worth mentioning, that ellipsometry data is most meaningful when used in conjunction with complementary characterization techniques such as AFM, QCM-D, or surface plasmon resonance (SPR).^1130^

In terms of nanocellulose–water interactions, SE can be used to monitor changes in the film upon water vapor sorption in situ at the nanoscale (Figure 25g–i).^1128^ Furthermore, because swelling upon water sorption in nanocellulose thin films is restricted only in the lateral dimension, SE can also be used to carry out volumetric analysis.^1131^ For example, Niinivaara et al. unveiled that upon hydration with water vapor, each CNC in a spin-coated film become enveloped in a 1 nm thick layer of adsorbed water (or three monolayers of water molecules), resulting in a two nanometer increase in film thickness for every layer of CNCs within the film as measured by SE.^271^ Hakalahti et al. used SE (and QCM-D) for determination of thickness fractions of water in the TOCNF thin films in different relative humidity values. Thickness fractions of water in the TOCNF thin film were calculated by dividing the thickness increase due to water vapor sorption at a given point along the % relative humidity spectrum by the total thickness of the TOCNF thin film at the same % relative humidity point under the assumption that all changes in thickness or optical thickness occurred due to water vapor uptake. Hence, this method describes the added thickness due to sorption of water, but it does not differentiate between actual thickness of the water molecules and thickness changes in the film due to structural changes like swelling or reconfiguration of individual fibrils or hemicellulose.^282^

##### 4.2.4.6 Surface Plasmon Resonance Spectroscopy

SPR is a surface sensitive optical technique used to measure the adsorption/desorption of molecules or swelling of a thin film deposited onto a surface plasmon active metal substrate. P-polarized light is focused on the surface of the substrate resulting in the generation of a plasmonic wave at the substrate–film interface, the refractive index of which dictates the angle at which the light is coupled into the sensor to excite the plasmons. When a molecule binds (or adsorbs) to the surface of the substrate, or the film swells, the refractive index in the space where the plasmonic wave propagates is changed, as is the surface plasmon resonance angle.^1132,1133^ Multiparameter surface plasmon resonance spectroscopy (MP-SPR) acquires spectra which are dependent on the angle of the incident light in various media^284^ (i.e., gas or liquid) at different wavelengths (i.e., 670 and 785 nm) during a single experiment. From the data, one can deconvolute film thickness and effective refractive index based on optical modeling (using the Fresnel equations).^1134^ Typically, the refractive index of nanocellulose is reported to range from 1.51 to 1.62.^276,284,1135^

To examine the swelling dynamics of CNC thin films, Reid et al. utilized SPR to monitor changes in film thickness as a function of CNC surface chemistry and charge density in various solvents and aqueous environments with different ionic strengths (Figure 25j–l).^283^ The authors found that while the total water uptake capacity of each film was similar due to the restrictions imposed be van der Waals forces between particles, the rate of swelling was highly dependent on CNC surface charge and solvent ionic strength.^283^ SPR has also been frequently used in the studies that include surface modification of nanocellulose films to tailor the interactions between nanocellulose and water. However, in these studies, SPR is mostly used to show the adsorption of the polymer on the surface. For example, Ahola et al.^1136^ and Eronen et al.^1137^ used SPR to surveil the adsorption profile of multiple synthetic and natural polymers on CNF films for the purpose of changing water binding capacity of the material.

#### 4.2.5 Thermal Analysis

##### 4.2.5.1 Differential Scanning Calorimetry (DSC)

DSC is a thermoanalytical technique that measures the energy transferred to or from a sample undergoing a chemical or physical change as a function of temperature. The heat flow profiles attained through a DSC measurement (i.e., DSC curves) can be interpreted to provide detailed information on the enthalpies of transition of a material. When utilized on wet samples at low temperatures, DSC can yield direct information on the pore size distribution of the sample with a method called thermoporosimetry where the Gibbs–Thomson relation yields the relationship between melting point depression and capillary pore diameter. In practice, the sample is frozen, and an increasing temperature ramp detects the melting water in pores of varying size. In terms of cellulose–water interactions, DSC has been intensively investigated for its ability to quantitatively evaluate the different water populations in celluloses, i.e., the contents of free and bound water (section 2.4.2.2).^100,274^

**Figure 26:**
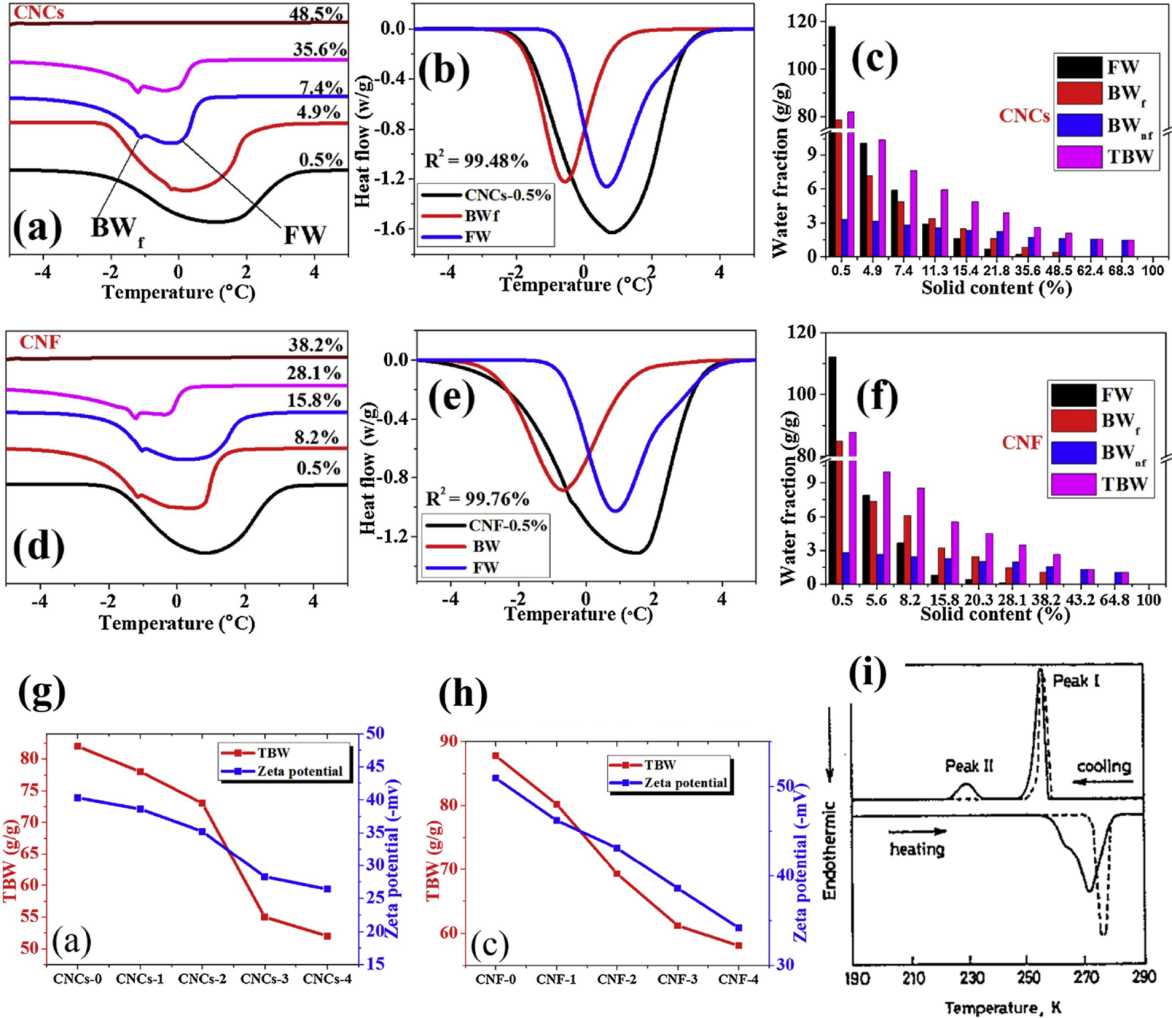
Using DSC to uncover nanocellulose–water interactions. (a,d) Characteristic DSC melting curve for CNCs and CNF. (b,e) Split peak fitting curve of CNCs-0.5% and CNF-0.5%. (c,f) Distribution histograms of various water components in different solids of CNCs and CNF. FW, BW_f_, BW_nf_, and TBW stand for free water, freezing bound water, nonfreezing bound water, and total bound water, respectively.^332^ (g,h) TBW and zeta potential of redispersed nanocellulose dispersion after dehydration. (a–h) Adapted with permission from ref (332). Copyright 2019 Elsevier. (i) Schematic DSC curves of water on different cellulose samples: free water (peak I) and freezing bound water (peak II).^272^ (i) Adapted with permission from ref (272). Copyright 1981 Sage Publications.

The DSC curves of free water within cellulosic materials have similar profiles to that of pure water, indicating similar transition temperatures and enthalpies. Bound water, on the other hand, shows a lower transition temperature than that of pure water due to the restriction caused by the hydroxy groups of cellulose. The enthalpy of melting of freezing bound water adsorbed to cellulose can be quantified by integrating the area under the endothermic DSC curve (Figure 26a–f).^100^ In the case of celluloses, two exothermic peaks of crystallization of adsorbed water are observable during the cooling step: (i) a sharp peak at ca. 255 K for bound water (peak I) and (ii) a broad peak between 230 and 250 K (peak II), indicating free water and freezing bound water in cellulose (Figure 26i).^272^ The melting enthalpy can be plotted against the total water content in the cellulose sample (g water/g dry cellulose sample). Theoretically, the points should fall on a straight line with a slope equal to the heat of fusion of bulk water (Δ*H*_f_ = 79.7 g cal^–1^)^1138^ and an *x*-intercept showing the nonfreezing water content (Δ*H* = 0).^1139^

DSC can yield indirect information on the crystallinity and fiber structure of cellulosic materials,^272,1140^ as well as the accessibility of the cellulose hydroxy groups (Figure 26g,h).^103,1017^ As such, DSC can also help to garner an understanding on the effects of nanocellulose hornification as a result of removal of water.^332^ For instance, Ding et al. implemented DSC to demonstrate a collapse in the mesoporous structure of nanocellulose fibers and a subsequent loss in hydroxy group accessibility of redispersed nanocellulose upon the removal of water.^332^ Furthermore, DSC can also be used to attain information on the pore size distribution of a material such as a wet cellulose nanopaper.^784^ Thermoporosimetry is typically carried out after isothermal heating where temperature is kept constant until the enthalpy of melting has stabilized. This technique assumes that water contained within pores is subjected to elevated pressures and has a higher melting temperature than free water. Hence, the different isothermal melting points obtained during the measurements provide information about the number of pores and their respective size, as reported by Rojo et al.^784^ However, a clear limitation of DSC-based thermoporosimetry are nanocavities smaller than several Å, which cause the formation of nonfreezing water and might hinder an accurate size measurement.^1141^

#### 4.2.6 Rheological and Mechanical Testing

##### 4.2.6.1 Rheology

**Figure 27:**
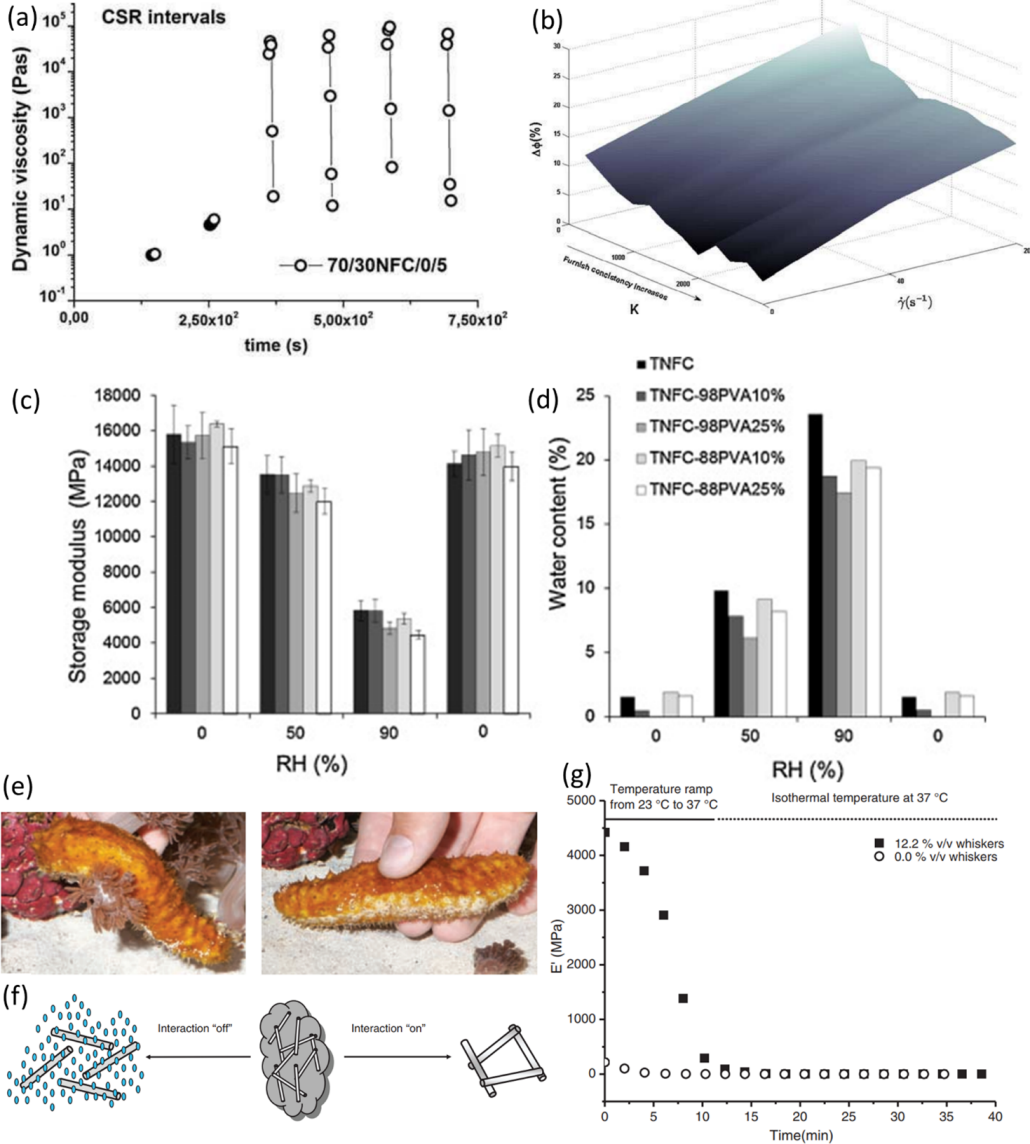
Application of rheology and mechanical measurements in probing nanocellulose–water interactions. (a,b) The influence of shear on the dewatering of high consistency CNF furnishes: (a) Development of the η during the measurement of 5% consistencies CNF-furnish without cellulose fibers, subjected to the rotation rate of 200 s^–1^ during controlled shear rate cycle. (b) Solid content increase Δφ, for different dewatering schemes versus shear rates in controlled shear rate intervals 0, 40, and 200 s^–1^ and the flow index (*K*) for all furnished.^307^ (a,b) Adapted with permission from ref (307). Copyright 2013 Springer Nature. (c,d) Effect of water on mechanical properties of TOCNF/PVA films measured by DMA: (c) Storage moduli and (d) equilibrium water contents of pure TOCNF films and TOCNF–PVA films containing PVA at different RH levels.^759^ (c,d) Adapted with permission from ref (759). Copyright 2015 Elsevier. (e–g) Using DMA to investigate dynamic mechanical properties of stimuli responsive CNC–polymer nanocomposites inspired by sea cucumber dermis. (e) Natural model and bioinspired design of chemomechanical nanocomposites. Pictures of a sea cucumber in relaxed (left) and stiffened (right) state demonstrating the firming of dermal tissue in the vicinity of the contacted area.^687^ (f) Schematic representation of the architecture and switching mechanism in the artificial nanocomposites with dynamic mechanical properties. In the “on” state, strong hydrogen bonds between rigid, percolating CNCs maximize stress transfer and therewith the overall modulus of the nanocomposite. The interactions are switched “off” by the introduction of a chemical regulator that allows for competitive hydrogen bonding.^687^ (g) Time-dependent modulus decrease of neat poly(vinyl acetate) (PVAc) and a 12.2% v/v PVAc/CNC nanocomposite upon immersion into artificial cerebrospinal fluid and increasing the temperature from 23 to 37 °C.^687^ (e–g) Adapted with permission from ref (687). Copyright 2008 The American Association for the Advancement of Science.

The rheological behavior of aqueous nanocellulose suspensions has been studied to determine the effects such as size or morphology, surface chemistry, and aspect ratio, which have been discussed in multiple comprehensive reviews.^3,21,102,219,580,1142^ Furthermore, the flow behavior of aqueous nanocellulose suspensions has been studied in dilute, semidilute, and concentrated regimes, all providing information related to nanocellulose–water interactions. Simply put, the dilute regime typically provides information on the aspect ratio of nanocelluloses and their particle–particle interactions, while the semidilute and concentrated regimes shed light on the viscoelastic properties and structure of suspensions as a function of the shear rate. Additionally, rheological measurements can provide in situ information on the dewatering of nanocellulose suspensions (Figure 27a,b). Dimic-Misic et al. studied the relationship between shear stress-induced dewatering of CNFs and microfibrillated (MFC)-containing furnishes and their rheological behavior.^307,310^ Dynamic dewatering as a function of shear was studied under vacuum in an immobilization cell combined with a rheometer. Dewatering led to an increase in the elastic modulus and viscosity of the suspension as a function of the increase in solids content. The shear-thinning behavior of the cellulosic materials was thereby found to be the central feature influencing shear-induced dewatering and was related to the creation of dewatering channels.^307^ Moreover, the work showed the surface charges of both MFC and CNFs affect both rheological and dewatering properties, which was attributed to fibrillar swelling and nonremovable surface-bound water.^310^

##### 4.2.6.2 Dynamic Mechanical Analysis (DMA)

DMA is a characterization technique used to measure the viscoelastic properties of a material under an oscillating load as a function of oscillation frequency and temperature. While DMA has mostly been used to investigate the effect of water on the performance of (mostly) nanocellulose nanocomposite materials, it can also provide insights into the reversible water-response different types of materials.^687,689^

Utilizing DMA, Hakalahti et al. compared the mechanical properties of TOCNF/poly(vinyl alcohol) (PVA) films in both the dry and wet state to demonstrate that PVA significantly improved the wet stability of the films (Figure 27c,d).^759^ In a different study, Capadona et al. also used DMA to understand the reversible water response of sea cucumber-like CNC reinforced polymer composites (Figure 27e–g).^687^ DMA analysis showed that upon contact changes in composite modulus, elongation at break, and tensile strength were the result of “switching off” the CNC–CNC interactions through water adsorption. Conversely, in the dry state, strong hydrogen bonding between the CNCs maximized the stress transfer and improved the modulus of the nanocomposite. Zhu et al. used DMA to understand the mechanisms behind the shape-memory and the water sensitivity of a CNC/TPUs (thermoplastic polyurethane) nanocomposite. They also evaluated the reversibility of the change in modulus by repeated drying–wetting cycles.^689^

#### 4.2.7 Scattering Techniques

##### 4.2.7.1 Small-Angle Scattering (SAS)

**Figure 28:**
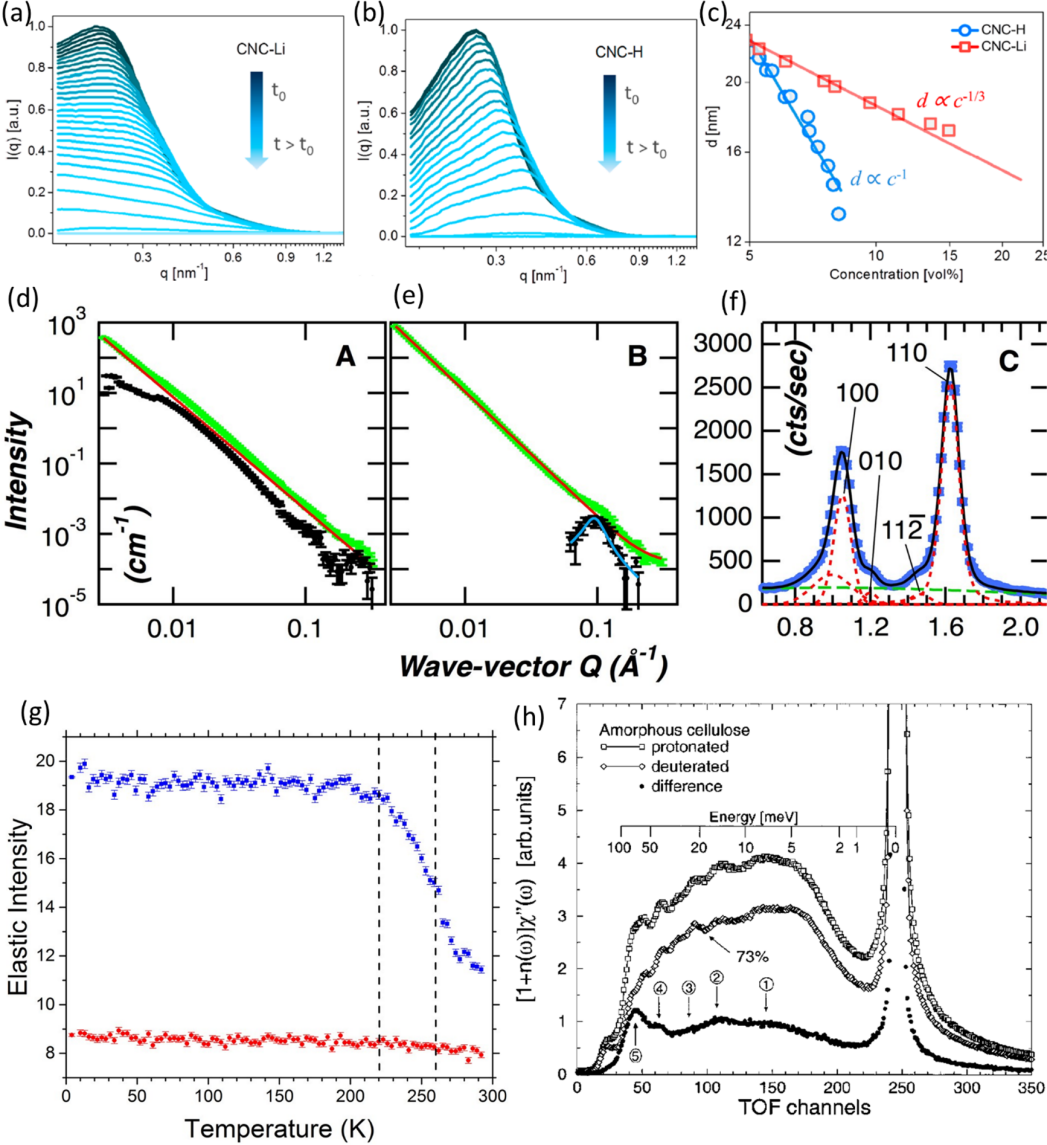
Scattering techniques to elucidate nanocellulose–water interactions. (a–c) Structural properties of CNC dispersions. Time-resolved SAXS data of drying dispersions of (a) CNC-Li and (b) CNC-H over a total time span of 60 and 20 min. (c) Time-dependent change of the center-to-center CNC separation distance *d* with increasing CNC concentrations during evaporation-induced assembly (●, CNC-H; ■, CNC-Li). The solid curves describe a power law relation with exponents of −1/3 and −1.^264^ (a–c) Adapted with permission from ref (264). Copyright 2018 American Chemical Society. (d–f) Analysis of the structure and morphology of dry and hydrated bacterial cellulose by SANS. (d) SANS profile of dry bacterial cellulose. The experimental SANS (green), the power-law fit (red), and the deviation from the power-law behavior (black).^273^ (e) SANS profiles of hydrated bacterial cellulose. The color scheme of the curves is the same as in (d).^273^ (f) Powder X-ray diffraction pattern of bacterial cellulose hydrated in H_2_O. Experimental data (blue), background (green), and peak positions were obtained from data fitting (red).^273^ (g) Using QENS to probe the dynamics of water associated with cellulose. Elastic intensity scans of dry and hydrated deuterated cellulose. The data curves with blue squares and red circles represent the hydrated and dry samples, respectively. The dashed lines denote inflection points in the curves at 220 (nonfreezing bound water) and 260 K (freezing bound water) in the hydrated cellulose sample.^273^ (d–g) Adapted with permission from ref (273). Copyright 2017 Spring Nature. (h) Using INS to study hydroxy accessibility in cellulose. The neutron spectra of amorphous cellulose and the effect of the low-energy dynamics by deuteration of polar (OH) groups. The lower curve (filled circles) is the difference (OH – OD), which essentially reflects the dynamics of OH groups. In this disordered material, all hydroxy (OH) groups that are not completely saturated by hydrogen bonds will be affected by water molecules. Due to the penetration of water molecules, the sample swells and distances between cellulose molecules increase, additionally perturbing the hydrogen bonds between cellulose molecules. This process finally makes almost every hydroxy group accessible to water molecules. Hence, swelling and washing of amorphous cellulose in D_2_O will lead to an exchange of 30% of all hydrogens in the cellulose molecule. On removing heavy water by drying the sample, OD groups are preserved as long as the sample is protected against atmospheric humidity or other protonated polar solvents.^211^ (h) Adapted with permission from ref (211). Copyright 2000 American Chemical Society.

Small-angle X-ray scattering (SAXS) and small-angle neutron scattering (SANS) are able to provide information on the structure and morphology of nanocellulose on a multiscale length,^49,1143−1145^ although data interpretation can be complex. One of the major benefits of small-angle scattering techniques is that changes in sample properties can be measured in real time when exposed to an external force. For example, the alignment of CNCs in aqueous suspension in a relatively weak (0–1.2 T)^1146^ and strong (up to 6.8 T) magnetic field has been monitored in situ using SAXS and SANS, respectively. Furthermore, Håkansson et al. utilized SAXS to detect nanocellulose hydrodynamic alignment in filaments produced using a flow focusing device. This approach gave very detailed information on the dynamics and parameters affecting the aligned assembly of CNF and CNC using such a set up.^1147,1148^ Lastly, the self-assembly of sulfated CNCs upon drying and rewetting was measured in situ by Liu et al. using time-resolved SAXS (Figure 28a–c). They were able to quantitatively follow the nanoscale assembly of CNCs and the reswelling of the dry structure upon hydration as a function of the counterion of the sulfate half ester charge group (hydrogen or lithium).^264^

**Figure 29:**
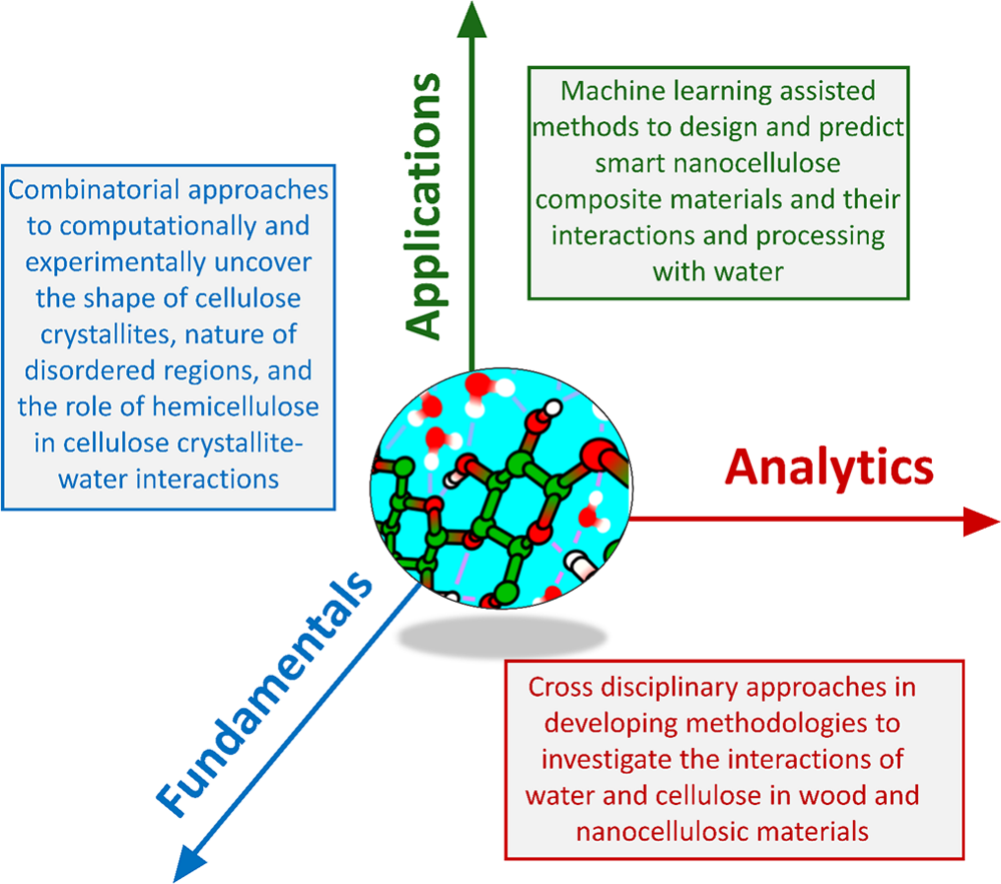
Future trends in the research that encompasses nanocellulose–water interactions.

Using SAXS, Guccini et al.^855^ assessed the influence of atmospheric moisture content on the structure of carboxylated CNF membrane as a function of surface charge and counterion. Strips of the membranes were introduced to a humidity-controlled cell, while the scattering pattern was recorded. The team was able to track the formation of pores and water channels within the nanofiber network in a range of 55–95% relative humidity and concluded that the amount of water absorbed and the swelling increases with the surface charge density of the CNF and decreases with the substitution of sodium with hydrogen ions. Others have also used small-angle scattering to understand the liquid crystalline properties of CNC suspensions as a functions of solids content, total surface charge, and ionic strength,^120,1149,1150^ while fewer reports are available regarding the nematic ordering of CNF suspensions using SAS.^121,266^

Using grazing incident SANS (GISANS), Brett et al. studied the morphological features of ultrasmooth carboxylated CNF thin films as a function of relative humidity. They observed that the size of the three-dimensional CNF aggregates were not affected by an increase in humidity, but their average distance increased as more water is absorbed and condensed into the films.^1140^ Compared to microscopic techniques such as TEM and SEM, GISANS (and GISAXS) have versatile sample environments do not require some degree of electrical conductivity and are able to scan a bigger area with high resolution and fast acquisition rates both parallel and perpendicular to the sample surface. Valencia et al. monitored the effect of water flow during a filtration process across a porous membrane of carboxylated CNFs. Using SAXS, they were able to monitor changes in the porosity of the membranes during filtration and assess the antifouling performance of the membrane.^1151^ An interesting feature specific to SANS is the possibility to contrast-match (or make components “invisible” to the neutron beam) individual components of a multicomponent system by varying the H_2_O/D_2_O ratio, which allows one to study individual characteristics and structural features. O’Neil et al. used this technique in SANS to selectively highlight the effect of hydration on BC surface and to compare the nanostructure and bulk morphology of dry and hydrated BC (Figure 28d–f).^273^

##### 4.2.7.2 Inelastic Scattering (IS)

The dynamics of water in nanocellulose can be directly probed as a function of temperature using inelastic scattering methods as quasielastic neutron scattering (QENS) and inelastic neutron scattering (INS). The information accessible through these techniques are characteristics of a length scale ranging from Ångstroms to a few tens of nanometers level, and the phenomena probed are within the time scales from a fraction of picoseconds to microseconds.^1152^ The results given by INS are resolved according to the change of the kinetic energy of the sample interacting with the neutron beam.

The applications of these techniques on nanocellulose–water systems may not have reached their full potential as there are less reports available compared to other techniques. Nonetheless, Müller et al. investigate the dynamics of water within cellulose using INS, concluding the water accessible sites correspond to the disordered cellulose chains mostly located on the surface of CNFs (Figure 28h).^211^ The peculiarity of QENS is to quantitatively determine the dynamical relaxation processes of water to distinguish between localized rotational and long-range translational motions. This characteristic has been used to determine how the hydration level affect the water dynamics within cellulose and its mechanical properties.^996^ As mentioned in section 4.1, O’Neil et al. detected two populations of water within crystalline BC depending on their relaxation dynamics as a function of temperature, one consisted of surface bound water and the other of confined bound water (Figure 28g).^273^ Guccini et al. were able to detect two types of motions for the water inside a TOCNF network by QENS: one with fast, localized movement of water molecules and the other with slower, diffusive motion. The slower motion was independent of the surface charge of the TOCNFs.^1153^

## 5 Future Trends in Nanocellulose–Water Interactions

**Table 5:** Terms, Abbreviations, Synonyms, and Definitions in Nanocellulose–Water Interaction Research Field

term	abbreviation	definition
nanocellulose		cellulosic materials which have at least one dimension in the nanoscale^119^
cellulose nanocrystal or cellulose nanowhisker	CNC or CNW	highly crystalline, rod-shaped nanoparticles or nanowhiskers produced via hydrolysis of disordered regions of microfibrils^160^
TEMPO-oxidized CNF	TOCNF	cellulose nanofibrils produced via TEMPO mediated oxidation^102^
cellulose nanofibrils or cellulose nanofiber or nanofibrillated cellulose	CNF or NFC	isolated plant cellulose microfibrils^102^
fibrillated nanocellulose		CNF and BC
bacterial cellulose	BC	cellulose produced by bacteria^150^
terminal complex or cellulose synthase complex	TC	cellulose biosynthesis machinery in organisms that produce cellulose^1154^
cellulose microfibrils		smallest supramolecular units of cellulose in nature^58^
cellulose I		native crystalline polymorph of cellulose with parallel chain organization
cellulose II		crystalline polymorph of cellulose with antiparallel chain organization, obtained by mercerization or regeneration of cellulose I^71^
cellulose III		crystalline polymorph of cellulose, obtained by exposing cellulose I or II to liquid ammonia or certain diamines^72^
cellulose I_α_		triclinic polymorph of cellulose I^59^
cellulose I_β_		monoclinic polymorph of cellulose I^59^
free water		free water molecules that fill any voids in cellulosic material’s structure due to capillary forces, swell the matrix, and crystallize at 255 K in DSC^270^
bound water or adsorbed water		bound (adsorbed) water molecules that interact with the cellulosic material’s structure at specific sorption sites. It includes freezing and nonfreezing bound (adsorbed) waters^270^
absorbed water		any water molecule that is taken up by a cellulosic matrix (free water and bound (adsorbed) water)^270^
bulk water		bulk water molecules that surround the cellulosic material’s structure and do not cause observable swelling in the cellulose matrix^275^
freezing bound water		bound (adsorbed) water molecules that behave like bulk water and shows a crystallization peak at about 230–250 K in DSC^272^
nonfreezing bound water		bound (adsorbed) water molecules that do not show crystallization peak in DSC^272^
surface bound water or movable bound water or mobile bound water		bound (adsorbed) bound water molecules, located at the surface of the nanofibril belonging to two different agglomerates in CNF percolated fractal networks that becomes more mobile upon hydration^270^
confined bound water		bound (adsorbed) bound water molecules, located at the surface of the nanofibril belonging to the same agglomerate in CNF percolated fractal networks that is marginally influenced by hydration level^270^
dewatering		partial removal of water from nanocellulosic matrices^898^
drying		removal of (almost) all the water from nanocellulosic matrices^898^
hornification		loss in swelling ability of cellulose upon drying^99^

As implied by the very title of this contribution, the role of water in nanocellulosic materials is considered ambiguous. While many regard water as a problematic component in, e.g., nanopaper or composite structures, it is in fact a necessity in hydrogel-based structures. Much of the potential of water in, for instance, smart materials and energy-related applications entailing nanocellulose has not materialized, and it is the task of the coming years to push the research further in this respect. Perhaps the biggest quest is to merge fundamental knowledge of nanocellulose structures and cellulose–water interactions with design and buildup of new functional materials from nanocellulose. Properties like injectability, responsive behavior, thermal and electrical conductivity, and just chemical modification of cellulose to reach a certain property are strongly linked to water interactions.

Overall, the dominant trend in future is to adhere to the principles of green chemistry more avidly, not just in research but within the whole society, and water-based systems are in a central role in those principles. Because of the vast amount of knowledge that has been gathered over the years on cellulose–water interactions, we know that water is compatible with cellulose and the behavior of cellulose–water systems can be largely, if not fully, predicted. Therefore, processing cellulose in water will need to be the goal even in those cases when ultimately hydrophobic bioproducts are the end goal. These premises will dictate the future development of truly sustainable production and usage of cellulose-based materials. Figure 29 summarizes the future outlook in the field of nanocellulose–water interactions.

## Table of Definitions and Abbreviations

Table 5 summarizes the relevant terms in nanocellulose–water interaction research field, their abbreviations, their synonyms, and definitions.
